# Supplementary material for: Was the risk of death among the population of teachers and other school workers in England and Wales due to COVID-19 and all causes higher than other occupations during the pandemic in 2020? An ecological study using routinely collected data on deaths from the Office for National Statistics
Source: BMJ Open. 2021 Nov 23;11(11):e050656. doi: 10.1136/bmjopen-2021-050656 (PMC8613671; doi:10.1136/bmjopen-2021-050656)
Supplement: Author's manuscript [file bmjopen-2021-050656.draft_revisions.pdf]

BMJ Open is committed to open peer review. As part of this commitment we make the peer review history of every article we publish publicly available.

When an article is published we post the peer reviewers' comments and the authors' responses online. We also post the versions of the paper that were used during peer review. These are the versions that the peer review comments apply to.

The versions of the paper that follow are the versions that were submitted during the peer review process. They are not the versions of record or the final published versions. They should not be cited or distributed as the published version of this manuscript.

BMJ Open is an open access journal and the full, final, typeset and author-corrected version of record of the manuscript is available on our site with no access controls, subscription charges or pay-per-view fees (http://bmjopen.bmj.com).

If you have any questions on BMJ Open's open peer review process please email info.bmjopen@bmj.com

BMJ Open

Risk of death among teachers in England and Wales during the Covid19 pandemic

Journal:	*BMJ Open*
Manuscript ID	bmjopen-2021-050656
Article Type:	Original research
Date Submitted by the Author:	01-Apr-2021
Complete List of Authors:	Lewis, Sarah; University of Bristol, Population Health Sciences<br>Dack, Kyle; University of Bristol, Population Health Sciences<br>Relton, Caroline; University of Bristol, Population Health Sciences<br>Munafo, Marcus; University of Bristol, School of Psychological Science<br>Davey Smith, George; University of Bristol, MRC- Integrative Epidemiology Unit
Keywords:	COVID-19, OCCUPATIONAL & INDUSTRIAL MEDICINE, INFECTIOUS DISEASES

SCHOLARONE™
Manuscripts

I, the Submitting Author has the right to grant and does grant on behalf of all authors of the Work (as defined in the below author licence), an exclusive licence and/or a non-exclusive licence for contributions from authors who are: i) UK Crown employees; ii) where BMJ has agreed a CC-BY licence shall apply, and/or iii) in accordance with the terms applicable for US Federal Government officers or employees acting as part of their official duties; on a worldwide, perpetual, irrevocable, royalty-free basis to BMJ Publishing Group Ltd ("BMJ") its licensees and where the relevant Journal is co-owned by BMJ to the co-owners of the Journal, to publish the Work in this journal and any other BMJ products and to exploit all rights, as set out in our licence.

The Submitting Author accepts and understands that any supply made under these terms is made by BMJ to the Submitting Author unless you are acting as an employee on behalf of your employer or a postgraduate student of an affiliated institution which is paying any applicable article publishing charge ("APC") for Open Access articles. Where the Submitting Author wishes to make the Work available on an Open Access basis (and intends to pay the relevant APC), the terms of reuse of such Open Access shall be governed by a Creative Commons licence – details of these licences and which Creative Commons licence will apply to this Work are set out in our licence referred to above.

Other than as permitted in any relevant BMJ Author's Self Archiving Policies, I confirm this Work has not been accepted for publication elsewhere, is not being considered for publication elsewhere and does not duplicate material already published. I confirm all authors consent to publication of this Work and authorise the granting of this licence.

For peer review only

Risk of death among teachers in England and Wales during the Covid19 pandemic

Sarah J Lewis[1,2], Kyle Dack[1,2], Caroline L Relton[1,2], Marcus R Munafò[1,3] and George Davey Smith[1,2]

1.MRC Integrative Epidemiology Unit at the University of Bristol

2. School of Population Health Sciences, University of Bristol

3. School of Psychological Science, University of Bristol

Corresponding Author: Sarah J Lewis (s.j.lewis@bristol.ac.uk)

Abstract

Objectives: To estimate occupation risk from Covid19 among teachers and others working in schools using publicly available data on mortality in England and Wales.

Design: Analysis of national death registration data from the Office for National Statistics.

Setting: England and Wales, March 20th to 28th December 2020, during the Covid19 pandemic.

Participants: The total working age population in England and Wales plus those still working aged over 65.

Primary and Secondary outcomes: Death with Covid19 as a primary outcome and death from all causes as a secondary outcome.

Results: Across occupational groups there was a strong correlation between Covid19 mortality and both non-Covid19 and all-cause mortality. The absolute mortality rates for deaths with Covid19 were low amongst those working in schools (from 10 per 100,000 in female primary school teachers to 39 per 100,000 male secondary school teachers) relative to many other occupations (range: 10 to 143 per 100,000 in men; 9 to 50 per 100,000 in women).There was weak evidence that secondary school teachers had slightly higher risks of dying with Covid19 compared to the average for all working aged people, but stronger evidence of a higher risk compared to the average for all professionals; primary school teachers had a lower risk. All-cause mortality was also higher amongst all teachers compared to all professionals. Teaching and lunchtime assistants were not at higher risk of death from Covid19 compared with all working aged people.

Conclusion: There was weak evidence that Covid19 mortality risk for secondary school teachers was above expectation, but in general school staff had Covid19 mortality risks which were proportionate to their non-covid mortality risk.

For peer review only

Strengths and limitations of this study

- We used routinely collected data on all deaths in England and Wales; , which are near-complete and not susceptible to serious ascertainment biases.
- We were able to compare mortality data for teachers and other school workers with all other occupational groups and with the working-age population.
- The number of deaths due to Covid19 were small and thus differences between the specific occupational groups were imprecisely estimated.
- We did not have access to individual level mortality data so were not able to account for potential confounders such as comorbidities or household size.
- For those working in school aged over 65 we had neither mortality rates per 100,000 nor total numbers within the group; we only had number of deaths and a 5-year average and we do not know whether the denominators have changed for this group over the last 5 years.

Background

School closures have been implemented in many countries in an effort to slow the spread of the SARS-CoV-2 virus responsible for the Covid19 pandemic. This has affected hundreds of millions of children globally and has been contentious due to harms that have arisen to children as a result of school closures (1). Schools in England and Wales closed to most pupils (except a small number of children of critical workers and some vulnerable children) on the 20th March 2020 and did not reopen fully until September 2020; they closed again for the Christmas holidays (between 11th and 18th December) and reopened on the 8th March in England, although at the time of writing (15th March) secondary schools in Wales are still closed.

Whether or not teachers and others working in schools are at higher risk from Covid19 as a result of schools being open is central to decisions on school reopening, but until recently there has not been good data on this. Two as yet unpublished record linkage studies carried out in Scotland (2) and Finland (3) show that teachers have a greater risk of being diagnosed as a Covid19 case compared with the general population. There was a 25% higher risk in the Scottish study, and between 50% and 70% higher risk in the Finnish study. A study in Sweden found that teachers delivering in person teaching had a two-fold higher risk of Covid-19 infection compared to those who were teaching remotely (4). However, studies examining transmission in schools have found this to occur at low levels (5,6). One reason for the discrepancy is that teachers are likely to be under greater surveillance, and therefore undergo more testing than those working in other occupations. In the Scottish study teachers were much more likely to be tested over the autumn term compared with the general population, and this is likely to explain at least some the elevated number of cases among teachers.

Analyses of more robust outcomes such as hospitalisation from Covid19 are less susceptible to detection bias and may be more reliable. The Scottish teachers study (2) found that 47 out of 11,101 teachers were hospitalised with Covid19 up to 26th November 2020, with an odds ratio (OR) of 0.98 (95% CI 0.67 to 1.45) for hospitalisation compared with controls matched on age, sex and general practice registration and adjusting for ethnicity, deprivation, comorbidity counts and sharing a household with a healthcare worker. Sweden has been an outlier in the Covid19 pandemic in that schools have remained open for children up to 16 years of age. A study by the Swedish Public Health Agency found that 20 out of 103,596 schoolteachers in Sweden received intensive care treatment up to the 30th June 2020, an

For peer review only

age and sex matched analysis comparing teachers with other occupations (except health care workers) reported an OR of 0.43 (95% CI 0.28 to 0.68) (7). Similarly, a study carried out in Norway did not find higher rates of hospitalisation with Covid19 for school teachers, after adjustment for age, sex and country of birth (8). There is an absence of evidence on the risk to teaching and lunchtime assistants working in schools.

The Office for National Statistics (ONS) routinely collect mortality data for those living in England and Wales. They have recently released data on deaths with Covid19 and all-cause mortality by occupation (9). We used this data to compared mortality risk among school workers and all working aged adults in England and Wales. We also compared mortality rates among teachers to all professionals, because there are large differences in mortality risk by occupation, largely driven by differences in socioeconomic circumstances (10).

Methods

The ONS mortality statistics used for this analysis are based on mandatory registration of all deaths in England and Wales. So far 3 datasets on mortality with Covid19 by occupation have been released; the first covered the period from 8th March to the 20th April which included the peak of wave 1 deaths, the second covered the period between 8th March to the 25th May and the final dataset (the one we used for the analysis in this paper) covered the period from 8th March to the 28th December.

We used these data to determine whether the risk of death from Covid19 was higher among teachers and others working in the education sector than those working in other occupations. We compared the number of deaths with historical data on deaths for the same occupational group over 5 years (2015 to 2019) and compared death rates among teachers, teaching and lunchtime assistants death rates among all working aged individuals over the same period of the pandemic. We also compared mortality among teachers with all professionals. Finally, we investigated the ratio of mortality with Covid19 to mortality from other causes among different occupational groups to determine whether school staff were outliers.

Occupational exposure groups

Occupation was reported on the death certificate at the time of death registration by the informant and was coded according to the ONS standard occupational classification 2010 (10). Population counts for occupational groups were estimated from the Annual Population Survey (APS), conducted in 2019 (11). The APS is the largest ongoing household survey in the UK, conducting interviews with members of randomly selected households.

The group ‘teaching and educational professionals’, as defined by the ONS, includes; higher education teaching professionals, further education teaching professionals, secondary education teaching professionals, primary and nursery education teaching professionals, special needs education teaching professionals, senior professionals of educational establishments, education advisers and school inspectors and teaching and other educational professionals not elsewhere defined.

The group ‘teaching and educational professionals’ does not include: teaching assistants, school secretaries, lunchtime assistants or educational support assistants, but these occupations were considered along with teachers working in schools where sufficient data were available.

The group defined by ONS as midday and crossing patrol occupations, includes some individuals who work as road crossing patrols, however not all schools have these and if

For peer review only

they do usually only have one, so the group will mainly comprise of those who supervise children in school during their lunch break and who prepare school lunch. Therefore midday and crossing patrol occupations will be shorted and referred to in this manuscript as lunchtime assistants.

Outcome definition

ONS define deaths with Covid19 as those where Covid19 or suspected Covid19 was mentioned anywhere on the death certificate, including in combination with other health conditions. If a death certificate mentions Covid19, it will not always be the main cause of death but may be a contributory factor.

Statistical analysis

We calculated the relative risk of death for education professions compared to all working aged (20 to 64 year olds) people and compared teachers to all professionals in England and Wales stratified by sex, for this we used published ONS data on total deaths and age adjusted mortality rates per 100,000 for each group. We derived the denominator (total population) for each group of interest as

Population = (total deaths x100000)/mortality rate.

Risk = total deaths/population.

We calculated 95% confidence intervals and *p*-values using the formula;

CI = exp(ln(RR) +/- 1.96 x √(1/deaths + 1/control deaths - 1/population - 1/control population)

P = exp((−0.717 x (RR/SE)) – (0.416 x (RR/SE)²))

The ONS data included deaths for 8 subgroups of educational professionals, we included those with a sufficient number of Covid19 deaths (≥10) in our analysis. In addition, sufficient data were available to include female teaching and lunchtime assistants in this analysis. We also performed a fixed effect meta-analysis across the 4 occupational groups working in schools among females.

We estimated mortality rates for all other causes (other than Covid19) by calculating the difference between all-cause mortality and mortality with Covid19 for each minor occupational group as defined by ONS (12).

We also produced scatterplots of 'all cause' versus 'with Covid19' mortality and 'all other causes' versus 'with Covid19' mortality by occupational group for men and women separately and calculated Pearson's correlation coefficient for these plots.

We also calculated proportionate mortality ratios as mortality rates from Covid19 divided by all-cause mortality for occupations working in schools.

Finally we used our estimates from above for the SE and the denominators for each occupational group to estimate the variance for Covid19 mortality. We then performed a weighted least squares regression analysis (weighted by 1/variance) of Covid19 mortality against other cause to determine the increase in Covid19 for 1 death increase in other cause mortality across occupational groups.

For peer review only

Results

Deaths from Covid19 and all causes among education professionals and others working in schools

Between the 9th March 2020 and 28th December 2020 there were 68,757 deaths from all causes among the working age (20-64 years) population in England and Wales, 7,961 (12%) of these deaths involved Covid19, of which, 2,494 had occurred prior to the 20th April, 2,267 occurred between 20th April and the 25th May, and 3,200 occurred between the 25th May and the 28th December 2020.

There were 1,326 deaths from all causes among educational professionals during this period; 139 of those deaths were thought to involve Covid19.

Table 1 shows that during the 10 months covered by the ONS data all-cause and Covid19 mortality rates among educational professionals were lower than for all working aged adults and similar to those for all professionals.

Covid19 was involved in 1 in 8 male deaths and 1 in 9 female deaths among working aged people; among education professionals these figures were approximately 1 in 8 deaths among males and 1 in 11 among females. Covid19 was involved in a higher proportion of deaths early on in the pandemic; data covering the 6 weeks of the first peak in the UK showed that Covid19 was involved in 1 in 4 deaths in males and 1 in 6 deaths in female education professionals during this time.

We examined number of deaths by occupation among those working in schools aged 20-64 years (Supplementary table S1) and among those aged 65 or older (Supplementary table S2). Among working aged people when deaths across all occupations working in schools were combined, we observed 15% more deaths (excess) compared with the 2015-2019 average for the same period among men and 5% more among women. For women this was less than the excess observed across all occupations and among all professionals; for men it was similar. Deaths with Covid19 appeared to account for almost all the excess among men whereas among women there were twice as many deaths with Covid19 among school workers as there were excess deaths.

Among the older age group there were much higher numbers of deaths compared with their 5-year average (74% more deaths amongst males and 37% among females). However, only 33% of excess deaths in men and 37% in women were thought to involve Covid19. The group of all professionals and of all occupations had fewer excess deaths as a proportion of their 5-year average but a higher proportion of the deaths involved Covid19 compared with those working in schools.

Comparison of Covid19 and all-cause deaths among education professionals and those working in schools compared to the working aged population

Table 2 shows that compared to the total working aged population there was strong evidence that the risk of dying with Covid19 was lower among all education professionals combined (RR_{Males} 0.59, 95%CI 0.46 to 0.75, $RR_{females}$ 0.58, 95%CI 0.46 to 0.73). There was also some evidence of a lower risk of death with Covid19 among female primary and nursery education professionals (RR 0.60, 95%CI 0.38 to 0.93). For both male and female secondary school teachers the relative risk estimate shows a 25% higher risk of death from Covid19, although there was a great deal of uncertainty around these estimates (RR_{Males} 1.25, 95%CI 0.87 to 1.80, $RR_{females}$ 1.26, 95%CI 0.84 to 1.90). There was little evidence of a difference in risk between teaching assistants, or lunchtime assistants compared with

working aged people. A fixed effect meta-analysis across the 4 occupational groups working in schools in women showed that risk was similar to all working aged people (RR 0.95, 95CI 0.77 to 1.14), with weak evidence of heterogeneity across the groups (I^2=55.2%, Chi^2=6.697, p=0.08).

Table 3 shows strong evidence that the risk of death from all causes during this pandemic period was lower for higher and further education teacher professionals compared with the working aged population of England and Wales. The was also evidence that male secondary school teachers (RR 1.26, 95%CI 1.11 to 1.43) and female primary school teachers (RR 1.18, 95%CI 1.06 to 1.32) had slightly higher mortality risk compared to the general population.

We also calculated relative risks for mortality with Covid19 and all-cause mortality in educational professionals versus all professionals (Supplementary Tables S3 and S4). We found strong evidence that both male and female secondary school teachers had a higher risk of mortality with Covid19 compared with all professionals. In addition male and female primary and secondary school teachers had higher mortality risks for all causes compared with all professionals.

Comparison of Covid19 to other cause mortality across occupational groups

Figures 1 and 2 show that there are strong correlations between all other cause mortality and mortality with Covid19. Weighted least squares regression analysis showed that for every death from other causes there were 0.11 (95%CI=0.09 to 0.13, $p=2.0\times10^{-16}$) deaths from Covid19, with no meaningful difference between men (0.11, 95%CI 0.09 to 0.13, $p=2.49\times10^{-12}$) and women (0.10, 95%CI 0.08 to 0.13, $p=4.41\times10^{-9}$).

We also plotted mortality with Covid19 versus all-cause mortality on separate scatterplots (Figures S1 and S2) and found similar results.

Ratio of mortality with Covid19 to other causes

The ratio of deaths with Covid19 to other causes of death, ranged from 1:3 among male nurses and midwifes to 1:13 among male elementary construction occupations (Supplementary Table S5). The ratio of Covid19 to other causes of death for male secondary school teachers was 1:7, which was the same as the average for all male professionals and all working aged men. Among women (Supplementary Table S6), the ratio of mortality with Covid19 to other causes of death, ranged from 1:5 among welfare professionals (social workers and probation officers) to 1:18 among primary and nursery schoolteachers; ratios for female secondary school teachers, teaching assistants and lunchtime assistants were 1:6, 1:10 and 1:8 respectively. Proportionate mortality rates (Covid19 divided by all-cause mortality are presented in Supplementary Table S7.

Discussion

Covid19 deaths among education professionals and others working in schools

We used routinely collected data on mortality for the Office of National Statistics in the UK to study deaths with Covid19 among teachers and other school staff, during the pandemic in 2020. We found that all educational professionals combined had fewer deaths per 100,000 occurring with Covid19 compared to the general population. This is to be expected since the total working aged population of England and Wales will include those who are unable to work due to ill health. The group ‘education professionals’ includes those working in

For peer review only - http://bmjopen.bmj.com/site/about/guidelines.xhtml

universities and school inspectors, many of whom have been working from home throughout the pandemic. We therefore investigated excess deaths specifically among school teachers and other staff working in schools and compared them to all those currently working and all professionals. There were fewer deaths than the 5-year average among female primary and secondary school teachers and the number of deaths among male teachers were similar to the 5-year average. There were more deaths among teaching assistants compared to the 5-year average, but only around half of the excess deaths were thought to involve Covid19. For all women working in schools combined there was a small increase in the number of deaths (5%) compared with the 5-year average, but the number of deaths occurring with Covid19 was greater than the excess, suggesting that some of those dying with Covid19 may have died over the course of a normal year from other causes. For those working in schools who were aged 65 and over there were large excesses in deaths compared with the average for the previous 5 years (74% for men and 37% for women), but only around a third of the excess deaths were thought to involve Covid19. It is possible that some excess deaths, were due to undiagnosed Covid19 among those who did not have classic symptoms (13), but this is less likely for later deaths as the amount of testing for Covid19 increased dramatically over time in the UK. We do not have information on other causes of death within this population over this time period, however in 2019 the most common causes of death among over 65s in England and Wales were heart disease and cancer (14). It is possible that more school staff were dying from other causes during this period due to delayed treatments for other conditions or due to an unwillingness to seek help for fear of contracting Covid19 or of overburdening the healthcare system. It is also possible the number of school staff in this group has increased over the last 5 years.

Relative risk of death with Covid19 among school staff

We found that secondary school teachers had around a 25% higher risk (in both men and women) of dying with Covid19 compared with the general population, although the evidence was weak. There was stronger evidence of an increased risk to secondary school teachers compared to all professionals. However, all-cause mortality was also increased among secondary school teachers compared to all professionals.

Proportionate mortality ratios (mortality from a specific cause: ‘all cause’ or ‘all other cause mortality’) are routinely used in occupational epidemiology in situations where calculation and adequate standardization of mortality rate ratios is not possible (15). The rationale for doing this is that if an occupation has a particular risk for a specific disease the proportion of deaths due to that disease will be increased relative to deaths from other causes in the group. We therefore plotted the correlation between age adjusted mortality for all causes and mortality with Covid19 across minor occupational groups for whom risk was reported in the ONS dataset, the two measures are bound to be correlated to a certain extent because all-cause mortality includes with Covid19. However, we also found a strong positive correlation between mortality with Covid19 and non-Covid19. This is consistent with findings from others which show that deaths from Covid19 closely match ‘normal’ risk within the UK population (13) and many predictors of Covid19 mortality are the same as those for others causes of death (16). Mortality from any cause amongst education professionals and teachers were towards the lower end of the distribution. For male secondary teachers, female teaching and lunchtime assistants the ratio of mortality with Covid19 to all-cause mortality was consistent with other occupational groups;female primary school teachers and female secondary school teachers appeared to be outliers in opposite directions, however the number of deaths in these groups was small and a meta-analysis across occupations working in school showed only weak evidence of heterogeneity..

For peer review only

Consistency with other studies

The data we examined from ONS is consistent with the Public Health Scotland study (2) and with the Swedish study (7) which both showed similar numbers of hospitalisations due to Covid19 among teachers compared with other occupations, but our findings are also compatible with a slightly higher risk of Covid19 mortality among secondary school teachers. Mutambudzi and colleagues (17) investigated the risk of hospitalisation or death by occupation after testing positive for Covid19 among 120,075 working participants (aged 49 to 64 years) in the UK Biobank study, they found weak evidence of more hospitalisations or deaths with Covid19 among education workers compared to non-essential workers up to July 2020 (OR 1.59, 95%CI 0.87 to 2.91) after adjusting for a long list of potential confounders. However, UK Biobank is a highly selected population, which is not representative of the UK population due to a response rate of just 5% (18).

A study of data on teacher absence due to Covid19 in England during the Autumn term (September-December) 2020 found that the proportion of teachers absent due to infection appeared to be similar in both primary and secondary schools (19).The ONS schools infection survey found that primary and secondary staff had similar antibody positivity rates for SARS-CoV2 (15% and 16% respectively) at the end of Autumn term, which reflected exposure during the time when in person teaching was taking place; positivity rates among teachers were slightly lower than among all working aged adults (18%) (20). Despite this, primary school teachers in England and Wales had a lower risk of death with Covid19 compared to secondary school teachers (although confidence intervals were overlapping).

Very few studies have examined risk among teaching assistants and lunchtime assistants as separate groups; we found that despite more deaths occurring in this group in 2020 compared to the previous 5 years, they were not at increased risk of death due to Covid19 or any cause compared to working aged people. A study carried out in California used modelling to compare deaths from all causes during the pandemic and found an odds ratio for teaching assistants of 1.28, but no confidence intervals were provided for that study (21).

Strength and limitations of the study

We used routinely collected data on mortality which includes all deaths in England and Wales, and results are therefore unlikely to be due to ascertainment bias and will be representative of the working aged population of these countries. However, we did not have access to individual level mortality data so were not able to account for potential confounders such as comorbidities or household size. For our relative risk calculations our comparison group also included school staff (although they only made up a very small proportion of the total) because we did not have age adjusted mortality rates excluding teachers. The age adjusted mortality rates calculated by ONS were based on denominators take from a survey conducted in 2019, if the school workforce has changed as a result of the pandemic these rates will be inaccurate, if any changes in the school workforce are systematically different to other professions the results will be biased. Whilst the size of the school workforce in England remained fairly stable between 2015-2019, there was a marked decrease in the number of teachers retiring (from 17,853 teachers retiring in 2015 to 12,062 teachers in 2019) (22), suggesting that the average age of teachers may have increased over this time period. We don’t know what impact the pandemic has had on the size and age distribution of those working in schools, it could be that older more vulnerable staff have retired or conversely staff may have stayed on to help with staff shortages due to teachers having to isolate. Therefore, we were unable to adequately assess the extent to which risk of death had changed during the pandemic period for these occupational groups especially among those aged over 65. In addition, the number of deaths were extremely small and

For peer review only

subject to random fluctuations in some groups. In the period covered by this data many school staff worked remotely for large periods of time during 2020, a survey carried out by the global education company Tes found that just 22% of teachers were engaged in face-to-face teaching during the first lockdown (23),The more vulnerable staff are likely to have worked from home even when schools were fully open, which will have an impact on their workplace exposure.

In Conclusion

Teachers, teaching and lunchtime assistants aged 20-64 years were not at high risk of death relative to the working age population in England and Wales during the Covid19 pandemic. For occupations working in schools Covid19 mortality was generally proportionate to all-cause mortality. Female primary school teachers and female secondary school teachers seemed to be outliers in opposite directions however, evidence for heterogeneity in Covid19 mortality risk among school workers was weak. There were large excesses in deaths from all causes among those working in schools aged over 65, only a third of which involved Covid19 . Further research is needed to determine whether these represent numerator-denominator bias or if there is a true excesses and if so the cause of these excess deaths.

Contributorship statement – SL and GDS conceived the idea for the manuscript and designed the analyses, SL and KD performed the analyses, SL, GDS, CR and MM all contributed to the interpretation of the results, all authors contributed to drafting the manuscript and have seen and approved the final version
Competing interests – The authors have no competing interests.
Funding- Sarah Lewis, Caroline Relton and George Davey Smith have received a grant from National Institute for Health Research (NIHR) and UK Research and Innovation (UKRI)COVID-19 mapping and mitigation in schools (CoMMinS)
Data sharing statement-The data used in this manuscript have been made publicly available by the Office of National Statistics (ONS) in the United Kingdom
Patient and public involvement-Neither patients nor the public were involved in this study.

For peer review only

References

1. Buonsenso, D.; Roland, D.; De Rose, C.; Vásquez-Hoyos, P.; Ramly, B.; Nandipa Chakakala-Chaziya, J.; Munro, A.; González-Dambrauskas, S. Schools Closures during the COVID-19 Pandemic: A Catastrophic Global Situation. *Preprints* 2020, 2020120199 (doi: 10.20944/preprints202012.0199.v1).

2. Fenton L, Gribben C, Caldwell D, Colville S, Bishop J, Reid M, White J, Hutchinson S, Robertson C, Colhoun HM, Wood R, McKeigue PM, McAllister DA. Risk of hospitalisation with COVID-19 among teachers compared to healthcare workers and other working-age adults. A nationwide case-control study. MedRxiv 2021.02.05.21251189 [Preprint]; doi: https://doi.org/10.1101/2021.02.05.21251189

3. Helsinki Gradate School of Economics. Situation Room Report: The Corona Virus and Health Differences – In Which Socioeconomic Groups Have the Most Infections Been Observed in Finland? 19th January, 2021. https://www.helsinkigse.fi/covid19-data-en/situation-room-report-the-corona-virus-and-health-differences-in-which-socioeconomic-groups-have-the-most-infections-been-observed-in-finland/

4. Vlachos J, Hertegård E, Svalery HB. The effects of school closures on SARS-CoV-2 among parents and teachers. Proceedings of the National Academy of Sciences Mar 2021, 118 (9) e2020834118; DOI: 10.1073/pnas.2020834118

5. Ismail SA, Saliba V, Lopez Bernal J, Ramsay ME, Ladhani SN. SARS-CoV-2 infection and transmission in educational settings: a prospective, cross-sectional analysis of infection clusters and outbreaks in England. Lancet Infect Dis. 2020 Dec 8:S1473-3099(20)30882-3. doi: 10.1016/S1473-3099(20)30882-3. Epub ahead of print.

6. Falk A, Benda A, Falk P, Steffen S, Wallace Z, Høeg TB. COVID-19 Cases and Transmission in 17 K–12 Schools — Wood County, Wisconsin, August 31–November 29, 2020. MMWR Morb Mortal Wkly Rep 2021;70:136–140. DOI: http://dx.doi.org/10.15585/mmwr.mm7004e3

7. Ludvigsson JF, Engerström L, Nordenhäll C, Larsson E. Open Schools, Covid-19, and Child and Teacher Morbidity in Sweden. N Engl J Med. 2021 Jan 6:NEJMc2026670. doi: 10.1056/NEJMc2026670. Epub ahead of print. https://www.nejm.org/doi/full/10.1056/NEJMc2026670

8. Magnusson K, Nygård K, Vold L, Telle K. Occupational risk of COVID-19 in the 1st vs 2nd wave of infection. MedRxiv *Preprints* https://www.medrxiv.org/content/10.1101/2020.10.29.20220426v1.full.pdf

9. Coronavirus (COVID-19) related deaths by occupation, England and Wales. Release date 25th January 2021. https://www.ons.gov.uk/peoplepopulationandcommunity/healthandsocialcare/causesofdeath/datasets/coronaviruscovid19relateddeathsbyoccupationenglandandwales

10. Katikireddi SV, Leyland AH, McKee M, Ralston K, Stuckler D. Patterns of mortality by occupation in the UK, 1991-2011: a comparative analysis of linked census and mortality records. Lancet Public Health. 2017 Oct 23;2(11):e501-e512. doi: 10.1016/S2468-2667(17)30193-7.

11. Office for National Statistics. SOC2010 volume 1: structure and descriptions of unit groups.

For peer review only

https://www.ons.gov.uk/methodology/classificationsandstandards/standardoccupationalclassificationsoc/soc2010/soc2010volume1structureanddescriptionsofunitgroups

12. Office for National Statistics. Annual population survey (APS) QMI. https://www.ons.gov.uk/employmentandlabourmarket/peopleinwork/employmentandemployeetypes/methodologies/annualpopulationsurveyapsqmi
13. Spigelhalter D. Use of “normal” risk to improve understanding of dangers of covid-19. BMJ 2020; 370: m3259. https://doi.org/10.1136/bmj.m3259
14. Office for National Statistics. Deaths registered in England and Wales. Release date July 2020. https://www.ons.gov.uk/peoplepopulationandcommunity/birthsdeathsandmarriages/deaths/datasets/deathsregisteredinenglandandwalesseriesdrreferencetables
15. Bhaskaran K, Bacon SCJ, Evans SJW, Bates CJ, Rentsch CT, MacKenna B, Tomlinson L, Walker AJ, Schultze A, Morton CE, Grint D, Mehrkar A, Eggo RM, Inglesby P, Douglas IJ, McDonald HI, Cockburn J, Williamson EJ, Evans D, Curtis HJ, Hulme WJ, Parry J, Hester F, Harper S, Spiegelhalter D, Smeeth L, Goldacre B. Factors associated with deaths due to COVID-19 versus other causes: population-based cohort analysis of UK primary care data and linked national death registrations within the OpenSAFELY platform. MedRxiv *Preprints* https://www.medrxiv.org/content/10.1101/2021.01.15.21249756v2.full.pdf
16. Decouflé P, Thomas TL, Pickle LW. Comparison of the proportionate mortality ratio and standardized mortality ratio risk measures. Am J Epidemiol. 1980 Mar;111(3):263-9. doi: 10.1093/oxfordjournals.aje.a112895.
17. Mutambudzi M, Niedwiedz C, Macdonald EB, et al. Occupation and risk of severe COVID-19: prospective cohort study of 120 075 UK Biobank participants. Occupational and Environmental Medicine Published Online First: 09 December 2020. doi: 10.1136/oemed-2020-106731. https://oem.bmj.com/content/early/2020/12/01/oemed-2020-106731.full#DC1
18. Tyrrell J, Zheng J, Beaumont, R. *et al.* Genetic predictors of participation in optional components of UK Biobank. *Nat Commun* **12,** 886 (2021). https://doi.org/10.1038/s41467-021-21073-y
19. Southall E, Holmes A, Hill EM, Atkins BD, Leng T, Thompson RN, Dyson L, Keeling MJ, Tildesley MJ. An analysis of school absences in England during the Covid-19 pandemic. medRxiv 2021.02.10.21251484 [Preprint]; doi: https://doi.org/10.1101/2021.02.10.21251484
20. Office for National Statistics. COVID-19 Schools Infection Survey Round 2, England: December 2020. https://www.ons.gov.uk/peoplepopulationandcommunity/healthandsocialcare/conditionsanddiseases/bulletins/covid19schoolsinfectionsurveyround2england/december2020#main-points

For peer review only

21. Chen Y-H, Glymour M, Riley A, Balmes J, Duchowny K, Harrison R, Matthay E, Bibbins-Domingo K. Excess mortality associated with the COVID-19 pandemic among Californians 18–65 years of age, by occupational sector and occupation: March through October 2020. medRxiv 2021.01.21.21250266 [Preprint]; doi: https://doi.org/10.1101/2021.01.21.21250266

22. United Kingdom Government. Reporting Year 2019. School workforce I n England. https://explore-education-statistics.service.gov.uk/find-statistics/school-workforce-in-england#dataBlock-4189d4c5-ba35-4f48-9248-cc39f53703a2-tables

23. Tes. Reality of lockdown for school staff: 5 key findings. https://www.tes.com/news/reality-lockdown-school-staff-5-key-findings

For peer review only

Table legends

Table 1 Age adjusted all-cause and with Covid19 mortality per 100,000 population (95% confidence intervals) taken from 3 ONS datasets on Covid19 mortality by occupation covering different time periods during the 2020 Covid19 pandemic. Incudes the ratio of Covid19 deaths to all-cause deaths for education professionals, all professionals and all working aged people (aged 20-64 years) stratified by sex.

Table 2 Relative risks for death occurring with Covid19 for school staff aged 20-64 years compared to the total working aged population, calculated using age-adjusted mortality rates by occupation between 9th March and 28th December 2020 from the ONS dataset.

Table 3 Relative risks for death occurring from all causes for school staff aged 20-64 years compared to the total working aged population, calculated using age-adjusted mortality rates by occupation between 9th March and 28th December 2020 from the ONS dataset.

Figure legends

Figure 1 Scatterplot of mortality rate (per 100,000) for Covid19 and all other causes of death for men, using age adjusted mortality rates between 9th March and 28th December 2020 from ONS. Pearson's correlation coefficient = 0.78, $p = 1.4 \times 10^{-11}$

Figure 2 Scatterplot of mortality rate (per 100,000) for Covid19 and all other causes of death for women, using age adjusted mortality rates between 9th March and 28th December 2020 from ONS. Pearson's correlation coefficient = 0.88, $p = 1.7. \times 10^{-09}$

For peer review only

For peer review only

Table 1 Age adjusted all cause and with Covid19 mortality per 100,000 population (95% confidence intervals)

Occupational group	Cause of Mortality	Males			Females		
		8th March-20th April	8th March-25th May	8th March-28th December	8th March-20th April	8th March-25th May	8th March-28th December
Working aged adults	With Covid19	9.9 (9.4-10.4)	19.1 (18.4-19.8)	31.4 (30.6-32.3)	5.2 (4.9-5.6)	9.7 (9.3-10.2)	16.8 (16.2-17.5)
	All cause	41.6 (40.6-42.6)	78.1 (76.7-79.4)	256.0 (253.5-258.4)	26.3 (25.5-27.1)	48.4 (47.3-49.4)	158.3 (156.4-160.2)
	Ratio*	1:4.2	1:4.1	1:8.2	1:5.1	1:5.0	1:9.4
Professional Occupations	With Covid19	5.6 (4.6-6.6)	11.6 (10.2-13.0)	17.6 (15.9 - 19.3)	4.2 (3.3-5.2)	8.0 (6.8 to 9.3)	12.8 (11.2- 14.4)
	All cause	22.2 (20.2-24.1)	41.2 (38.5-43.8)	130.4 (125.7-135.2)	22.0 (19.9-24.1)	39.3 (36.5-42.1)	120.0 (115.2-124.9)
	Ratio*	1:4	1:3.6	1:7.4	1:5.2	1:4.9	1:9.4
Educational professionals	With Covid19	6.7 (4.1-10.3)	12.9 (9.3-17.4)	18.4 (14.0-23.6)	3.3 (2.0-4.9)	6.0 (4.2-8.1)	9.8 (7.5-12.5)
	All cause	28.0 (22.4-34.5)	48.1 (40.4-55.7)	153.4 (139.8-167.1	20.4 (16.9-24.0)	37.2 (32.5-42.0)	110.0 (101.9-118.2)
	Ratio*	1:4.2	1:3.7	1:8.3	1:6.2	1:6.2	1:11.2

*Ratio of deaths involving Covid19 deaths to all deaths

For peer review only

Table 2 Covid19 deaths for educational professions aged 20-64 years compared to all working aged people, between 9th March and 28th December 2020

Description	Men					Women				
	Risk*	Risk for all workers	RR	95% CI	P-value	Risk*	Risk for all workers	RR	95% CI	P-value
All educational professionals	0.00018	0.00031	0.59	0.46 - 0.75	0.000018	0.000098	0.00017	0.58	0.46 - 0.73	0.0000058
Higher education teaching professionals	0.00012	0.00031	0.37	0.20 - 0.68	0.0016	NA[1]				
Further education teaching professionals	0.00025	0.00031	0.79	0.42 - 1.46	0.46	NA[1]				
Secondary education teaching professionals	0.00039	0.00031	1.25	0.87 - 1.80	0.23	0.00021	0.00017	1.26	0.84 - 1.90	0.27
Primary and nursery teaching professionals	NA[1]					0.00010	0.00017	0.60	0.38 - 0.93	0.024
Teaching assistants	NA[1]					0.00015	0.00017	0.89	0.65 - 1.23	0.5
Lunchtime assistants	NA[1]					0.00019	0.00017	1.14	0.72 - 1.82	0.58

*Total deaths divided by denominator [1]Less than 10 deaths, rate not reported.

For peer review only

Table 3 All-cause mortality for school staff aged 20-64 years compared to all working aged people, between 9th March and 28th December 2020

Description	Men					Women				
	Risk*	Risk for all workers	RR	95% CI	P-value	Risk*	Risk for all workers	RR	95% CI	P-value
All educational professionals	0.0015	0.0026	0.60	0.55 - 0.65	1.74×10^{-29}	0.0011	0.0016	0.69	0.65 - 0.74	6.14×10^{-23}
Higher education teaching professionals	0.00095	0.0026	0.37	0.30 - 0.47	2.00×10^{-16}	0.00073	0.0016	0.46	0.35 - 0.61	1.13×10^{-07}
Further education teaching professionals	0.0017	0.0026	0.66	0.52 - 0.84	0.00077	0.00096	0.0016	0.61	0.47 - 0.79	0.00015
Secondary education teaching professionals	0.0032	0.0026	1.26	1.11 - 1.43	0.00033	0.0015	0.0016	0.92	0.78 - 1.07	0.28
Primary and nursery teaching professionals	0.0024	0.0026	0.94	0.66 - 1.33	0.72	0.0019	0.0016	1.18	1.06 - 1.32	0.0023
Teaching assistants	0.0017	0.0026	0.65	0.45 - 0.95	0.024	0.0017	0.0016	1.05	0.95 - 1.16	0.3
Lunchtime assistants	NA[1]					0.0018	0.0016	1.14	0.98 - 1.33	0.08

*Total deaths divided by denominator [1]Less than 10 deaths, rate not reported.

For peer review only

For peer review only

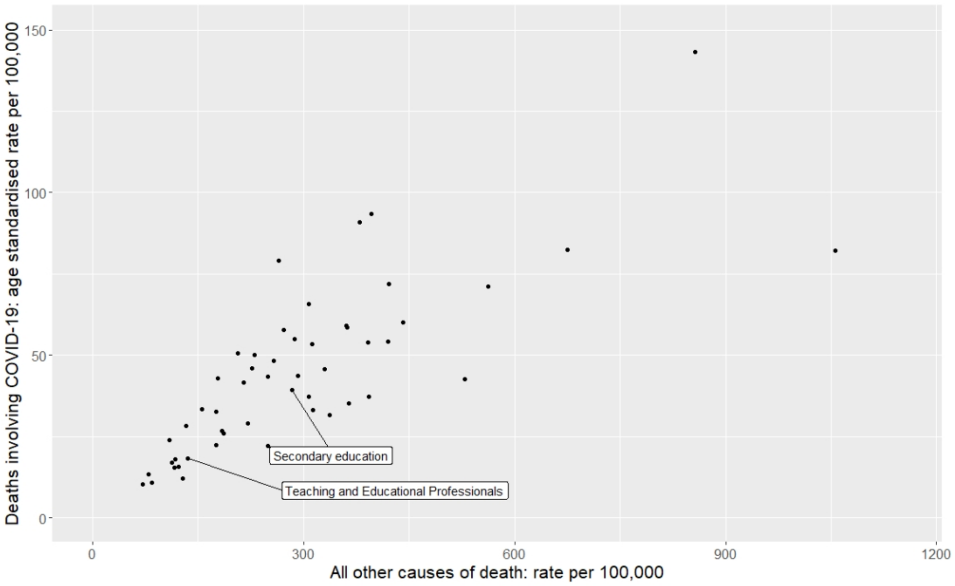

Figure 1 Scatterplot of mortality rate (per 100,000) for Covid19 and all other causes of death for men, using age adjusted mortality rates between 9th March and 28th December 2020 from ONS. Pearson's correlation coefficient = 0.78, p = 1.4 × 10^{-11}

317x202mm (96 x 96 DPI)

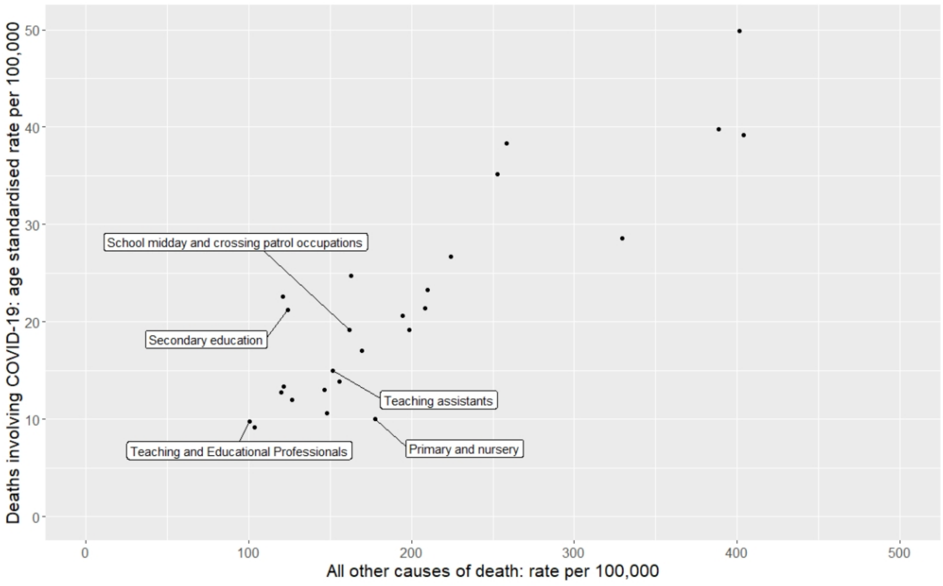

Figure 2 Scatterplot of mortality rate (per 100,000) for Covid19 and all other causes of death for women, using age adjusted mortality rates between 9th March and 28th December 2020 from ONS. Pearson's correlation coefficient = 0.88, p = 1.7. $\times 10^{-09}$

317x202mm (96 x 96 DPI)

Supplementary material

Deaths occurring among those working in schools in 2020 compared with the previous 5 years.

Table S1 shows a breakdown of the number of deaths by occupation in 2020 and the average for the previous 5 years among those working in schools aged 20-64 years, also includes figures for all occupations and all professionals for comparison. The number of excess deaths was calculated by subtracting the 5 year average number of deaths (provided by ONS) from the number of deaths from all causes between 9th March and 28th December 2020 for each occupation and occupational group. The number of excess deaths was converted into a percentage of the 5-year average (percentage excess). The percentage excess explained by Covid19 was calculated by dividing the number of Covid19 deaths by the number of excess deaths and multiplying this by 100. We set the maximum percentage excess deaths to be 100, but we did not cap the percentage excess explained by Covid19. The reason for this is to determine whether Covid19 could be a factor in some deaths which may have occurred anyway.

The number of deaths in the individual groups are mostly small and it is difficult to draw conclusions from these as they are imprecisely estimated. However, among female teachers there were fewer deaths than the 5-year average, and among male teachers deaths were 3% higher. Teaching assistants appeared to have a high number of deaths compared with the 5-year average, although only around half of these involved Covid19. When deaths across all occupations working in schools were combined, we observed 15% more deaths (excess) compared with the 2015-2019 average for the same period among men and 5% more among women. For women this is less than the excess observed across all occupations and among all professionals; for men it is similar. Deaths with Covid19 appeared to account for almost all the excess among men whereas among women there were twice as many deaths with Covid19 among school workers as there were excess deaths.

Table S2 shows a breakdown of the number of deaths by occupation among those working in schools aged 65 and older. Excess deaths and percentage of excess deaths explained by Covid19 were calculated as for Table 1s above. This age group had much higher numbers of deaths compared with their 5-year average than the younger age group. Amongst all males in this age group there were 74% more deaths and amongst females there were 37% more than the 5-year average. However, only around a third of the excess deaths were thought to involve Covid19. The group of all professionals and of all occupations had fewer excess deaths as a proportion of their 5-year average but a higher proportion of the deaths involved Covid19 compared with those working in schools.

We did not have the denominators (total number working in each occupational group) for the figures presented in Tables 1s and 2s, so we were unable to work out the uncertainty in these estimates, and this will be very large in some of the smaller occupational groups**.**

Comparison of risk of death with Covid19 and from all causes for education professionals versus all professionals

Table S3 shows relative risk of deaths occurring with Covid19 for educational professionals aged 20-64 years compared to all professionals, between 9th March and 28th December 2020. Risk of dying with Covid19 in males varied from 11.5 (95%CI 5.2 to 21.7) in 100,000 for professionals in higher education to 39.2 (95%CI 24.3 to 58.6) in 100,000 for secondary

school teachers. Similarly, risk in females ranged from 9.8 (95%CI 7.5 to 12.5) in 100,000 for higher education professionals to 21.2 (95% CI 12.4 to 33.2) in 100,000 among secondary school teachers. Compared with all professionals there was strong evidence that the risk of dying with Covid19 was elevated for male (RR 2.23, 95%CI 1.54 to 3.22) and female (RR 1.66, 95%CI 1.09 to 2.51) secondary school teachers. There was also evidence that the risk of dying with Covid19 was reduced among female higher education professionals (RR 0.77, 95%CI 0.60 to 0.97). For other analyses the confidence intervals were wide, and it was not possible to draw firm conclusions, although there was a suggested reduction in risk for female primary school teachers and male further education teachers.

Table S4 shows relative risks for all-cause mortality for educational professionals aged 20-64 years compared to the total working aged population, between 9th March and 28th December 2020. Both Male and Female higher education professionals had lower risks of mortality compared with the group of all professionals. However, there was very strong evidence that primary and secondary school teachers had higher risk of mortality from all causes compared with all professionals. This was especially true for males; male secondary school teachers had a 2.5 fold increased risk of mortality compared with all professionals and male primary school teachers a 1.8 fold increased risk.

Ratio of mortality with Covid19 versus other causes

We estimated the mortality due to all causes except Covid19 (Age adjusted all-cause mortality minus mortality with Covid19) and calculated the ratio of Covid19 mortality to mortality from other causes for minor occupational groups (according to ONS) and also for secondary school teachers and female primary school teachers. These figures are shown in Tables 3s and 4s.

Table S5 shows the age adjusted mortality rates per 100,000 individuals for deaths with Covid19, plus our estimate of the non-Covid19 mortality per 100,000 among men. The ratio of deaths with Covid19 to other causes of death, ranged from 1 to 3 among male nurses and midwifes to 1 to 13 among elementary construction occupations. The ratio for male secondary school teachers was 1 death with Covid19 to 7 other deaths, which was similar to that for all education professionals.

Table S6 shows the age adjusted mortality rates per 100,000 individuals for deaths with Covid19, plus our estimate of the non-Covid19 mortality per 100,000 among women. The ratio of deaths with Covid19 to other causes of death, ranged from 1 to 5 among welfare professionals (social workers and probation officers) to 1 to 18 among primary and nursery school teachers. The ratio for female secondary school teachers was 1 death with Covid19 to 6 other deaths. Therefore, Covid19 was less likely to be a factor in deaths among female primary and nursery school teachers than other professions and Covid19 was more likely to primary and nursery school teachers than other professions and more likely to be involved in deaths among females secondary school teachers than among other professions except probation officers and social workers. For female teaching assistants the ratio of mortality with covid to other causes was 1:10 for school midday and crossing patrol occupations this was 1:8.

Note: There were fewer occupational groups with useable data (sufficient numbers of deaths to have age-adjusted mortality risks) for women than for men, this is due to small numbers of deaths in some groups among women.

Table S7- We calculated the proportionate mortality rates for occupational groups working in schools for which we had sufficient numbers of Covid19 deaths (n>10). This was calculated by dividing the number of deaths involving Covid19 for the occupational group by the total number of deaths in that group. We also calculated the SE of the proportion and from that the confidence intervals for the proportion using standard formula.

We found that male secondary school teachers had a proportionate mortality rate which was very similar to all males currently in work. We found that female teaching assistants and females lunchtime assistants had proportionate mortality rates which were similar to all other females currently in work. Female secondary school teachers had a proportionate mortality rate for Covid19 which was around 50% higher than for all female workers but the confidence intervals around this estimate were wide and overlapped with the estimate for all other workers. The proportionate mortality rate for female primary school teachers was 40% lower than for all female workers and the confidence intervals for this estimate did not overlap with those for all female workers.

Figure S1 and **S2** show scatterplots of mortality with Covid19 versus mortality from all causes for minor occupational among men (1s) and among women (2s). There was a very strong correlation between mortality with Covid19 and all-cause mortality among both men and women, correlation coefficients were 0.83 and 0.91 among men and women respectively with very small p-values. Risk of both Covid19 mortality and all-cause mortality among teachers and school staff were lower end of the scale. Male secondary school teachers fell within the centre of the plot, as did female teaching assistants and school midday and crossing patrol occupations, suggesting that the Covid19 risk reflects their normal mortality risk. However, both female primary and nursery school teachers and female secondary school teachers were slight outliers but in opposite directions, female primary and nursery school teachers had a low Covid19 risk relative to their all-cause mortality risk and female secondary school teachers had a slightly higher Covid19 risk relative to their all-cause mortality risk.

For peer review only

Table S1 Deaths among working aged (20-64) people who working in education in England and Wales 9th March to 28th December 2020

Description	Men						Women					
	Deaths involving COVID-19	All causes of death	All causes 5 year average	Excess	% excess	Excess due to Covid19	Deaths involving COVID-19	All causes of death	All causes 5 year average	Excess	% excess	Excess due to Covid19
Secondary education teaching professionals	29	241	233	8	3%	100%	23	156	173	-17	-10%	NA
Primary and nursery education teaching professionals	4	31	30	1	3%	100%	19	327	368	-41	-11%	NA
Special needs education teaching professionals	1	12	8	4	50%	25%	3	27	33	-6	-18%	NA
Teaching and other educational professionals n.e.c.	8	56	32	14	44%	57%	7	70	61	9	15%	78%
School secretaries	0	3	2	1	50%	0%	4	58	53	5	9%	80%
Teaching assistants	5	28	19	9	47%	56%	37	396	307	89	29%	42%
Educational support assistants	1	7	8	-1	-13%	NA	3	55	58	-3	-5%	NA
School midday and crossing patrol occupations	2	11	5	6	120%	33%	18	169	149	20	13%	90%
All those working in schools	50	389	337	52	15%	96%	114	1258	1202	56	5%	204%
All professional occupations	419	3144	2765	379	14%	111%	279	2696	2477	219	9%	127%
All occupations	4225	33904	29745	4159	14%	102%	1742	18419	16528	1891	11%	92%

For peer review only

Table S2 Deaths among over 65s who are working in education in England and Wales 9th March to 28th December 2020

Description	Men						Women					
	Deaths involving COVID-19	All causes of death	All causes 5 year average	Excess	% excess	Excess due to Covid19	Deaths involving COVID-19	All causes of death	All causes 5 year average	Excess	% excess	Excess due to Covid19
Secondary education teaching professionals	77	565	327	238	73%	32%	19	306	226	80	35%	24%
Primary and nursery education teaching professionals	10	76	45	31	69%	32%	52	709	495	214	43%	24%
Special needs education teaching professionals	4	30	7	23	329%	17%	6	60	40	20	50%	30%
Teaching and other educational professionals n.e.c.	9	49	29	20	69%	45%	11	98	60	38	63%	29%
School secretaries	1	3	3	0	0%	NA	13	99	88	11	13%	118%
Teaching assistants	1	14	12	2	17%	50%	31	268	221	47	21%	66%
Educational support assistants	1	6	5	1	20%	20%	13	64	48	16	33%	81%
School midday and crossing patrol occupations	4	13	6	7	117%	57%	46	325	231	94	41%	49%
All those working in schools	107	756	434	322	74%	33%	191	1929	1409	520	37%	37%
All professional occupations	637	4504	3273	1231	38%	52%	401	3480	2712	768	28%	52%
All occupations	7033	43640	34676	8964	26%	78%	2993	25277	19842	5435	27%	55%

For peer review only

Table S3 - Covid19 related deaths for educational professionals aged 20-64 years compared to all professionals, between 9th March and 28th December 2020

Description	Men					Women				
	Risk	Risk for all professionals	RR	95% CI	P-value	Risk	Risk for all professionals	RR	95% CI	P-value
All educational professionals	0.00018	0.00018	1.05	0.82 - 1.34	0.74	0.000098	0.00013	0.77	0.6 - 0.97	0.03
Higher education teaching professionals	0.00012	0.00018	0.65	0.35 - 1.22	0.18	NA[1]				
Further education teaching professionals	0.00025	0.00018	1.40	0.75 - 2.62	0.29	NA[1]				
Secondary education teaching professionals	0.00039	0.00018	2.23	1.54 - 3.22	0.000025	0.00021	0.00013	1.66	1.09 - 2.51	0.017
Primary and nursery teaching professionals	NA[1]					0.00010	0.00013	0.78	0.49 - 1.23	0.29

[1]Less than 10 deaths, rate not reported in ONS data.

Table S4 – All cause mortality for education professionals aged 20-64 years compared to all professionals, between 9th March and 28th December 2020

Description	Men					Women				
	Risk	Risk for all professionals	RR	95% CI	P-value	Risk	Risk for all professionals	RR	95% CI	P-value
All educational professionals	0.0015	0.0013	1.18	1.08 - 1.28	0.00023	0.0011	0.0012	0.92	0.85 - 0.98	0.015
Higher education teaching professionals	0.00095	0.0013	0.73	0.58 - 0.92	0.007	0.00073	0.0012	0.61	0.46 - 0.81	0.00059
Further education teaching professionals	0.0017	0.0013	1.29	1.01 - 1.65	0.039	0.00096	0.0012	0.80	0.62 - 1.04	0.092
Secondary education teaching professionals	0.0032	0.0013	2.48	2.18 - 2.81	1.41×10^{-40}	0.0015	0.0012	1.21	1.03 - 1.41	0.018
Primary and nursery teaching professionals	0.0024	0.0013	1.84	1.29 - 2.61	0.00077	0.0019	0.0012	1.56	1.40 - 1.74	7.77×10^{-15}

Table S5 - Covid19 versus mortality from all other causes in men by minor occupational group

Group	Covid19 mortality rate	All other causes mortality rate	Ratio
Administrative Occupations: Finance	45.9	226.4	1:4.9
Administrative Occupations: Government and Related Organisations	58.6	362.2	1:6.2
Administrative Occupations: Records	33.5	155.6	1:4.6
Architects, Town Planners and Surveyors	12.1	127.8	1:10.6
Artistic, Literary and Media Occupations	22.1	248.9	1:11.3
Assemblers and Routine Operatives	32.6	175.5	1:5.4
Building Finishing Trades	42.6	529.6	1:12.4
Business, Finance and Related Associate Professionals	15.4	116.5	1:7.6
Business, Research and Administrative Professionals	13.5	79.9	1:5.9
Caring Personal Services	91	379.3	1:4.2
Construction and Building Trades	37.3	392.5	1:10.5
Construction Operatives	31.6	337.4	1:10.7
Customer Service Occupations	42.9	178.3	1:4.2
Electrical and Electronic Trades	37.3	307.5	1:8.2
Elementary Administration Occupations	54.9	286.8	1:5.2
Elementary Cleaning Occupations	54.3	419.8	1:7.7
Elementary Construction Occupations	82.1	1055.7	1:12.9
Elementary Process Plant Occupations	143.2	856.1	1:6.0
Elementary Security Occupations	93.4	396	1:4.2
Elementary Storage Occupations	54	391.4	1:7.2
Engineering Professionals	10.8	84.7	1:7.8
Functional Managers and Directors	10.3	71.3	1:6.9
Health Professionals	23.9	108.9	1:4.6
Housekeeping and Related Services	29.1	220.4	1:7.6
Information Technology and Telecommunications Professionals	15.6	122.7	1:7.9
Information Technology Technicians	26.8	183.5	1:6.8
Leisure and Travel Services	48.4	257.7	1:5.3
Managers and Directors in Retail and Wholesale	28.3	133.3	1:4.7
Managers and Directors in Transport and Logistics	50	230.3	1:4.6
Managers and Proprietors in Hospitality and Leisure Services	72	421.3	1:5.9
Managers and Proprietors in Other Services	43.5	248.8	1:5.7
Metal Machining, Fitting and Instrument Making Trades	33.2	313.6	1:9.4
Mobile Machine Drivers and Operatives	35.1	363.9	1:10.4
Nursing and Midwifery Professionals	79.1	264.2	1:3.3
Other Administrative Occupations	25.9	185.8	1:7.2
Other Drivers and Transport Operatives	43.7	292.3	1:6.7
Other Elementary Services Occupations	59	360.7	1:6.1
Other Skilled Trades	45.8	329.4	1:7.2
Plant and Machine Operatives	82.3	674.8	1:8.2
Process Operatives	65.7	307.4	1:4.7

For peer review only

Production Managers and Directors	18.1	117.4	1:6.5
Protective Service Occupations	71.2	561.9	1:7.9
Public Services and Other Associate Professionals	17	113.3	1:6.7
Road Transport Drivers	57.8	271.2	1:4.7
Sales Assistants and Retail Cashiers	53.5	312.1	1:5.8
Sales Related Occupations	41.7	215.3	1:5.2
Sales, Marketing and Related Associate Professionals	22.5	175.5	1:7.8
**Secondary education**	**39.2**	**283.8**	**1:7.2**
Teaching and Educational Professionals	18.4	135	1:7.3
Vehicle Trades	60.2	441.3	1:7.3
Welfare Professionals	50.5	206.5	1:4.1

Mortality rates are per 100,000 individuals

For peer review only

Table S6 - Covid19 versus mortality from all other causes in women by minor occupational group

Group	Covid19 mortality rate	All other causes mortality rate	Ratio
Administrative Occupations: Finance	13	146.2	1:11.2
Administrative Occupations: Government and Related Organisations	19.2	198.8	1:10.4
Assemblers and Routine Operatives	39.2	403.8	1:10.3
Caring Personal Services	38.3	258.5	1:6.7
Childcare and Related Personal Services	12.8	120	1:9.4
Customer Service Occupations	13.4	121.6	1:9.1
Elementary Cleaning Occupations	20.6	194.5	1:9.4
Elementary Process Plant Occupations	49.9	401.6	1:8.0
Elementary Security Occupations	17	169.2	1:10.0
Food Preparation and Hospitality Trades	28.6	329.2	1:11.5
Hairdressers and Related Services	39.8	388.8	1:9.8
Managers and Directors in Retail and Wholesale	26.7	224.3	1:8.4
Managers and Proprietors in Hospitality and Leisure Services	35.2	252.8	1:7.2
Managers and Proprietors in Other Services	13.9	155.4	1:11.2
Nursing and Midwifery Professionals	24.7	162.7	1:6.6
Other Administrative Occupations	12	126.5	1:10.5
Other Elementary Services Occupations	21.4	208.5	1:9.7
**Primary and nursery**	**10**	**177.5**	**1:17.8**
Sales Assistants and Retail Cashiers	23.3	209.6	1:9.0
**Secondary education**	**21.2**	**123.8**	**1:5.8**
**School midday and crossing patrol occupations**	**19.2**	**161.9**	**1:8.4**
Secretarial and Related Occupations	10.6	148.1	1:14.0
Teaching and Educational Professionals	9.8	100.2	1:10.2
**Teaching assistants**	**15**	**151.8**	**1:10.1**
Welfare and Housing Associate Professionals	9.2	103.3	1:11.2
Welfare Professionals	22.6	120.9	1:5.3

Mortality rates are per 100,000 individuals

For peer review only

Table S7- Proportionate mortality rates for those working in schools

	Occupation	Covid19 deaths	All deaths	Proportionate mortality rate
Men	Secondary teachers	29	241	0.12 (0.08 to 0.16)
	All other workers	4159	33373	0.12 (0.12 to 0.13)
Women	Primary teachers	19	327	0.06 (0.03 to 0.08)
	Secondary teachers	23	156	0.15 (0.10 t0 0.20)
	Teaching assistants	37	396	0.09 (0.07 to 0.12)
	Midday occupations	18	169	0.11 (0.06 to 0.15)
	All other workers	1669	17623	0.10 (0.09 to 0.10)

For peer review only

Figure S1 – Scatterplot of mortality rate (per 100,000) for Covid19 and all-cause mortality for men, between 9th March and 28th December 2020

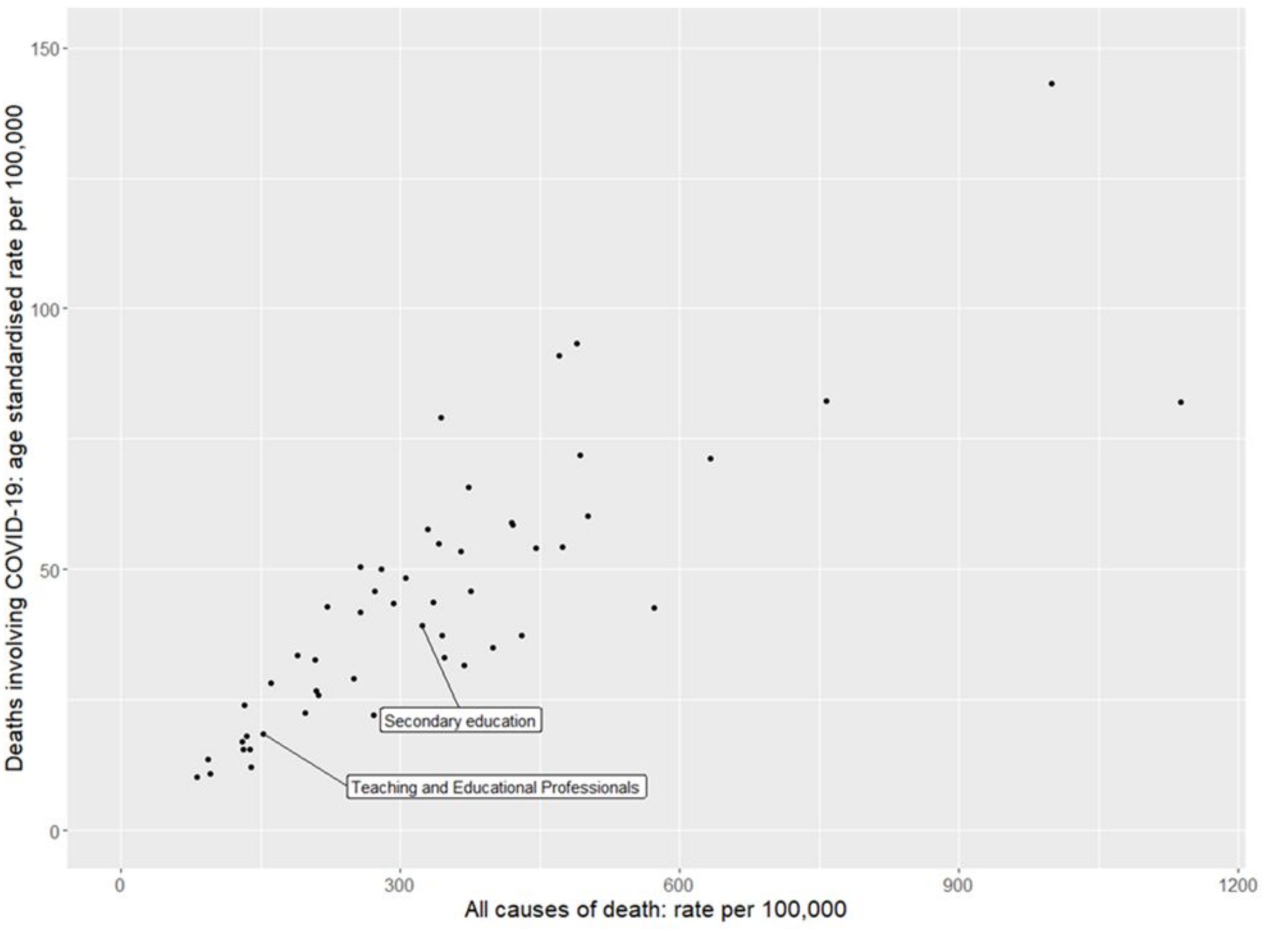

Pearson's correlation coefficient = 0.83, $p = 8.2 \times 10^{-14}$

only

Figure S2 – Scatterplot of mortality rate (per 100,000) for Covid19 and all-cause mortality for women, between 9th March and 28th December 2020

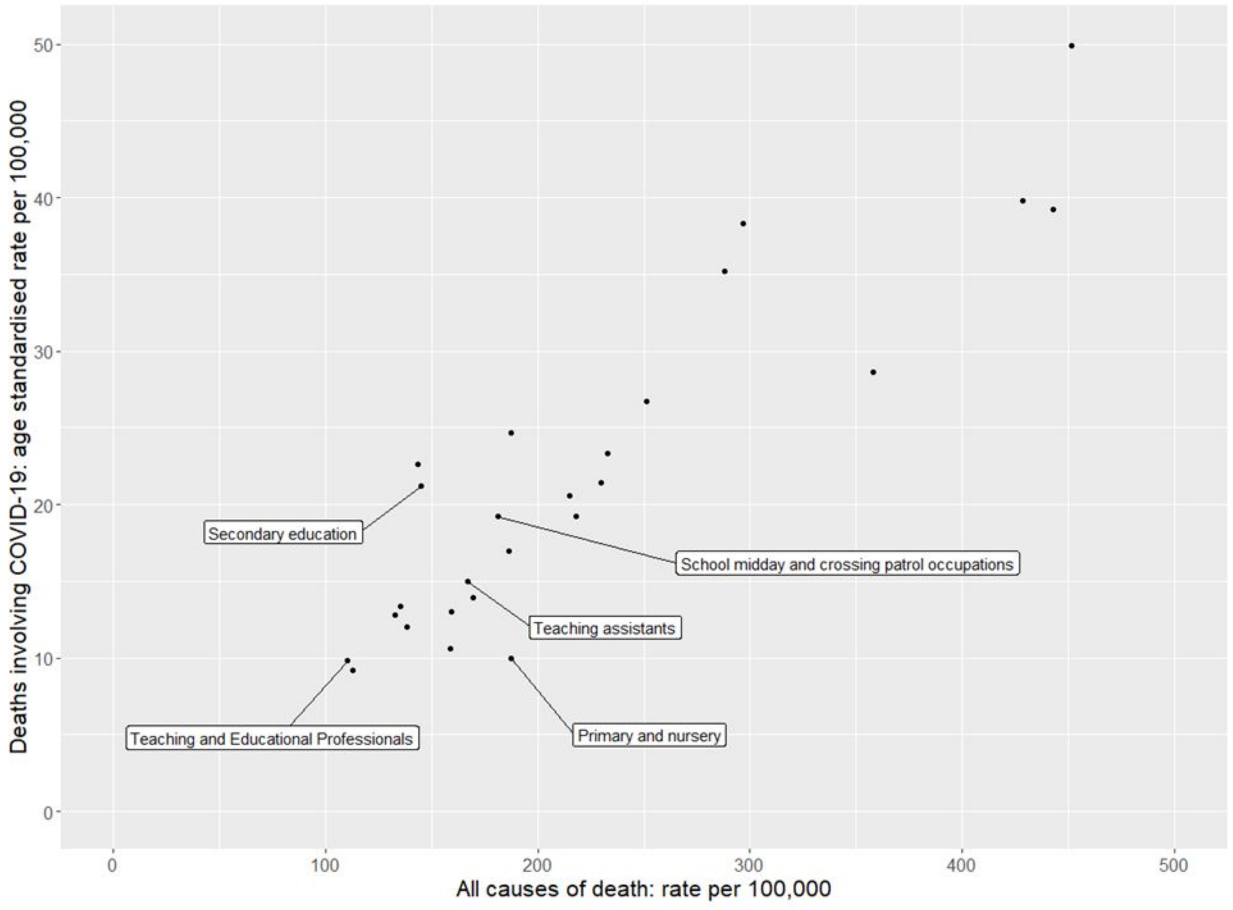

Pearson's correlation coefficient = 0.91, p = 1.4. × 10^{-10}

For peer review only

BMJ Open

Was the risk of death among the population of teachers and other school workers in England and Wales due to Covid19 and all causes higher than other occupations during the pandemic in 2020? An ecological study using routinely collected data on deaths from the Office for National Statistics

Journal:	*BMJ Open*
Manuscript ID	bmjopen-2021-050656.R1
Article Type:	Original research
Date Submitted by the Author:	17-Jul-2021
Complete List of Authors:	Lewis, Sarah; University of Bristol, Population Health Sciences<br>Dack, Kyle; University of Bristol, Population Health Sciences<br>Relton, Caroline; University of Bristol, Population Health Sciences<br>Munafo, Marcus; University of Bristol, School of Psychological Science<br>Davey Smith, George; University of Bristol, MRC- Integrative Epidemiology Unit
**Primary Subject Heading**:	Epidemiology
Secondary Subject Heading:	Occupational and environmental medicine
Keywords:	COVID-19, OCCUPATIONAL & INDUSTRIAL MEDICINE, INFECTIOUS DISEASES

SCHOLARONE™
Manuscripts

I, the Submitting Author has the right to grant and does grant on behalf of all authors of the Work (as defined in the below author licence), an exclusive licence and/or a non-exclusive licence for contributions from authors who are: i) UK Crown employees; ii) where BMJ has agreed a CC-BY licence shall apply, and/or iii) in accordance with the terms applicable for US Federal Government officers or employees acting as part of their official duties; on a worldwide, perpetual, irrevocable, royalty-free basis to BMJ Publishing Group Ltd ("BMJ") its licensees and where the relevant Journal is co-owned by BMJ to the co-owners of the Journal, to publish the Work in this journal and any other BMJ products and to exploit all rights, as set out in our licence.

The Submitting Author accepts and understands that any supply made under these terms is made by BMJ to the Submitting Author unless you are acting as an employee on behalf of your employer or a postgraduate student of an affiliated institution which is paying any applicable article publishing charge ("APC") for Open Access articles. Where the Submitting Author wishes to make the Work available on an Open Access basis (and intends to pay the relevant APC), the terms of reuse of such Open Access shall be governed by a Creative Commons licence – details of these licences and which Creative Commons licence will apply to this Work are set out in our licence referred to above.

Other than as permitted in any relevant BMJ Author's Self Archiving Policies, I confirm this Work has not been accepted for publication elsewhere, is not being considered for publication elsewhere and does not duplicate material already published. I confirm all authors consent to publication of this Work and authorise the granting of this licence.

For peer review only

Was the risk of death among the population of teachers and other school workers in England and Wales due to Covid19 and all causes higher than other occupations during the pandemic in 2020? An ecological study using routinely collected data on deaths from the Office for National Statistics

Sarah J Lewis[1,2], Kyle Dack[1,2], Caroline L Relton[1,2], Marcus R Munafò[1,3] and George Davey Smith[1,2]

1.MRC Integrative Epidemiology Unit at the University of Bristol

2. School of Population Health Sciences, University of Bristol

3. School of Psychological Science, University of Bristol

Corresponding Author: Sarah J Lewis (s.j.lewis@bristol.ac.uk)

Abstract

Objectives: To estimate occupation risk from Covid19 among teachers and others working in schools using publicly available data on mortality in England and Wales.

Design: Analysis of national death registration data from the Office for National Statistics.

Setting: England and Wales, 8th March to 28th December 2020, during the Covid19 pandemic.

Participants: The total working age population in England and Wales plus those still working aged over 65.

Primary and Secondary outcomes: Death with Covid19 as a primary outcome and death from all causes as a secondary outcome.

Results: Across occupational groups there was a strong correlation between Covid19 mortality and both non-Covid19 and all-cause mortality. The absolute mortality rates for deaths with Covid19 were low amongst those working in schools (from 10 per 100,000 in female primary school teachers to 39 per 100,000 male secondary school teachers) relative to many other occupations (range: 10 to 143 per 100,000 in men; 9 to 50 per 100,000 in women).There was weak evidence that secondary school teachers had slightly higher risks of dying with Covid19 compared to the average for all working aged people, but stronger evidence of a higher risk compared to the average for all professionals; primary school teachers had a lower risk. All-cause mortality was also higher amongst all teachers compared to all professionals. Teaching and lunchtime assistants were not at higher risk of death from Covid19 compared with all working aged people.

Conclusion: There was weak evidence that Covid19 mortality risk for secondary school teachers was above expectation, but in general school staff had Covid19 mortality risks which were proportionate to their non-covid mortality risk.

For peer review only

Strengths and limitations of this study

- We used routinely collected data on all deaths in England and Wales; , which are near-complete and not susceptible to serious ascertainment biases.
- We were able to compare mortality data for teachers and other school workers with all other occupational groups and with the working-age population.
- The number of deaths due to Covid19 were small and thus differences between the specific occupational groups were imprecisely estimated.
- We did not have access to individual level mortality data so were not able to account for potential confounders such as comorbidities or household size.
- For those working in school aged over 65 we had neither mortality rates per 100,000 nor total numbers within the group; we only had number of deaths and a 5-year average and we do not know whether the denominators have changed for this group over the last 5 years.

Background

School closures have been implemented in many countries in an effort to slow the spread of the SARS-CoV-2 virus responsible for the Covid19 pandemic. This has affected hundreds of millions of children globally and has been contentious due to harms that have arisen to children as a result of school closures (1). Schools in England and Wales closed to most pupils (except a small number of children of critical workers and some vulnerable children) on the 20th March 2020 and did not reopen fully until September 2020; they closed again for the Christmas holidays (between 11th and 18th December) and reopened on the 8th March 2021 in England. In Wales the reopening of schools after christmas was more gradual and started on the 22nd Feb 2021 with the youngest children, with all children returning by the 15th April 2021.

Whether or not teachers and others working in schools are at higher risk from Covid19 as a result of schools being open is central to decisions on school reopening, but until recently there has not been good data on this. Two as yet unpublished record linkage studies carried out in Scotland (2) and Finland (3) show that teachers have a greater risk of being diagnosed as a Covid19 case compared with the general population. There was a 25% higher risk in the Scottish study, and between 50% and 70% higher risk in the Finnish study. A study in Sweden found that teachers delivering in person teaching had a two-fold higher risk of Covid-19 infection compared to those who were teaching remotely (4). However, studies examining transmission in schools have found this to occur at low levels (5,6). One reason for the discrepancy is that teachers are likely to be under greater surveillance, and therefore undergo more testing than those working in other occupations. In the Scottish study teachers were much more likely to be tested over the autumn term compared with the general population, and this is likely to explain at least some the elevated number of cases among teachers.

Analyses of more robust outcomes such as hospitalisation from Covid19 are less susceptible to detection bias and may be more reliable. The Scottish teachers study (2) found that 47 out of 11,101 teachers were hospitalised with Covid19 up to 26th November 2020, with an odds ratio (OR) of 0.98 (95% CI 0.67 to 1.45) for hospitalisation compared with controls matched on age, sex and general practice registration and adjusted for ethnicity, deprivation, comorbidity counts and sharing a household with a healthcare worker. Sweden has been an outlier in the Covid19 pandemic in that schools have remained open for children up to 16 years of age. A study by the Swedish Public Health Agency found that 20 out of 103,596

For peer review only

school teachers in Sweden received intensive care treatment up to the 30th June 2020, an age and sex matched analysis comparing teachers with other occupations (except health care workers) reported an OR of 0.43 (95% CI 0.28 to 0.68) (7). Similarly, a study carried out in Norway did not find higher rates of hospitalisation with Covid19 for school teachers, after adjustment for age, sex and country of birth (8). There is an absence of evidence on the risk to teaching and lunchtime assistants working in schools.

The Office for National Statistics (ONS) routinely collect mortality data for those living in England and Wales. They released data on deaths with Covid19 and all-cause mortality by occupation as a publicly available dataset (9). We used this data to compared mortality risk among school workers and all working aged adults in England and Wales. We also compared mortality rates among teachers to all professionals, because there are large differences in mortality risk by occupation, largely driven by differences in socioeconomic circumstances (10).

Methods

The ONS mortality statistics used for this analysis are based on mandatory registration of all deaths in England and Wales. So far 3 datasets on mortality with Covid19 by occupation have been released; the first covered the period from 8th March to the 20th April which included the peak of wave 1 deaths, the second covered the period between 8th March to the 25th May and the final dataset (the one we used for the analysis in this paper) covered the period from 8th March to the 28th December.

We used these data to: 1) describe the mortality rates among all educational professionals combined and present these alongside rates for all professionals and all working aged people for the different time periods covered by this data (Table 1) , 2) compare the number of deaths with historical data on deaths for the same occupational group over 5 years (2015 to 2019) for all school workers and for individual occupations (teachers, teaching assistants, school secretaries etc), which are presented alongside death rates among all occupations and all professional occupations over the same period of the pandemic (Table 2 and S1), 3) calculate the risk of Covid19 and all cause mortality among teachers and other school staff compared with all working aged people (Table 3 and 4) and all professionals (Supplementary Table S2 and S3) and finally, 4) we investigated the ratio of mortality with Covid19 to mortality from other causes among different occupational groups to determine whether school staff were outliers (Supplementary Table S4-S6 and all Figures).

Occupational exposure groups

Occupation was reported on the death certificate at the time of death registration by the informant and was coded according to the ONS standard occupational classification 2010 (11). Population counts for occupational groups were estimated from the Annual Population Survey (APS), conducted in 2019 (12). The APS is the largest ongoing household survey in the UK, conducting interviews with members of randomly selected households.

The group ‘teaching and educational professionals’, as defined by the ONS, includes; higher education teaching professionals, further education teaching professionals, secondary education teaching professionals, primary and nursery education teaching professionals, special needs education teaching professionals, senior professionals of educational establishments, education advisers and school inspectors and teaching and other educational professionals not elsewhere defined.

The group ‘teaching and educational professionals’ does not include: teaching assistants, school secretaries, lunchtime assistants or educational support assistants, but these

For peer review only

occupations were considered along with teachers working in schools where sufficient data were available.

The group defined by ONS as midday and crossing patrol occupations, includes some individuals who work as road crossing patrols, however not all schools have these and if they do usually only have one, so the group will mainly comprise of those who supervise children in school during their lunch break and who prepare school lunch. Therefore midday and crossing patrol occupations will be shorted and referred to in this manuscript as lunchtime assistants.

The comparison groups we used were:

1) All working aged people – for comparisons of mortality rates and risk presented in Tables 1, 3 and 4.
2) All occupations – for comparisons of number of deaths presented in Tables 2 and S1.
3) All professional occupations – for comparisons of mortality risk presented in Tables S2 and S3.

Professional occupations are those which are classified by ONS as major occupational group 2, these are occupations which require a degree or equivalent period of relevant work experience and include, but are not limited to; scientists, engineers, architects, doctors, nurses, radiographers, physiotherapists, social workers and solicitors, for further information see reference 11.

Outcome definition

ONS define deaths with Covid19 as those where Covid19 or suspected Covid19 was mentioned anywhere on the death certificate, including in combination with other health conditions. If a death certificate mentions Covid19, it will not always be the main cause of death but may be a contributory factor.

Statistical analysis

We calculated the relative risk of death for education professions compared to all working aged (20 to 64 year olds) people and compared teachers to all professionals in England and Wales stratified by sex, for this we used published ONS data on total deaths and age adjusted mortality rates per 100,000 for each group. We derived the denominator (total population) for each group of interest as

Risk =mortality rate per 100,000 taken from ONS data/100,000.

We calculated 95% confidence intervals and *p*-values using the formula;

$$CI = \exp(\ln(RR) \pm 1.96 \times \sqrt{(1/deaths + 1/control\ deaths - 1/population - 1/control\ population)}$$

$$P = \exp((-0.717 \times (RR/SE)) - (0.416 \times (RR/SE)^2))$$

The ONS data included deaths for 8 subgroups of educational professionals, we included those with a sufficient number of Covid19 deaths (≥10) in our analysis. In addition, sufficient data were available to include female teaching and lunchtime assistants in this analysis. We also performed a fixed effect meta-analysis across the 4 occupational groups working in schools among females.

For peer review only

We estimated mortality rates for all other causes (other than Covid19) by calculating the difference between all-cause mortality and mortality with Covid19 for each minor occupational group as defined by ONS (9, 11).

We produced scatterplots of 'all cause' versus 'with Covid19' mortality and 'all other causes' versus 'with Covid19' mortality by occupational group for men and women separately and calculated Pearson's correlation coefficient for these plots.

We also calculated proportionate mortality ratios as mortality rates from Covid19 divided by all-cause mortality for occupations working in schools.

Finally, we used our estimates from above for the SE and the denominators for each occupational group to estimate the variance for Covid19 mortality. We then performed a weighted least squares regression analysis (weighted by 1/variance) of Covid19 mortality against other cause to determine the increase in Covid19 for 1 death increase in other cause mortality across occupational groups.

Patient and Public Involvement statement

The public were not involved in the design or conduct of this study.

Ethical approval

Ethical approval was not sought for this project because we used publicly accessible summary statistics of a national mandatory data collection.

Results

Death rates from Covid19 and all causes among all education professionals presented alongside death rates for all professionals and all working aged people by time period.

Between the 8th March 2020 and 28th December 2020 there were 68,757 deaths from all causes among the working age (20-64 years) population in England and Wales, 7,961 (12%) of these deaths involved Covid19, of which, 2,494 had occurred prior to the 20th April, 2,267 occurred between 20th April and the 25th May, and 3,200 occurred between the 25th May and the 28th December 2020.

There were 1,326 deaths from all causes among educational professionals during this period; 139 of those deaths were thought to involve Covid19.

Table 1

Covid19 was involved in 1 in 8 male deaths and 1 in 9 female deaths among working aged people; among education professionals these figures were approximately 1 in 8 deaths among males and 1 in 11 among females. Covid19 was involved in a higher proportion of deaths early on in the pandemic; data covering the 6 weeks of the first peak in the UK showed that Covid19 was involved in 1 in 4 deaths in males and 1 in 6 deaths in female education professionals during this time. During the 10 months covered by the ONS data all-cause and Covid19 mortality rates among educational professionals appeared to be lower than for all working aged adults and similar to those for all professionals.

Number of deaths by individual occupations and combined across all school workers compared with 5-year average rates

We examined number of deaths by occupation among those working in schools aged 20-64 years (Table 2) and among those aged 65 or older (Supplementary table S1). Among

For peer review only

working aged people when deaths across all occupations working in schools were combined, we observed 15% more deaths (excess) compared with the 2015-2019 average for the same period among men and 5% more among women. For women this was less than the excess observed across all occupations and among all professionals; for men it was similar. Deaths with Covid19 appeared to account for almost all the excess among men whereas among women there were twice as many deaths with Covid19 among school workers as there were excess deaths.

Among the older age group there were much higher numbers of deaths compared with their 5-year average (74% more deaths amongst males and 37% among females). However, only 33% of excess deaths in men and 37% in women were thought to involve Covid19. The group of all professionals and of all occupations had fewer excess deaths as a proportion of their 5-year average but a higher proportion of the deaths involved Covid19 compared with those working in schools.

Comparison of Covid19 and all-cause deaths among education professionals and those working in schools compared to the working aged population

Table 3 shows that compared to the total working aged population there was strong evidence that the risk of dying with Covid19 was lower among all education professionals combined (RR_{Males} 0.59, 95%CI 0.46 to 0.75, $RR_{females}$ 0.58, 95%CI 0.46 to 0.73). There was also some evidence of a lower risk of death with Covid19 among female primary and nursery education professionals (RR 0.60, 95%CI 0.38 to 0.93). For both male and female secondary school teachers the relative risk estimate shows a 25% higher risk of death from Covid19, although there was a great deal of uncertainty around these estimates (RR_{Males} 1.25, 95%CI 0.87 to 1.80, $RR_{females}$ 1.26, 95%CI 0.84 to 1.90). There was little evidence of a difference in risk between teaching assistants, or lunchtime assistants compared with working aged people. A fixed effect meta-analysis across the 4 occupational groups working in schools in women showed that risk was similar to all working aged people (RR 0.95, 95CI 0.77 to 1.14), with weak evidence of heterogeneity across the groups (I^2=55.2%, Chi^2=6.697, p=0.08).

Table 4 shows strong evidence that the risk of death from all causes during this pandemic period was lower for higher and further education teacher professionals compared with the working aged population of England and Wales. The was also evidence that male secondary school teachers (RR 1.26, 95%CI 1.11 to 1.43) and female primary school teachers (RR 1.18, 95%CI 1.06 to 1.32) had slightly higher mortality risk compared to the general population.

We also calculated relative risks for mortality with Covid19 and all-cause mortality in educational professionals versus all professionals (Supplementary Tables S2 and S3). We found strong evidence that both male and female secondary school teachers had a higher risk of mortality with Covid19 compared with all professionals. In addition male and female primary and secondary school teachers had higher mortality risks for all causes compared with all professionals.

Comparison of Covid19 to other cause mortality across occupational groups

Figures 1 and 2 show that there are strong correlations between all other cause mortality and mortality with Covid19. Weighted least squares regression analysis showed that for every death from other causes there were 0.11 (95%CI=0.09 to 0.13, $p=2.0\times10^{-16}$) deaths from Covid19, with no meaningful difference between men (0.11, 95%CI 0.09 to 0.13, $p=2.49\times10^{-12}$) and women (0.10, 95%CI 0.08 to 0.13, $p=4.41\times10^{-9}$).

For peer review only

We also plotted mortality with Covid19 versus all-cause mortality on separate scatterplots (Figures S1 and S2) and found similar results.

Ratio of mortality with Covid19 to other causes

The ratio of deaths with Covid19 to other causes of death, ranged from 1:3 among male nurses and midwifes to 1:13 among male elementary construction occupations (Supplementary Table S4). The ratio of Covid19 to other causes of death for male secondary school teachers was 1:7, which was the same as the average for all male professionals and all working aged men. Among women (Supplementary Table S5), the ratio of mortality with Covid19 to other causes of death, ranged from 1:5 among welfare professionals (social workers and probation officers) to 1:18 among primary and nursery schoolteachers; ratios for female secondary school teachers, teaching assistants and lunchtime assistants were 1:6, 1:10 and 1:8 respectively. Proportionate mortality rates (Covid19 deaths divided by deaths from all causes) are presented in Supplementary Table S6.

Discussion

Covid19 deaths among education professionals and others working in schools

We used routinely collected data on mortality for the Office of National Statistics in the UK to study deaths with Covid19 among teachers and other school staff, during the pandemic in 2020. We found that all educational professionals combined had fewer deaths per 100,000 occurring with Covid19 compared to the general population. This is to be expected since the total working aged population of England and Wales will include those who are unable to work due to ill health. The group ‘education professionals’ includes those working in universities and school inspectors, many of whom have been working from home throughout the pandemic. We therefore investigated excess deaths specifically among school teachers and other staff working in schools and compared them to all those currently working and all professionals. There were fewer deaths than the 5-year average among female primary and secondary school teachers and the number of deaths among male teachers were similar to the 5-year average. There were more deaths among teaching assistants compared to the 5-year average, but only around half of the excess deaths were thought to involve Covid19. For all women working in schools combined there was a small increase in the number of deaths (5%) compared with the 5-year average, but the number of deaths occurring with Covid19 was greater than the excess, suggesting that some of those dying with Covid19 may have died over the course of a normal year from other causes. For those working in schools who were aged 65 and over there were large excesses in deaths compared with the average for the previous 5 years (74% for men and 37% for women), but only around a third of the excess deaths were thought to involve Covid19. It is possible that some excess deaths, were due to undiagnosed Covid19 among those who did not have classic symptoms (13), but this is less likely for later deaths as the amount of testing for Covid19 increased dramatically over time in the UK. We do not have information on other causes of death within this population over this time period, however in 2019 the most common causes of death among over 65s in England and Wales were heart disease and cancer (14). It is possible that more school staff were dying from other causes during this period due to delayed treatments for other conditions or due to an unwillingness to seek help for fear of contracting Covid19 or of overburdening the healthcare system. It is also possible the number of school staff in this group has increased over the last 5 years. Further research is needed to determine whether these represent numerator-denominator bias or if there is a true excesses and if so the cause of these excess deaths.

For peer review only

Relative risk of death with Covid19 among school staff

We found that secondary school teachers had around a 25% higher risk (in both men and women) of dying with Covid19 compared with the general population, although the evidence was weak. There was stronger evidence of an increased risk to secondary school teachers compared to all professionals. However, all-cause mortality was also increased among secondary school teachers compared to all professionals.

Proportionate mortality ratios (mortality from a specific cause: 'all cause' or 'all other cause mortality') are routinely used in occupational epidemiology in situations where calculation and adequate standardization of mortality rate ratios is not possible (15). The rationale for doing this is that if an occupation has a particular risk for a specific disease the proportion of deaths due to that disease will be increased relative to deaths from other causes in the group. We therefore plotted the correlation between age adjusted mortality for all causes and mortality with Covid19 across minor occupational groups for whom risk was reported in the ONS dataset, the two measures are bound to be correlated to a certain extent because all-cause mortality includes with Covid19. However, we also found a strong positive correlation between mortality with Covid19 and non-Covid19. This is consistent with findings from others which show that deaths from Covid19 closely match 'normal' risk within the UK population (13,16). Mortality from any cause amongst all education professionals and teachers were towards the lower end of the distribution. For male secondary teachers, female teaching and lunchtime assistants the ratio of mortality with Covid19 to all-cause mortality was consistent with other occupational groups; female primary school teachers and female secondary school teachers appeared to be outliers in opposite directions, however the number of deaths in these groups was small and a meta-analysis across occupations working in school showed only weak evidence of heterogeneity.

Consistency with other studies

The data we examined from ONS is consistent with the Public Health Scotland study (2) and with the Swedish study (7) which both showed similar numbers of hospitalisations due to Covid19 among teachers compared with other occupations, but our findings are also compatible with a slightly higher risk of Covid19 mortality among secondary school teachers. Mutambudzi and colleagues (17) investigated the risk of hospitalisation or death by occupation after testing positive for Covid19 among 120,075 working participants (aged 49 to 64 years) in the UK Biobank study, they found weak evidence of more hospitalisations or deaths with Covid19 among education workers compared to non-essential workers up to July 2020 (OR 1.59, 95%CI 0.87 to 2.91) after adjusting for potential confounders. However, UK Biobank is a highly selected population, which is not representative of the UK population due to a response rate of just 5% (18).

A study of data on teacher absence due to Covid19 in England during the Autumn term (September-December) 2020 found that the proportion of teachers absent due to infection appeared to be similar in both primary and secondary schools (19).The ONS schools infection survey found that primary and secondary staff had similar antibody positivity rates for SARS-CoV2 (15% and 16% respectively) at the end of Autumn term, which reflected exposure during the time when in person teaching was taking place; positivity rates among all teachers were slightly lower than among all working aged adults (18%) (20). Despite this, primary school teachers in England and Wales had a lower risk of death with Covid19 compared to secondary school teachers (although confidence intervals were overlapping).

Very few studies have examined risk among teaching assistants and lunchtime assistants as separate groups; we found that despite more deaths occurring in this group in 2020

For peer review only

compared to the previous 5 years, they were not at increased risk of death due to Covid19 or any cause compared to working aged people. A study carried out in California used modelling to compare deaths from all causes during the pandemic and found an odds ratio for teaching assistants of 1.28, but no confidence intervals were provided for that study (21).

Strengths and limitations of the study

We used routinely collected data on mortality which includes all deaths in England and Wales, and results are therefore unlikely to be due to ascertainment bias and will be representative of the working aged population of these countries. However, we did not have access to individual level mortality data so were not able to account for potential confounders such as comorbidities or household size. For our relative risk calculations our comparison group also included school staff (although they only made up a very small proportion of the total) because we did not have age adjusted mortality rates excluding teachers. The age adjusted mortality rates calculated by ONS were based on denominators take from a survey conducted in 2019, if the school workforce has changed as a result of the pandemic these rates will be inaccurate, if any changes in the school workforce are systematically different to other professions the results will be biased. Whilst the size of the school workforce in England remained fairly stable between 2015-2019, there was a marked decrease in the number of teachers retiring (from 17,853 teachers retiring in 2015 to 12,062 teachers in 2019) (22), suggesting that the average age of teachers may have increased over this time period. We don't know what impact the pandemic has had on the size and age distribution of those working in schools, it could be that older more vulnerable staff have retired or conversely staff may have stayed on to help with staff shortages due to teachers having to isolate. Therefore, we were unable to adequately assess the extent to which risk of death had changed during the pandemic period for these occupational groups especially among those aged over 65. In addition, the number of deaths were extremely small and subject to random fluctuations in some groups. Whilst our results are likely to be generalisable to countries which are similar to the UK, they may not be generalisable to developing countries and countries with different social structures.

In the period covered by this data many school staff worked remotely for large periods of time during 2020, a survey carried out by the global education company Tes found that just 22% of teachers were engaged in face-to-face teaching during the first lockdown (23),The more vulnerable staff are likely to have worked from home even when schools were fully open, which will have an impact on their workplace exposure. However, all teachers were potentially exposed to Covid19 up until schools closed on 20th March 2020 and deaths up to 25th May, just two months after schools were closed, accounted for approximately two thirds of all deaths occurring over the study period (Table 1). Given the lag between infection and death, many people dying up to the 25th May could have been infected prior to school closures. We do not have data on deaths by date and occupation, however, out of a total of 68,186 deaths which occurred due to Covid19 in England and Wales between the dates covered by this data (24), only 5018 deaths (7.4%) occurred between 25th May 2020 and the 30th September 2020, a period during which those dying were unlikely to have been infected in school due to school closures. Therefore the vast majority of people dying of Covid19 during the period covered by the study could have been infected when schools were open.

In Conclusion

Teachers, teaching and lunchtime assistants aged 20-64 years were not at high risk of death relative to the working age population in England and Wales during the Covid19 pandemic. For occupations working in schools Covid19 mortality was generally proportionate to all-cause mortality. Female primary school teachers and female secondary school teachers

For peer review only - http://bmjopen.bmj.com/site/about/guidelines.xhtml

seemed to be outliers in opposite directions however, evidence for heterogeneity in Covid19 mortality risk among school workers was weak. For policy makers who are considering the impact of schools being open in future pandemics it will be important to note that staff were not at increased risk.

Contributorship statement – SL and GDS conceived the idea for the manuscript and designed the analyses, SL and KD performed the analyses, SL, GDS, CR and MM all contributed to the interpretation of the results, all authors contributed to drafting the manuscript and have seen and approved the final version

Competing interests – The authors have no competing interests.

Funding- Sarah Lewis, Caroline Relton and George Davey Smith have received a grant from National Institute for Health Research (NIHR) and UK Research and Innovation (UKRI)COVID-19 mapping and mitigation in schools (CoMMinS)

Data sharing statement-The data used in this manuscript have been made publicly available by the Office of National Statistics (ONS) in the United Kingdom

For peer review only

References

1. Buonsenso, D.; Roland, D.; De Rose, C.; Vásquez-Hoyos, P.; Ramly, B.; Nandipa Chakakala-Chaziya, J.; Munro, A.; González-Dambrauskas, S. Schools Closures during the COVID-19 Pandemic: A Catastrophic Global Situation. *Preprints* 2020, 2020120199 (doi: 10.20944/preprints202012.0199.v1).

2. Fenton L, Gribben C, Caldwell D, Colville S, Bishop J, Reid M, White J, Hutchinson S, Robertson C, Colhoun HM, Wood R, McKeigue PM, McAllister DA. Risk of hospitalisation with COVID-19 among teachers compared to healthcare workers and other working-age adults. A nationwide case-control study. MedRxiv 2021.02.05.21251189 [Preprint]; doi: https://doi.org/10.1101/2021.02.05.21251189

3. Helsinki Gradate School of Economics. Situation Room Report: The Corona Virus and Health Differences – In Which Socioeconomic Groups Have the Most Infections Been Observed in Finland? 19th January, 2021. https://www.helsinkigse.fi/covid19-data-en/situation-room-report-the-corona-virus-and-health-differences-in-which-socioeconomic-groups-have-the-most-infections-been-observed-in-finland/

4. Vlachos J, Hertegård E, Svalery HB. The effects of school closures on SARS-CoV-2 among parents and teachers. Proceedings of the National Academy of Sciences Mar 2021, 118 (9) e2020834118; DOI: 10.1073/pnas.2020834118

5. Ismail SA, Saliba V, Lopez Bernal J, Ramsay ME, Ladhani SN. SARS-CoV-2 infection and transmission in educational settings: a prospective, cross-sectional analysis of infection clusters and outbreaks in England. Lancet Infect Dis. 2020 Dec 8:S1473-3099(20)30882-3. doi: 10.1016/S1473-3099(20)30882-3. Epub ahead of print.

6. Falk A, Benda A, Falk P, Steffen S, Wallace Z, Høeg TB. COVID-19 Cases and Transmission in 17 K–12 Schools — Wood County, Wisconsin, August 31–November 29, 2020. MMWR Morb Mortal Wkly Rep 2021;70:136–140. DOI: http://dx.doi.org/10.15585/mmwr.mm7004e3

7. Ludvigsson JF, Engerström L, Nordenhäll C, Larsson E. Open Schools, Covid-19, and Child and Teacher Morbidity in Sweden. N Engl J Med. 2021 Jan 6:NEJMc2026670. doi: 10.1056/NEJMc2026670. Epub ahead of print. https://www.nejm.org/doi/full/10.1056/NEJMc2026670

8. Magnusson K, Nygård K, Vold L, Telle K. Occupational risk of COVID-19 in the 1st vs 2nd wave of infection. MedRxiv *Preprints* https://www.medrxiv.org/content/10.1101/2020.10.29.20220426v1.full.pdf

9. Coronavirus (COVID-19) related deaths by occupation, England and Wales. Release date 25th January 2021. https://www.ons.gov.uk/peoplepopulationandcommunity/healthandsocialcare/causesofdeath/datasets/coronaviruscovid19relateddeathsbyoccupationenglandandwales

10. Katikireddi SV, Leyland AH, McKee M, Ralston K, Stuckler D. Patterns of mortality by occupation in the UK, 1991-2011: a comparative analysis of linked census and mortality records. Lancet Public Health. 2017 Oct 23;2(11):e501-e512. doi: 10.1016/S2468-2667(17)30193-7.

11. Office for National Statistics. SOC2010 volume 1: structure and descriptions of unit groups.

https://www.ons.gov.uk/methodology/classificationsandstandards/standardoccupationalclassificationsoc/soc2010/soc2010volume1structureanddescriptionsofunitgroups

12. Office for National Statistics. Annual population survey (APS) QMI. https://www.ons.gov.uk/employmentandlabourmarket/peopleinwork/employmentandemployeetypes/methodologies/annualpopulationsurveyapsqmi

13. Spigelhalter D. Use of "normal" risk to improve understanding of dangers of covid-19. BMJ 2020; 370: m3259. https://doi.org/10.1136/bmj.m3259

14. Office for National Statistics. Deaths registered in England and Wales. Release date July 2020. https://www.ons.gov.uk/peoplepopulationandcommunity/birthsdeathsandmarriages/deaths/datasets/deathsregisteredinenglandandwalesseriesdrreferencetables

15. Decouflé P, Thomas TL, Pickle LW. Comparison of the proportionate mortality ratio and standardized mortality ratio risk measures. Am J Epidemiol. 1980 Mar;111(3):263-9. doi: 10.1093/oxfordjournals.aje.a112895.

16. Bhaskaran K, Bacon SCJ, Evans SJW, Bates CJ, Rentsch CT, MacKenna B, Tomlinson L, Walker AJ, Schultze A, Morton CE, Grint D, Mehrkar A, Eggo RM, Inglesby P, Douglas IJ, McDonald HI, Cockburn J, Williamson EJ, Evans D, Curtis HJ, Hulme WJ, Parry J, Hester F, Harper S, Spiegelhalter D, Smeeth L, Goldacre B. Factors associated with deaths due to COVID-19 versus other causes: population-based cohort analysis of UK primary care data and linked national death registrations within the OpenSAFELY platform. MedRxiv *Preprints* https://www.medrxiv.org/content/10.1101/2021.01.15.21249756v2.full.pdf

17. Mutambudzi M, Niedwiedz C, Macdonald EB, et al. Occupation and risk of severe COVID-19: prospective cohort study of 120 075 UK Biobank participants. Occupational and Environmental Medicine Published Online First: 09 December 2020. doi: 10.1136/oemed-2020-106731. https://oem.bmj.com/content/early/2020/12/01/oemed-2020-106731.full#DC1

18. Tyrrell J, Zheng J, Beaumont, R. *et al.* Genetic predictors of participation in optional components of UK Biobank. *Nat Commun* **12,** 886 (2021). https://doi.org/10.1038/s41467-021-21073-y

19. Southall E, Holmes A, Hill EM, Atkins BD, Leng T, Thompson RN, Dyson L, Keeling MJ, Tildesley MJ. An analysis of school absences in England during the Covid-19 pandemic. medRxiv 2021.02.10.21251484 [Preprint]; doi: https://doi.org/10.1101/2021.02.10.21251484

20. Office for National Statistics. COVID-19 Schools Infection Survey Round 2, England: December 2020. https://www.ons.gov.uk/peoplepopulationandcommunity/healthandsocialcare/conditionsanddiseases/bulletins/covid19schoolsinfectionsurveyround2england/december2020#main-points

21. Chen Y-H, Glymour M, Riley A, Balmes J, Duchowny K, Harrison R, Matthay E, Bibbins-Domingo K. Excess mortality associated with the COVID-19 pandemic among Californians 18–65 years of age, by occupational sector and occupation: March through October 2020. medRxiv 2021.01.21.21250266 [Preprint]; doi: https://doi.org/10.1101/2021.01.21.21250266

22. United Kingdom Government. Reporting Year 2019. School workforce I n England. https://explore-education-statistics.service.gov.uk/find-statistics/school-workforce-in-england#dataBlock-4189d4c5-ba35-4f48-9248-cc39f53703a2-tables

23. Tes. Reality of lockdown for school staff: 5 key findings. https://www.tes.com/news/reality-lockdown-school-staff-5-key-findings

24. Gov.uk. Coronavirus (COVID-19) in the UK. https://coronavirus.data.gov.uk/

For peer review only

Table legends

Table 1 Age adjusted all-cause and with Covid19 mortality per 100,000 population (95% confidence intervals) taken from 3 ONS datasets on Covid19 mortality by occupation covering different time periods during the 2020 Covid19 pandemic. Incudes the ratio of Covid19 deaths to all-cause deaths for education professionals, all professionals and all working aged people (aged 20-64 years) stratified by sex.

Table 2- Deaths among working aged (20-64) people who working in education in England and Wales 9th March to 28th December 2020. This table shows the number of deaths by occupation in 2020 and the average for the previous 5 years among those working in schools aged 20-64 years, also includes figures for all occupations and all professionals for comparison. The number of excess deaths was calculated by subtracting the 5 year average number of deaths (provided by ONS) from the number of deaths from all causes between 9th March and 28th December 2020 for each occupation and occupational group. The number of excess deaths was converted into a percentage of the 5-year average (percentage excess). The percentage excess explained by Covid19 was calculated by dividing the number of Covid19 deaths by the number of excess deaths and multiplying this by 100. We set the maximum percentage excess deaths to be 100, but we did not cap the percentage excess explained by Covid19. The reason for this is to determine whether Covid19 could be a factor in some deaths which may have occurred anyway.

Table 3 Relative risks for death occurring with Covid19 for school staff aged 20-64 years compared to the total working aged population, calculated using age-adjusted mortality rates by occupation between 9th March and 28th December 2020 from the ONS dataset.

Table 4 Relative risks for death occurring from all causes for school staff aged 20-64 years compared to the total working aged population, calculated using age-adjusted mortality rates by occupation between 9th March and 28th December 2020 from the ONS dataset.

Figure legends

Figure 1 Scatterplot of mortality rate (per 100,000) for Covid19 and all other causes of death for men, using age adjusted mortality rates between 9th March and 28th December 2020 from ONS. Unlabelled data points represent occupational groups in Supplementary Table S4. Pearson's correlation coefficient = 0.78, $p = 1.4 \times 10^{-11}$

Figure 2 Scatterplot of mortality rate (per 100,000) for Covid19 and all other causes of death for women, using age adjusted mortality rates between 9th March and 28th December 2020 from ONS. Unlabelled data points represent occupational groups in Supplementary Table S5. Pearson's correlation coefficient = 0.88, $p = 1.7. \times 10^{-09}$

For peer review only

For peer review only

Table 1- Age adjusted all cause and with Covid19 mortality rates per 100,000 population (95% confidence intervals) for all educational professionals, all professional occupations and all working aged adults in England and Wales by overlapping time periods covered by this data.

Occupational group	Cause of Mortality	Males			Females		
		8th March-20th April	8th March-25th May	8th March-28th December	8th March-20th April	8th March-25th May	8th March-28th December
Working aged adults	With Covid19	9.9 (9.4-10.4)	19.1 (18.4-19.8)	31.4 (30.6-32.3)	5.2 (4.9-5.6)	9.7 (9.3-10.2)	16.8 (16.2-17.5)
	All cause	41.6 (40.6-42.6)	78.1 (76.7-79.4)	256.0 (253.5-258.4)	26.3 (25.5-27.1)	48.4 (47.3-49.4)	158.3 (156.4-160.2)
	Ratio*	1:4.2	1:4.1	1:8.2	1:5.1	1:5.0	1:9.4
Professional Occupations	With Covid19	5.6 (4.6-6.6)	11.6 (10.2-13.0)	17.6 (15.9 - 19.3)	4.2 (3.3-5.2)	8.0 (6.8 to 9.3)	12.8 (11.2- 14.4)
	All cause	22.2 (20.2-24.1)	41.2 (38.5-43.8)	130.4 (125.7-135.2)	22.0 (19.9-24.1)	39.3 (36.5-42.1)	120.0 (115.2-124.9)
	Ratio*	1:4	1:3.6	1:7.4	1:5.2	1:4.9	1:9.4
Educational professionals	With Covid19	6.7 (4.1-10.3)	12.9 (9.3-17.4)	18.4 (14.0-23.6)	3.3 (2.0-4.9)	6.0 (4.2-8.1)	9.8 (7.5-12.5)
	All cause	28.0 (22.4-34.5)	48.1 (40.4-55.7)	153.4 (139.8-167.1	20.4 (16.9-24.0)	37.2 (32.5-42.0)	110.0 (101.9-118.2)
	Ratio*	1:4.2	1:3.7	1:8.3	1:6.2	1:6.2	1:11.2

*Ratio of deaths involving Covid19 deaths to all deaths

For peer review only

Table 2 Number of deaths among working aged (20-64) people who were working in education in England and Wales 9th March to 28th December 2020

Description	Men						Women					
	Deaths involving COVID-19	All causes of death	All causes 5 year average	Excess	% excess	Excess due to Covid19	Deaths involving COVID-19	All causes of death	All causes 5 year average	Excess	% excess	Excess due to Covid19
Secondary education teaching professionals	29	241	233	8	3%	100%	23	156	173	-17	-10%	NA
Primary and nursery education teaching professionals	4	31	30	1	3%	100%	19	327	368	-41	-11%	NA
Special needs education teaching professionals	1	12	8	4	50%	25%	3	27	33	-6	-18%	NA
Teaching and other educational professionals n.e.c.	8	56	32	14	44%	57%	7	70	61	9	15%	78%
School secretaries	0	3	2	1	50%	0%	4	58	53	5	9%	80%
Teaching assistants	5	28	19	9	47%	56%	37	396	307	89	29%	42%

For peer review only

**Educational support assistants**	1	7	8	-1	-13%	NA	3	55	58	-3	-5%	NA
**School midday and crossing patrol occupations**	2	11	5	6	120%	33%	18	169	149	20	13%	90%
**All those working in schools**	50	389	337	52	15%	96%	114	1258	1202	56	5%	204%
**All professional occupations**	419	3144	2765	379	14%	111%	279	2696	2477	219	9%	127%
**All occupations**	4225	33904	29745	4159	14%	102%	1742	18419	16528	1891	11%	92%

For peer review only

Table 3 Covid19 deaths for educational professions aged 20-64 years compared to all working aged adults, between 9th March and 28th December 2020

**Description**	**Men**					**Women**				
	Risk*	Risk for all workers	RR	95% CI	P-value	Risk*	Risk for all workers	RR	95% CI	P-value
All educational professionals	0.00018	0.00031	0.59	0.46 - 0.75	0.000018	0.000098	0.00017	0.58	0.46 - 0.73	0.0000058
Higher education teaching professionals	0.00012	0.00031	0.37	0.20 - 0.68	0.0016	NA[1]				
Further education teaching professionals	0.00025	0.00031	0.79	0.42 - 1.46	0.46	NA[1]				
Secondary education teaching professionals	0.00039	0.00031	1.25	0.87 - 1.80	0.23	0.00021	0.00017	1.26	0.84 - 1.90	0.27
Primary and nursery teaching professionals	NA[1]					0.00010	0.00017	0.60	0.38 - 0.93	0.024

Teaching assistants	NA[1]					0.00015	0.00017	0.89	0.65 - 1.23	0.5
Lunchtime assistants	NA[1]					0.00019	0.00017	1.14	0.72 - 1.82	0.58
[*1]Less than 10 deaths, rate not reported.										

For peer review only

Table 4 All-cause mortality for school staff aged 20-64 years compared to all working aged adults, between 9th March and 28th December 2020

Description	Men					Women				
	Risk*	Risk for all workers	RR	95% CI	P-value	Risk*	Risk for all workers	RR	95% CI	P-value
All educational professionals	0.0015	0.0026	0.60	0.55 - 0.65	1.74×10^{-29}	0.0011	0.0016	0.69	0.65 - 0.74	6.14×10^{-23}
Higher education teaching professionals	0.00095	0.0026	0.37	0.30 - 0.47	2.00×10^{-16}	0.00073	0.0016	0.46	0.35 - 0.61	1.13×10^{-07}
Further education teaching professionals	0.0017	0.0026	0.66	0.52 - 0.84	0.00077	0.00096	0.0016	0.61	0.47 - 0.79	0.00015
Secondary education teaching professionals	0.0032	0.0026	1.26	1.11 - 1.43	0.00033	0.0015	0.0016	0.92	0.78 - 1.07	0.28
Primary and nursery teaching professionals	0.0024	0.0026	0.94	0.66 - 1.33	0.72	0.0019	0.0016	1.18	1.06 - 1.32	0.0023
Teaching assistants	0.0017	0.0026	0.65	0.45 - 0.95	0.024	0.0017	0.0016	1.05	0.95 - 1.16	0.3
Lunchtime assistants	NA[1]					0.0018	0.0016	1.14	0.98 - 1.33	0.08

*Total deaths divided by denominator [1]Less than 10 deaths, rate not reported.

For peer review only

For peer review only

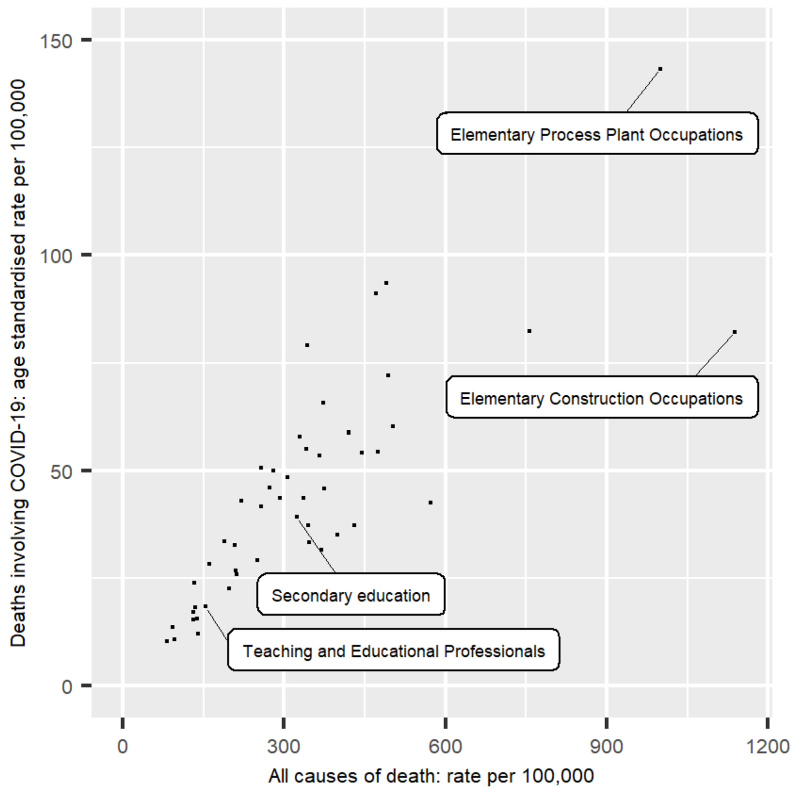

Figure 1 - Scatterplot of mortality rate (per 100,000) for Covid19 and all other causes of death for men, using age adjusted mortality rates between 9th March and 28th December 2020 from ONS. Unlabelled data points represent occupational groups in Supplementary Table S4. Pearson’s correlation coefficient = 0.78, p = 1.4 × 10-11

89x89mm (300 x 300 DPI)

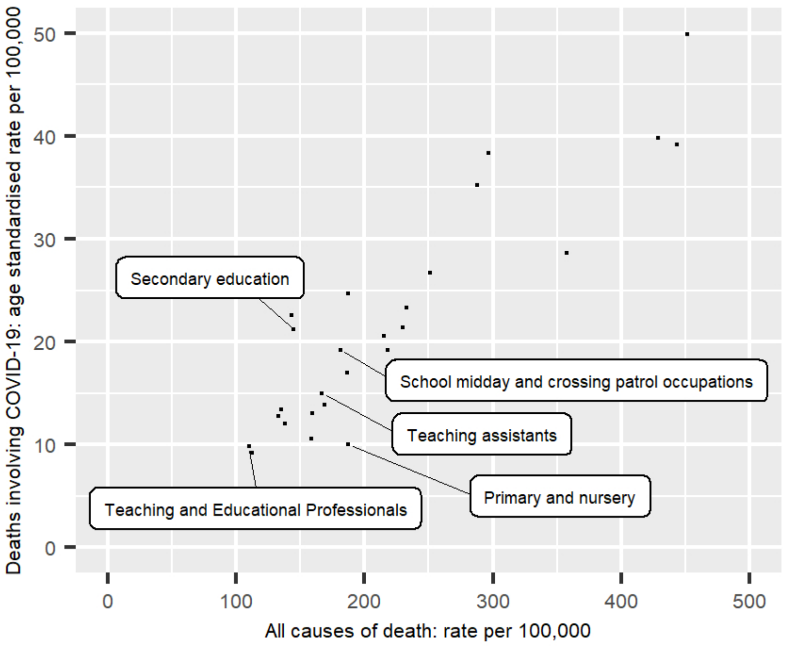

Figure 2 - Scatterplot of mortality rate (per 100,000) for Covid19 and all other causes of death for women, using age adjusted mortality rates between 9th March and 28th December 2020 from ONS. Unlabelled data points represent occupational groups in Supplementary Table S5. Pearson's correlation coefficient = 0.88, p = 1.7. × 10-09

89x89mm (300 x 300 DPI)

Supplementary material

Table S1 shows a breakdown of the number of deaths by occupation among those working in schools aged 65 and older. Excess deaths and percentage of excess deaths explained by Covid19 were calculated as for Table 2 in the main manuscript. This age group had much higher numbers of deaths compared with their 5-year average than the younger age group. Amongst all males in this age group there were 74% more deaths and amongst females there were 37% more than the 5-year average. However, only around a third of the excess deaths were thought to involve Covid19. The group of all professionals and of all occupations had fewer excess deaths as a proportion of their 5-year average but a higher proportion of the deaths involved Covid19 compared with those working in schools.

We did not have the denominators (total number working in each occupational group) for the figures presented in Tables 1s and 2s, so we were unable to work out the uncertainty in these estimates, and this will be very large in some of the smaller occupational groups**.**

Comparison of risk of death with Covid19 and from all causes for education professionals versus all professionals

Table S2 shows relative risk of deaths occurring with Covid19 for educational professionals aged 20-64 years compared to all professionals, between 9th March and 28th December 2020. Risk of dying with Covid19 in males varied from 11.5 (95%CI 5.2 to 21.7) in 100,000 for professionals in higher education to 39.2 (95%CI 24.3 to 58.6) in 100,000 for secondary school teachers. Similarly, risk in females ranged from 9.8 (95%CI 7.5 to 12.5) in 100,000 for higher education professionals to 21.2 (95% CI 12.4 to 33.2) in 100,000 among secondary school teachers. Compared with all professionals there was strong evidence that the risk of dying with Covid19 was elevated for male (RR 2.23, 95%CI 1.54 to 3.22) and female (RR 1.66, 95%CI 1.09 to 2.51) secondary school teachers. There was also evidence that the risk of dying with Covid19 was reduced among female higher education professionals (RR 0.77, 95%CI 0.60 to 0.97). For other analyses the confidence intervals were wide, and it was not possible to draw firm conclusions, although there was a suggested reduction in risk for female primary school teachers and male further education teachers.

Table S3 shows relative risks for all-cause mortality for educational professionals aged 20-64 years compared to all professionals, between 9th March and 28th December 2020. Both Male and Female higher education professionals had lower risks of mortality compared with the group of all professionals. However, there was very strong evidence that primary and secondary school teachers had higher risk of mortality from all causes compared with all professionals. This was especially true for males; male secondary school teachers had a 2.5 fold increased risk of mortality compared with all professionals and male primary school teachers a 1.8 fold increased risk.

Ratio of mortality with Covid19 versus other causes

We estimated the mortality due to all causes except Covid19 (Age adjusted all-cause mortality minus mortality with Covid19) and calculated the ratio of Covid19 mortality to mortality from other causes for minor occupational groups (according to ONS) and also for secondary school teachers and female primary school teachers. These figures are shown in Tables S4 and S5.

For peer review only

Table S4 shows the age adjusted mortality rates per 100,000 individuals for deaths with Covid19, plus our estimate of the non-Covid19 mortality per 100,000 among men. The ratio of deaths with Covid19 to other causes of death, ranged from 1 to 3 among male nurses and midwifes to 1 to 13 among elementary construction occupations. The ratio for male secondary school teachers was 1 death with Covid19 to 7 other deaths, which was similar to that for all education professionals.

Table S5 shows the age adjusted mortality rates per 100,000 individuals for deaths with Covid19, plus our estimate of the non-Covid19 mortality per 100,000 among women. The ratio of deaths with Covid19 to other causes of death, ranged from 1 to 5 among welfare professionals (social workers and probation officers) to 1 to 18 among primary and nursery school teachers. The ratio for female secondary school teachers was 1 death with Covid19 to 6 other deaths. Therefore, Covid19 was less likely to be a factor in deaths among female primary and nursery school teachers than other professions and Covid19 was more likely to primary and nursery school teachers than other professions and more likely to be involved in deaths among females secondary school teachers than among other professions except probation officers and social workers. For female teaching assistants the ratio of mortality with covid to other causes was 1:10 for school midday and crossing patrol occupations this was 1:8.

Note: There were fewer occupational groups with useable data (sufficient numbers of deaths to have age-adjusted mortality risks) for women than for men, this is due to small numbers of deaths in some groups among women.

Table S6- We calculated the proportionate mortality rates for occupational groups working in schools for which we had sufficient numbers of Covid19 deaths (n>10). This was calculated by dividing the number of deaths involving Covid19 for the occupational group by the total number of deaths in that group. We also calculated the SE of the proportion and from that the confidence intervals for the proportion using standard formula.

We found that male secondary school teachers had a proportionate mortality rate which was very similar to all males currently in work. We found that female teaching assistants and females lunchtime assistants had proportionate mortality rates which were similar to all other females currently in work. Female secondary school teachers had a proportionate mortality rate for Covid19 which was around 50% higher than for all female workers but the confidence intervals around this estimate were wide and overlapped with the estimate for all other workers. The proportionate mortality rate for female primary school teachers was 40% lower than for all female workers and the confidence intervals for this estimate did not overlap with those for all female workers.

Figure S1 and **S2** show scatterplots of mortality with Covid19 versus mortality from all causes for minor occupational among men (1s) and among women (2s). There was a very strong correlation between mortality with Covid19 and all-cause mortality among both men and women, correlation coefficients were 0.83 and 0.91 among men and women respectively with very small p-values. Risk of both Covid19 mortality and all-cause mortality among teachers and school staff were lower end of the scale. Male secondary school teachers fell within the centre of the plot, as did female teaching assistants and school midday and crossing patrol occupations, suggesting that the Covid19 risk reflects their normal mortality risk. However, both female primary and nursery school teachers and female secondary school teachers were slight outliers but in opposite directions, female primary and nursery school teachers had a low Covid19 risk relative to their all-cause mortality risk and female secondary school teachers had a slightly higher Covid19 risk relative to their all-cause mortality risk.

For peer review only

Table S1 Number of deaths among over 65s who were working in education in England and Wales 9th March to 28th December 2020

Description	Men						Women					
	Deaths involving COVID-19	All causes of death	All causes 5 year average	Excess	% excess	Excess due to Covid19	Deaths involving COVID-19	All causes of death	All causes 5 year average	Excess	% excess	Excess due to Covid19
Secondary education teaching professionals	77	565	327	238	73%	32%	19	306	226	80	35%	24%
Primary and nursery education teaching professionals	10	76	45	31	69%	32%	52	709	495	214	43%	24%
Special needs education teaching professionals	4	30	7	23	329%	17%	6	60	40	20	50%	30%
Teaching and other educational professionals n.e.c.	9	49	29	20	69%	45%	11	98	60	38	63%	29%
School secretaries	1	3	3	0	0%	NA	13	99	88	11	13%	118%
Teaching assistants	1	14	12	2	17%	50%	31	268	221	47	21%	66%
Educational support assistants	1	6	5	1	20%	20%	13	64	48	16	33%	81%
School midday and crossing patrol occupations	4	13	6	7	117%	57%	46	325	231	94	41%	49%
All those working in schools	107	756	434	322	74%	33%	191	1929	1409	520	37%	37%
All professional occupations	637	4504	3273	1231	38%	52%	401	3480	2712	768	28%	52%
All occupations	7033	43640	34676	8964	26%	78%	2993	25277	19842	5435	27%	55%

For peer review only

Table S2 - Covid19 related deaths for educational professionals aged 20-64 years compared to all professionals, between 9th March and 28th December 2020

Description	Men					Women				
	Risk	Risk for all professionals	RR	95% CI	P-value	Risk	Risk for all professionals	RR	95% CI	P-value
All educational professionals	0.00018	0.00018	1.05	0.82 - 1.34	0.74	0.000098	0.00013	0.77	0.6 - 0.97	0.03
Higher education teaching professionals	0.00012	0.00018	0.65	0.35 - 1.22	0.18	NA[1]				
Further education teaching professionals	0.00025	0.00018	1.40	0.75 - 2.62	0.29	NA[1]				
Secondary education teaching professionals	0.00039	0.00018	2.23	1.54 - 3.22	0.000025	0.00021	0.00013	1.66	1.09 - 2.51	0.017
Primary and nursery teaching professionals	NA[1]					0.00010	0.00013	0.78	0.49 - 1.23	0.29

[1]Less than 10 deaths, rate not reported in ONS data.

Table S3 – All cause mortality for education professionals aged 20-64 years compared to all professionals, between 9th March and 28th December 2020

Description	Men					Women				
	Risk	Risk for all professionals	RR	95% CI	P-value	Risk	Risk for all professionals	RR	95% CI	P-value
All educational professionals	0.0015	0.0013	1.18	1.08 - 1.28	0.00023	0.0011	0.0012	0.92	0.85 - 0.98	0.015
Higher education teaching professionals	0.00095	0.0013	0.73	0.58 - 0.92	0.007	0.00073	0.0012	0.61	0.46 - 0.81	0.00059
Further education teaching professionals	0.0017	0.0013	1.29	1.01 - 1.65	0.039	0.00096	0.0012	0.80	0.62 - 1.04	0.092
Secondary education teaching professionals	0.0032	0.0013	2.48	2.18 - 2.81	1.41×10^{-40}	0.0015	0.0012	1.21	1.03 - 1.41	0.018
Primary and nursery teaching professionals	0.0024	0.0013	1.84	1.29 - 2.61	0.00077	0.0019	0.0012	1.56	1.40 - 1.74	7.77×10^{-15}

For peer review only

Table S4 - Covid19 versus mortality from all other causes in men by minor occupational group

Group	Covid19 mortality rate	All other causes mortality rate	Ratio
Administrative Occupations: Finance	45.9	226.4	1:4.9
Administrative Occupations: Government and Related Organisations	58.6	362.2	1:6.2
Administrative Occupations: Records	33.5	155.6	1:4.6
Architects, Town Planners and Surveyors	12.1	127.8	1:10.6
Artistic, Literary and Media Occupations	22.1	248.9	1:11.3
Assemblers and Routine Operatives	32.6	175.5	1:5.4
Building Finishing Trades	42.6	529.6	1:12.4
Business, Finance and Related Associate Professionals	15.4	116.5	1:7.6
Business, Research and Administrative Professionals	13.5	79.9	1:5.9
Caring Personal Services	91	379.3	1:4.2
Construction and Building Trades	37.3	392.5	1:10.5
Construction Operatives	31.6	337.4	1:10.7
Customer Service Occupations	42.9	178.3	1:4.2
Electrical and Electronic Trades	37.3	307.5	1:8.2
Elementary Administration Occupations	54.9	286.8	1:5.2
Elementary Cleaning Occupations	54.3	419.8	1:7.7
Elementary Construction Occupations	82.1	1055.7	1:12.9
Elementary Process Plant Occupations	143.2	856.1	1:6.0
Elementary Security Occupations	93.4	396	1:4.2
Elementary Storage Occupations	54	391.4	1:7.2
Engineering Professionals	10.8	84.7	1:7.8
Functional Managers and Directors	10.3	71.3	1:6.9
Health Professionals	23.9	108.9	1:4.6
Housekeeping and Related Services	29.1	220.4	1:7.6
Information Technology and Telecommunications Professionals	15.6	122.7	1:7.9
Information Technology Technicians	26.8	183.5	1:6.8
Leisure and Travel Services	48.4	257.7	1:5.3
Managers and Directors in Retail and Wholesale	28.3	133.3	1:4.7
Managers and Directors in Transport and Logistics	50	230.3	1:4.6
Managers and Proprietors in Hospitality and Leisure Services	72	421.3	1:5.9
Managers and Proprietors in Other Services	43.5	248.8	1:5.7
Metal Machining, Fitting and Instrument Making Trades	33.2	313.6	1:9.4
Mobile Machine Drivers and Operatives	35.1	363.9	1:10.4
Nursing and Midwifery Professionals	79.1	264.2	1:3.3
Other Administrative Occupations	25.9	185.8	1:7.2
Other Drivers and Transport Operatives	43.7	292.3	1:6.7
Other Elementary Services Occupations	59	360.7	1:6.1
Other Skilled Trades	45.8	329.4	1:7.2
Plant and Machine Operatives	82.3	674.8	1:8.2
Process Operatives	65.7	307.4	1:4.7

For peer review only

Production Managers and Directors	18.1	117.4	1:6.5
Protective Service Occupations	71.2	561.9	1:7.9
Public Services and Other Associate Professionals	17	113.3	1:6.7
Road Transport Drivers	57.8	271.2	1:4.7
Sales Assistants and Retail Cashiers	53.5	312.1	1:5.8
Sales Related Occupations	41.7	215.3	1:5.2
Sales, Marketing and Related Associate Professionals	22.5	175.5	1:7.8
**Secondary education**	**39.2**	**283.8**	**1:7.2**
Teaching and Educational Professionals	18.4	135	1:7.3
Vehicle Trades	60.2	441.3	1:7.3
Welfare Professionals	50.5	206.5	1:4.1

Mortality rates are per 100,000 individuals

For peer review only

Table S5 - Covid19 versus mortality from all other causes in women by minor occupational group

Group	Covid19 mortality rate	All other causes mortality rate	Ratio
Administrative Occupations: Finance	13	146.2	1:11.2
Administrative Occupations: Government and Related Organisations	19.2	198.8	1:10.4
Assemblers and Routine Operatives	39.2	403.8	1:10.3
Caring Personal Services	38.3	258.5	1:6.7
Childcare and Related Personal Services	12.8	120	1:9.4
Customer Service Occupations	13.4	121.6	1:9.1
Elementary Cleaning Occupations	20.6	194.5	1:9.4
Elementary Process Plant Occupations	49.9	401.6	1:8.0
Elementary Security Occupations	17	169.2	1:10.0
Food Preparation and Hospitality Trades	28.6	329.2	1:11.5
Hairdressers and Related Services	39.8	388.8	1:9.8
Managers and Directors in Retail and Wholesale	26.7	224.3	1:8.4
Managers and Proprietors in Hospitality and Leisure Services	35.2	252.8	1:7.2
Managers and Proprietors in Other Services	13.9	155.4	1:11.2
Nursing and Midwifery Professionals	24.7	162.7	1:6.6
Other Administrative Occupations	12	126.5	1:10.5
Other Elementary Services Occupations	21.4	208.5	1:9.7
**Primary and nursery**	**10**	**177.5**	**1:17.8**
Sales Assistants and Retail Cashiers	23.3	209.6	1:9.0
**Secondary education**	**21.2**	**123.8**	**1:5.8**
**School midday and crossing patrol occupations**	**19.2**	**161.9**	**1:8.4**
Secretarial and Related Occupations	10.6	148.1	1:14.0
Teaching and Educational Professionals	9.8	100.2	1:10.2
**Teaching assistants**	**15**	**151.8**	**1:10.1**
Welfare and Housing Associate Professionals	9.2	103.3	1:11.2
Welfare Professionals	22.6	120.9	1:5.3

Mortality rates are per 100,000 individuals

For peer review only

Table S6- Proportionate mortality rates for those working in schools

	**Occupation**	**Covid19 deaths**	**All deaths**	**Proportionate mortality rate**
Men	Secondary teachers	29	241	0.12 (0.08 to 0.16)
	All other workers	4159	33373	0.12 (0.12 to 0.13)
Women	Primary teachers	19	327	0.06 (0.03 to 0.08)
	Secondary teachers	23	156	0.15 (0.10 t0 0.20)
	Teaching assistants	37	396	0.09 (0.07 to 0.12)
	Midday occupations	18	169	0.11 (0.06 to 0.15)
	All other workers	1669	17623	0.10 (0.09 to 0.10)

Proportionate mortality rate is number of Covid19 deaths divided by the number of deaths from all causes.

For peer review only

Figure S1 – Scatterplot of mortality rate (per 100,000) for Covid19 and all-cause mortality for men, between 9th March and 28th December 2020

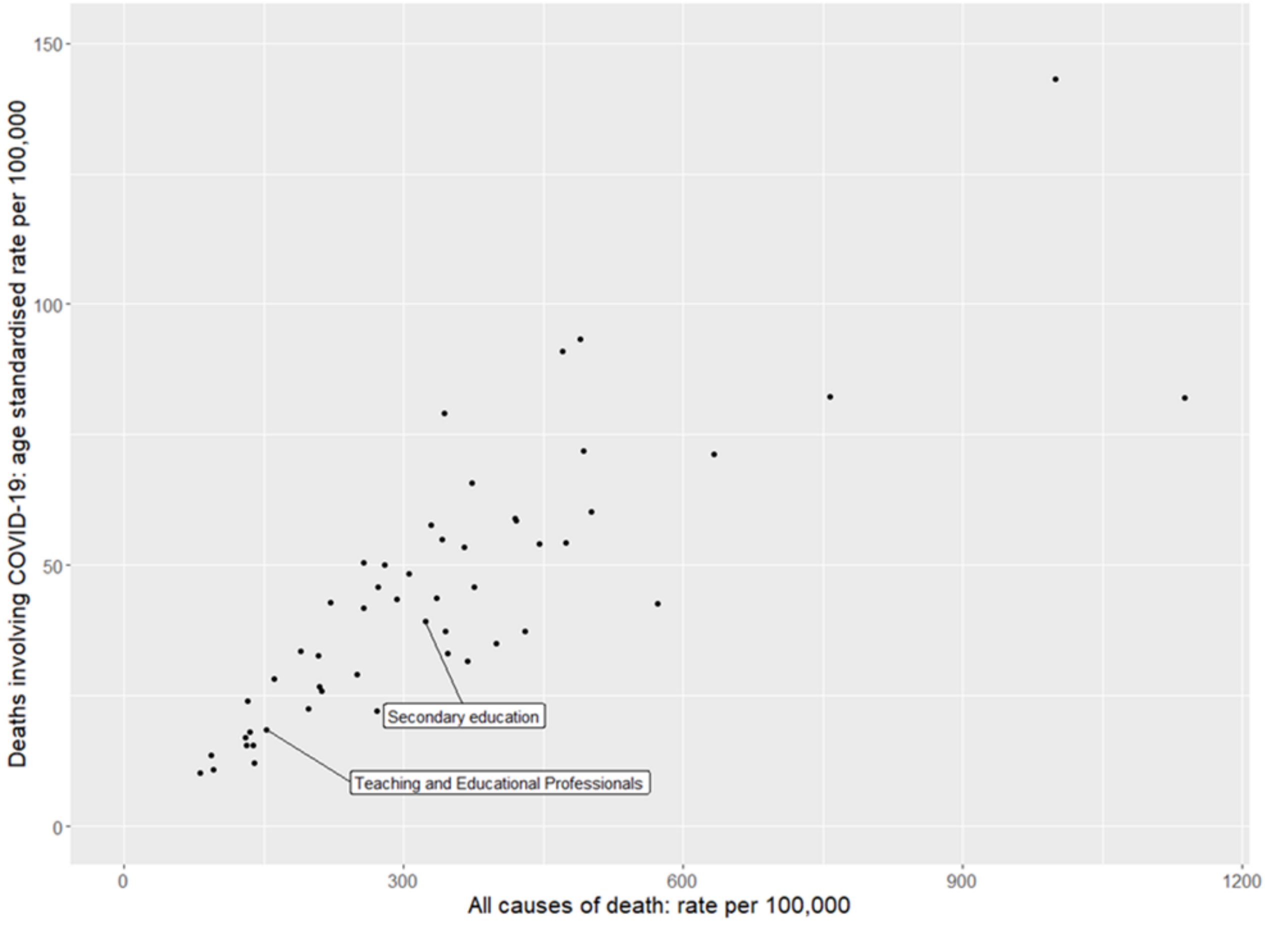

Pearson's correlation coefficient = 0.83, $p = 8.2 \times 10^{-14}$

Unlabelled data points represent other occupations in Table S4

only

Figure S2 – Scatterplot of mortality rate (per 100,000) for Covid19 and all-cause mortality for women, between 9th March and 28th December 2020

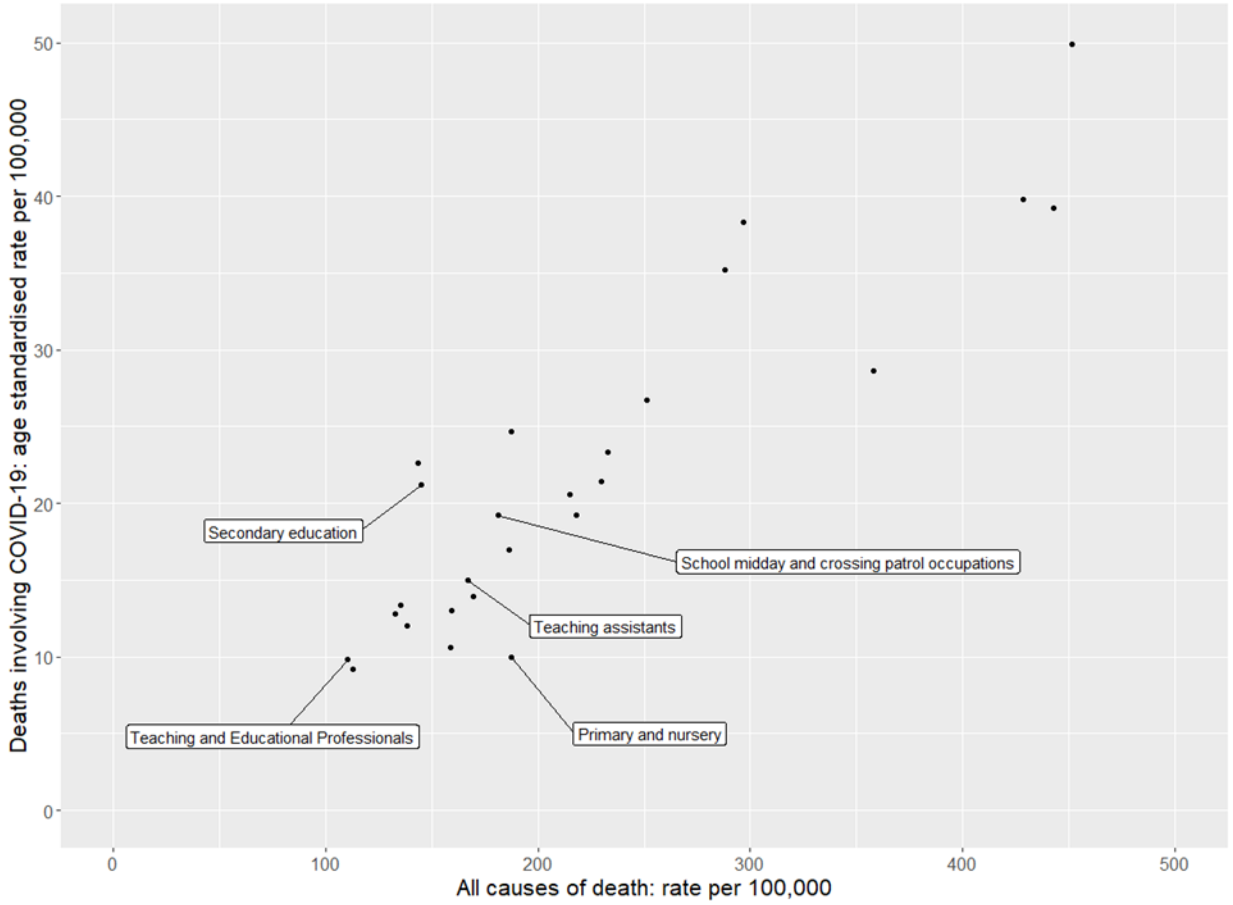

Pearson's correlation coefficient = 0.91, p = 1.4. × 10^{-10}

Unlabelled data points represent other occupations in Table S5

For peer review only

The RECORD statement – checklist of items, extended from the STROBE statement, that should be reported in observational studies using routinely collected health data.

	Item No.	STROBE items	Location in manuscript where items are reported	RECORD items	Location in manuscript where items are reported
**Title and abstract**					
	1	(a) Indicate the study's design with a commonly used term in the title or the abstract (b) Provide in the abstract an informative and balanced summary of what was done and what was found	Title (lines 2-3) and abstract (lines 14-37)	RECORD 1.1: The type of data used should be specified in the title or abstract. When possible, the name of the databases used should be included.<br><br>RECORD 1.2: If applicable, the geographic region and timeframe within which the study took place should be reported in the title or abstract.<br><br>RECORD 1.3: If linkage between databases was conducted for the study, this should be clearly stated in the title or abstract.	Title (lines 2-3) and Abstract (lines 15-19)<br><br>Reported in title (lines 1-2 and abstract 15-19)<br><br>Not applicable
**Introduction**					
Background rationale	2	Explain the scientific background and	Background (lines 54-90)		

For peer review only

		rationale for the investigation being reported			
Objectives	3	State specific objectives, including any prespecified hypotheses	Last 4 lines of the background section. (lines 93-96)		
**Methods**					
Study Design	4	Present key elements of study design early in the paper	There is a section on strength and limitations before the background (lines 39-51) and an outline of the study design in the last paragraph of the introduction (lines 90-96)		
Setting	5	Describe the setting, locations, and relevant dates, including periods of recruitment, exposure, follow-up, and data collection	This is described in the abstract and the first paragraph of the methods.		
Participants	6	*(a) Cohort study* - Give the eligibility criteria, and the sources and methods of selection of participants. Describe methods of follow-up	Not applicable- this was an ecological study using death registration data	RECORD 6.1: The methods of study population selection (such as codes or algorithms used to identify subjects) should be listed in detail. If this is not possible, an explanation	Not applicable – death registration data were used and all those eligible were represented. This is stated in the methods section and in the strength

For peer review only

		Case-control study - Give the eligibility criteria, and the sources and methods of case ascertainment and control selection. Give the rationale for the choice of cases and controls<br><br>*Cross-sectional study* - Give the eligibility criteria, and the sources and methods of selection of participants<br><br>*(b) Cohort study* - For matched studies, give matching criteria and number of exposed and unexposed<br><br>*Case-control study* - For matched studies, give matching criteria and the number of controls per case		should be provided.<br><br>RECORD 6.2: Any validation studies of the codes or algorithms used to select the population should be referenced. If validation was conducted for this study and not published elsewhere, detailed methods and results should be provided.<br><br>RECORD 6.3: If the study involved linkage of databases, consider use of a flow diagram or other graphical display to demonstrate the data linkage process, including the number of individuals with linked data at each stage.	and limitations section.
Variables	7	Clearly define all outcomes, exposures, predictors, potential confounders, and effect modifiers. Give		RECORD 7.1: A complete list of codes and algorithms used to classify exposures, outcomes, confounders, and	

For peer review only

		diagnostic criteria, if applicable.		effect modifiers should be provided. If these cannot be reported, an explanation should be provided.	
Data sources/ measurement	8	For each variable of interest, give sources of data and details of methods of assessment (measurement).<br>Describe comparability of assessment methods if there is more than one group	Provided in the methods section, Lines 121-158.		
Bias	9	Describe any efforts to address potential sources of bias	We were unable to address this in the analysis but discuss potential bias in light of other evidence in the discussion Lines 366-403		
Study size	10	Explain how the study size was arrived at	This is described in the results section, line 196-202		
Quantitative variables	11	Explain how quantitative variables were handled in the analyses. If applicable, describe which	This is explained in the methods section under the subheading: “occupational exposure		

For peer review only

		groupings were chosen, and why	groups"- Line 121		
Statistical methods	12	(a) Describe all statistical methods, including those used to control for confounding<br>(b) Describe any methods used to examine subgroups and interactions<br>(c) Explain how missing data were addressed<br>(d) *Cohort study* - If applicable, explain how loss to follow-up was addressed<br>*Case-control study* - If applicable, explain how matching of cases and controls was addressed<br>*Cross-sectional study* - If applicable, describe analytical methods taking account of sampling strategy<br>(e) Describe any sensitivity analyses	Described under the heading "statistical methods section" in the methods section, line 160<br>(b), (c) and (d) are not applicable		

For peer review only

Data access and cleaning methods		..	The dataset we used is provided as reference 9, we did not carry-out any cleaning of the data.	RECORD 12.1: Authors should describe the extent to which the investigators had access to the database population used to create the study population.<br><br>RECORD 12.2: Authors should provide information on the data cleaning methods used in the study.	In Line 94 we state that this is a publicly available datset.<br><br>Not applicable
Linkage		..		RECORD 12.3: State whether the study included person-level, institutional-level, or other data linkage across two or more databases. The methods of linkage and methods of linkage quality evaluation should be provided.	Not applicable
**Results**					
Participants	13	(a) Report the numbers of individuals at each stage of the study (*e.g.*, numbers potentially eligible, examined for eligibility,	The dataset was complete or near complete due to mandatory death registration so the numbers given in the results tables	RECORD 13.1: Describe in detail the selection of the persons included in the study (*i.e.,* study population selection) including filtering based on data	Not applicable

		confirmed eligible, included in the study, completing follow-up, and analysed)<br><br>(b) Give reasons for non-participation at each stage.<br><br>(c) Consider use of a flow diagram	represent the total eligible population.<br><br>Not applicable	quality, data availability and linkage. The selection of included persons can be described in the text and/or by means of the study flow diagram.	
Descriptive data	14	(a) Give characteristics of study participants (*e.g.*, demographic, clinical, social) and information on exposures and potential confounders<br><br>(b) Indicate the number of participants with missing data for each variable of interest<br><br>(c) *Cohort study* - summarise follow-up time (*e.g.*, average and total amount)	a), b) and c) We do not have data on this.		
Outcome data	15	*Cohort study* - Report numbers of outcome events or summary measures over time	Numbers with the outcomes of interest are reported in Table 2.		

For peer review only

		Case-control study - Report numbers in each exposure category, or summary measures of exposure<br><br>*Cross-sectional study* - Report numbers of outcome events or summary measures			
Main results	16	(a) Give unadjusted estimates and, if applicable, confounder-adjusted estimates and their precision (e.g., 95% confidence interval). Make clear which confounders were adjusted for and why they were included<br><br>(b) Report category boundaries when continuous variables were categorized<br><br>(c) If relevant, consider translating estimates of relative risk into absolute risk for a meaningful time period	a) and b) are not applicable.<br><br>c) Absolute risks are provided in table 1 and relative risks and absolute risks are provided in Tables 3 and 4.		

For peer review only

Other analyses	17	Report other analyses done—e.g., analyses of subgroups and interactions, and sensitivity analyses	All analyses are reported in the paper		
**Discussion**					
Key results	18	Summarise key results with reference to study objectives	Summarised in lines 313-336		
Limitations	19	Discuss limitations of the study, taking into account sources of potential bias or imprecision. Discuss both direction and magnitude of any potential bias	Discussed in lines 364-403	RECORD 19.1: Discuss the implications of using data that were not created or collected to answer the specific research question(s). Include discussion of misclassification bias, unmeasured confounding, missing data, and changing eligibility over time, as they pertain to the study being reported.	Discussed in lines 364-400
Interpretation	20	Give a cautious overall interpretation of results considering objectives, limitations, multiplicity of analyses, results from similar studies, and	Provided in the conclusion lines 404-411		

For peer review only

		other relevant evidence			
Generalisability	21	Discuss the generalisability (external validity) of the study results	Discussed in Lines 385-387		
**Other Information**					
Funding	22	Give the source of funding and the role of the funders for the present study and, if applicable, for the original study on which the present article is based	Provided under the heading "funding" line 421.		
Accessibility of protocol, raw data, and programming code		..	A reference and a link to the raw data are provided.	RECORD 22.1: Authors should provide information on how to access any supplemental information such as the study protocol, raw data, or programming code.	A supplementary material section is provided.

*Reference: Benchimol EI, Smeeth L, Guttmann A, Harron K, Moher D, Petersen I, Sørensen HT, von Elm E, Langan SM, the RECORD Working Committee. The REporting of studies Conducted using Observational Routinely-collected health Data (RECORD) Statement. *PLoS Medicine* 2015; in press.

*Checklist is protected under Creative Commons Attribution (CC BY) license.

BMJ Open

Was the risk of death among the population of teachers and other school workers in England and Wales due to Covid19 and all causes higher than other occupations during the pandemic in 2020? An ecological study using routinely collected data on deaths from the Office for National Statistics

Journal:	*BMJ Open*
Manuscript ID	bmjopen-2021-050656.R2
Article Type:	Original research
Date Submitted by the Author:	24-Sep-2021
Complete List of Authors:	Lewis, Sarah; University of Bristol, Population Health Sciences<br>Dack, Kyle; University of Bristol, Population Health Sciences<br>Relton, Caroline; University of Bristol,<br>Munafo, Marcus; University of Bristol, Experimental Psychology<br>Davey Smith, George; University of Bristol, MRC- Integrative Epidemiology Unit
**Primary Subject Heading**:	Epidemiology
Secondary Subject Heading:	Occupational and environmental medicine
Keywords:	COVID-19, OCCUPATIONAL & INDUSTRIAL MEDICINE, INFECTIOUS DISEASES

SCHOLARONE™
Manuscripts

I, the Submitting Author has the right to grant and does grant on behalf of all authors of the Work (as defined in the below author licence), an exclusive licence and/or a non-exclusive licence for contributions from authors who are: i) UK Crown employees; ii) where BMJ has agreed a CC-BY licence shall apply, and/or iii) in accordance with the terms applicable for US Federal Government officers or employees acting as part of their official duties; on a worldwide, perpetual, irrevocable, royalty-free basis to BMJ Publishing Group Ltd ("BMJ") its licensees and where the relevant Journal is co-owned by BMJ to the co-owners of the Journal, to publish the Work in this journal and any other BMJ products and to exploit all rights, as set out in our licence.

The Submitting Author accepts and understands that any supply made under these terms is made by BMJ to the Submitting Author unless you are acting as an employee on behalf of your employer or a postgraduate student of an affiliated institution which is paying any applicable article publishing charge ("APC") for Open Access articles. Where the Submitting Author wishes to make the Work available on an Open Access basis (and intends to pay the relevant APC), the terms of reuse of such Open Access shall be governed by a Creative Commons licence – details of these licences and which Creative Commons licence will apply to this Work are set out in our licence referred to above.

Other than as permitted in any relevant BMJ Author's Self Archiving Policies, I confirm this Work has not been accepted for publication elsewhere, is not being considered for publication elsewhere and does not duplicate material already published. I confirm all authors consent to publication of this Work and authorise the granting of this licence.

For peer review only

Was the risk of death among the population of teachers and other school workers in England and Wales due to Covid19 and all causes higher than other occupations during the pandemic in 2020? An ecological study using routinely collected data on deaths from the Office for National Statistics

Sarah J Lewis[1,2], Kyle Dack[1,2], Caroline L Relton[1,2], Marcus R Munafò[1,3] and George Davey Smith[1,2]

1.MRC Integrative Epidemiology Unit at the University of Bristol

2. School of Population Health Sciences, University of Bristol

3. School of Psychological Science, University of Bristol

Corresponding Author: Sarah J Lewis (s.j.lewis@bristol.ac.uk)

Abstract

Objectives: To estimate occupation risk from Covid19 among teachers and others working in schools using publicly available data on mortality in England and Wales.

Design: Analysis of national death registration data from the Office for National Statistics.

Setting: England and Wales, 8th March to 28th December 2020, during the Covid19 pandemic.

Participants: The total working age population in England and Wales plus those still working aged over 65.

Primary and Secondary outcomes: Death with Covid19 as a primary outcome and death from all causes as a secondary outcome.

Results: Across occupational groups there was a strong correlation between Covid19 mortality and both non-Covid19 and all-cause mortality. The absolute mortality rates for deaths with Covid19 were low amongst those working in schools (from 10 per 100,000 in female primary school teachers to 39 per 100,000 male secondary school teachers) relative to many other occupations (range: 10 to 143 per 100,000 in men; 9 to 50 per 100,000 in women).There was weak evidence that secondary school teachers had slightly higher risks of dying with Covid19 compared to the average for all working aged people, but stronger evidence of a higher risk compared to the average for all professionals; primary school teachers had a lower risk. All-cause mortality was also higher amongst all teachers compared to all professionals. Teaching and lunchtime assistants were not at higher risk of death from Covid19 compared with all working aged people.

Conclusion: There was weak evidence that Covid19 mortality risk for secondary school teachers was above expectation, but in general school staff had Covid19 mortality risks which were proportionate to their non-covid mortality risk.

For peer review only

Strengths and limitations of this study

- We used routinely collected data on all deaths in England and Wales; , which are near-complete and not susceptible to serious ascertainment biases.
- We were able to compare mortality data for teachers and other school workers with all other occupational groups and with the working-age population.
- The number of deaths due to Covid19 were small and thus differences between the specific occupational groups were imprecisely estimated.
- We did not have access to individual level mortality data so were not able to account for potential confounders such as comorbidities or household size.
- For those working in school aged over 65 we had neither mortality rates per 100,000 nor total numbers within the group; we only had number of deaths and a 5-year average and we do not know whether the denominators have changed for this group over the last 5 years.

Background

School closures have been implemented in many countries in an effort to slow the spread of the SARS-CoV-2 virus responsible for the Covid19 pandemic. This has affected hundreds of millions of children globally and has been contentious due to harms that have arisen to children as a result of school closures (1). Schools in England and Wales closed to most pupils (except a small number of children of critical workers and some vulnerable children) on the 20th March 2020 and did not reopen fully until September 2020; they closed again for the Christmas holidays (between 11th and 18th December) and reopened on the 8th March 2021 in England. In Wales the reopening of schools after Christmas was more gradual and started on the 22nd Feb 2021 with the youngest children, with all children returning by the 15th April 2021.

Whether or not teachers and others working in schools are at higher risk from Covid19 as a result of schools being open is central to decisions on school reopening, but until recently there has not been good data on this. Record linkage studies carried out in Scotland (2) and Finland (3) show that teachers had a greater risk of being diagnosed as a Covid19 case compared with the general population when schools were open; there was a 40% higher risk of Covid19 among teachers in the Scottish study, and between 50% and 70% higher risk in the Finnish study. A study in Sweden found that teachers delivering in person teaching had a two-fold higher risk of Covid-19 infection compared to those who were teaching remotely (4). However, studies examining transmission in schools have found this to occur at low levels (5,6). One reason for the discrepancy is that teachers are likely to be under greater surveillance, and therefore undergo more testing than those working in other occupations. In the Scottish study teachers were much more likely to be tested over the autumn term compared with the general population, and this is likely to explain at least some the elevated number of cases among teachers.

Analyses of more robust outcomes such as hospitalisation from Covid19 are less susceptible to detection bias and may be more reliable. The Scottish study (2) found teachers and their household members were at a lower risk of being hospitalised with Covid19 compared with adults of working age in the general population after matched on age, sex and general practice registration and adjusted for ethnicity, deprivation, comorbidity counts and sharing a household with a healthcare worker. Sweden has been an outlier in the Covid19 pandemic in that schools have remained open for children up to 16 years of age. A study by the Swedish Public Health Agency found that 20 out of 103,596 school teachers in Sweden

For peer review only

received intensive care treatment up to the 30th June 2020, an age and sex matched analysis comparing teachers with other occupations (except health care workers) reported an OR of 0.43 (95% CI 0.28 to 0.68) (7). Similarly, a study carried out in Norway did not find higher rates of hospitalisation with Covid19 for school teachers, after adjustment for age, sex and country of birth (8). There is an absence of evidence on the risk to teaching and lunchtime assistants working in schools.

The Office for National Statistics (ONS) routinely collect mortality data for those living in England and Wales. They released data on deaths with Covid19 and all-cause mortality by occupation as a publicly available dataset (9). We used this data to compared mortality risk among school workers and all working aged adults in England and Wales. We also compared mortality rates among teachers to all professionals, because there are large differences in mortality risk by occupation, largely driven by differences in socioeconomic circumstances (10).

Methods

The ONS mortality statistics used for this analysis are based on mandatory registration of all deaths in England and Wales. So far 3 datasets on mortality with Covid19 by occupation have been released; the first covered the period from 8th March to the 20th April which included the peak of wave 1 deaths, the second covered the period between 8th March to the 25th May and the final dataset (the one we used for the analysis in this paper) covered the period from 8th March to the 28th December.

We used these data to: 1) describe the mortality rates among all educational professionals combined and present these alongside rates for all professionals and all working aged people for the different time periods covered by this data (Table 1) , 2) compare the number of deaths with historical data on deaths for the same occupational group over 5 years (2015 to 2019) for all school workers and for individual occupations (teachers, teaching assistants, school secretaries etc), which are presented alongside death rates among all occupations and all professional occupations over the same period of the pandemic (Table 2 and S1), 3) calculate the risk of Covid19 and all cause mortality among teachers and other school staff compared with all working aged people (Table 3 and 4) and all professionals (Supplementary Table S2 and S3) and finally, 4) we investigated the ratio of mortality with Covid19 to mortality from other causes among different occupational groups to determine whether school staff were outliers (Supplementary Table S4-S6 and all Figures).

Occupational exposure groups

Occupation was reported on the death certificate at the time of death registration by the informant and was coded according to the ONS standard occupational classification 2010 (11). Population counts for occupational groups were estimated from the Annual Population Survey (APS), conducted in 2019 (12). The APS is the largest ongoing household survey in the UK, conducting interviews with members of randomly selected households.

The group ‘teaching and educational professionals’, as defined by the ONS, includes; higher education teaching professionals, further education teaching professionals, secondary education teaching professionals, primary and nursery education teaching professionals, special needs education teaching professionals, senior professionals of educational establishments, education advisers and school inspectors and teaching and other educational professionals not elsewhere defined.

The group ‘teaching and educational professionals’ does not include: teaching assistants, school secretaries, lunchtime assistants or educational support assistants, but these

For peer review only

occupations were considered along with teachers working in schools where sufficient data were available.

The group defined by ONS as midday and crossing patrol occupations, includes some individuals who work as road crossing patrols, however not all schools have these and if they do usually only have one, so the group will mainly comprise of those who supervise children in school during their lunch break and who prepare school lunch. Therefore midday and crossing patrol occupations will be shorted and referred to in this manuscript as lunchtime assistants.

The comparison groups we used were:

1) All working aged people – for comparisons of mortality rates and risk presented in Tables 1, 3 and 4.
2) All occupations – for comparisons of number of deaths presented in Tables 2 and S1.
3) All professional occupations – for comparisons of mortality risk presented in Tables S2 and S3.

Professional occupations are those which are classified by ONS as major occupational group 2, these are occupations which require a degree or equivalent period of relevant work experience and include, but are not limited to; scientists, engineers, architects, doctors, nurses, radiographers, physiotherapists, social workers and solicitors, for further information see reference 11.

Outcome definition

ONS define deaths with Covid19 as those where Covid19 or suspected Covid19 was mentioned anywhere on the death certificate, including in combination with other health conditions. If a death certificate mentions Covid19, it will not always be the main cause of death but may be a contributory factor.

Statistical analysis

We calculated the relative risk of death for education professions compared to all working aged (20 to 64 year olds) people and compared teachers to all professionals in England and Wales stratified by sex, for this we used published ONS data on total deaths and age adjusted mortality rates per 100,000 for each group. We derived the denominator (total population) for each group of interest as

Risk =mortality rate per 100,000 taken from ONS data/100,000.

We calculated 95% confidence intervals and *p*-values using the formula;

$$CI = \exp(\ln(RR) \pm 1.96 \times \sqrt{1/deaths + 1/control\ deaths - 1/population - 1/control\ population})$$

$$P = \exp((-0.717 \times (RR/SE)) - (0.416 \times (RR/SE)^2))$$

The ONS data included deaths for 8 subgroups of educational professionals, we included those with a sufficient number of Covid19 deaths (≥10) in our analysis. In addition, sufficient data were available to include female teaching and lunchtime assistants in this analysis. We also performed a fixed effect meta-analysis across the 4 occupational groups working in schools among females.

For peer review only

We estimated mortality rates for all other causes (other than Covid19) by calculating the difference between all-cause mortality and mortality with Covid19 for each minor occupational group as defined by ONS (9, 11).

We produced scatterplots of ‘all cause’ versus ‘with Covid19’ mortality and ‘all other causes’ versus ‘with Covid19’ mortality by occupational group for men and women separately and calculated Pearson’s correlation coefficient for these plots.

We also calculated proportionate mortality ratios as mortality rates from Covid19 divided by all-cause mortality for occupations working in schools.

Finally, we used our estimates from above for the SE and the denominators for each occupational group to estimate the variance for Covid19 mortality. We then performed a weighted least squares regression analysis (weighted by 1/variance) of Covid19 mortality against other cause to determine the increase in Covid19 for 1 death increase in other cause mortality across occupational groups.

Patient and Public Involvement statement

The public were not involved in the design or conduct of this study.

Ethical approval

Ethical approval was not sought for this project because we used publicly accessible summary statistics of a national mandatory data collection.

Results

Death rates from Covid19 and all causes among all education professionals presented alongside death rates for all professionals and all working aged people by time period.

Between the 8th March 2020 and 28th December 2020 there were 68,757 deaths from all causes among the working age (20-64 years) population in England and Wales, 7,961 (12%) of these deaths involved Covid19, of which, 2,494 had occurred prior to the 20th April, 2,267 occurred between 20th April and the 25th May, and 3,200 occurred between the 25th May and the 28th December 2020.

There were 1,326 deaths from all causes among educational professionals during this period; 139 of those deaths were thought to involve Covid19.

Table 1

Covid19 was involved in 1 in 8 male deaths and 1 in 9 female deaths among working aged people; among education professionals these figures were approximately 1 in 8 deaths among males and 1 in 11 among females. Covid19 was involved in a higher proportion of deaths early on in the pandemic; data covering the 6 weeks of the first peak in the UK showed that Covid19 was involved in 1 in 4 deaths in males and 1 in 6 deaths in female education professionals during this time. During the 10 months covered by the ONS data all-cause and Covid19 mortality rates among educational professionals appeared to be lower than for all working aged adults and similar to those for all professionals.

Number of deaths by individual occupations and combined across all school workers compared with 5-year average rates

We examined number of deaths by occupation among those working in schools aged 20-64 years (Table 2) and among those aged 65 or older (Supplementary table S1). Among

For peer review only

working aged people when deaths across all occupations working in schools were combined, we observed 15% more deaths (excess) compared with the 2015-2019 average for the same period among men and 5% more among women. For women this was less than the excess observed across all occupations and among all professionals; for men it was similar. Deaths with Covid19 appeared to account for almost all the excess among men whereas among women there were twice as many deaths with Covid19 among school workers as there were excess deaths.

Among the older age group there were much higher numbers of deaths compared with their 5-year average (74% more deaths amongst males and 37% among females). However, only 33% of excess deaths in men and 37% in women were thought to involve Covid19. The group of all professionals and of all occupations had fewer excess deaths as a proportion of their 5-year average but a higher proportion of the deaths involved Covid19 compared with those working in schools.

Comparison of Covid19 and all-cause deaths among education professionals and those working in schools compared to the working aged population

Table 3 shows that compared to the total working aged population there was strong evidence that the risk of dying with Covid19 was lower among all education professionals combined (RR_{Males} 0.59, 95%CI 0.46 to 0.75, $RR_{females}$ 0.58, 95%CI 0.46 to 0.73). There was also some evidence of a lower risk of death with Covid19 among female primary and nursery education professionals (RR 0.60, 95%CI 0.38 to 0.93). For both male and female secondary school teachers the relative risk estimate shows a 25% higher risk of death from Covid19, although there was a great deal of uncertainty around these estimates (RR_{Males} 1.25, 95%CI 0.87 to 1.80, $RR_{females}$ 1.26, 95%CI 0.84 to 1.90). There was little evidence of a difference in risk between teaching assistants, or lunchtime assistants compared with working aged people. A fixed effect meta-analysis across the 4 occupational groups working in schools in women showed that risk was similar to all working aged people (RR 0.95, 95CI 0.77 to 1.14), with weak evidence of heterogeneity across the groups (I^2=55.2%, Chi^2=6.697, p=0.08).

Table 4 shows strong evidence that the risk of death from all causes during this pandemic period was lower for higher and further education teacher professionals compared with the working aged population of England and Wales. The was also evidence that male secondary school teachers (RR 1.26, 95%CI 1.11 to 1.43) and female primary school teachers (RR 1.18, 95%CI 1.06 to 1.32) had slightly higher mortality risk compared to the general population.

We also calculated relative risks for mortality with Covid19 and all-cause mortality in educational professionals versus all professionals (Supplementary Tables S2 and S3). We found strong evidence that both male and female secondary school teachers had a higher risk of mortality with Covid19 compared with all professionals. In addition male and female primary and secondary school teachers had higher mortality risks for all causes compared with all professionals.

Comparison of Covid19 to other cause mortality across occupational groups

Figures 1 and 2 show that there are strong correlations between all other cause mortality and mortality with Covid19. Weighted least squares regression analysis showed that for every death from other causes there were 0.11 (95%CI=0.09 to 0.13, $p=2.0\times10^{-16}$) deaths from Covid19, with no meaningful difference between men (0.11, 95%CI 0.09 to 0.13, $p=2.49\times10^{-12}$) and women (0.10, 95%CI 0.08 to 0.13, $p=4.41\times10^{-9}$).

We also plotted mortality with Covid19 versus all-cause mortality on separate scatterplots (Figures S1 and S2) and found similar results.

Ratio of mortality with Covid19 to other causes

The ratio of deaths with Covid19 to other causes of death, ranged from 1:3 among male nurses and midwifes to 1:13 among male elementary construction occupations (Supplementary Table S4). The ratio of Covid19 to other causes of death for male secondary school teachers was 1:7, which was the same as the average for all male professionals and all working aged men. Among women (Supplementary Table S5), the ratio of mortality with Covid19 to other causes of death, ranged from 1:5 among welfare professionals (social workers and probation officers) to 1:18 among primary and nursery schoolteachers; ratios for female secondary school teachers, teaching assistants and lunchtime assistants were 1:6, 1:10 and 1:8 respectively. Proportionate mortality rates (Covid19 deaths divided by deaths from all causes) are presented in Supplementary Table S6.

Discussion

Covid19 deaths among education professionals and others working in schools

We used routinely collected data on mortality for the Office of National Statistics in the UK to study deaths with Covid19 among teachers and other school staff, during the pandemic in 2020. We found that all educational professionals combined had fewer deaths per 100,000 occurring with Covid19 compared to the general population. This is to be expected since the total working aged population of England and Wales will include those who are unable to work due to ill health. The group 'education professionals' includes those working in universities and school inspectors, many of whom have been working from home throughout the pandemic. We therefore investigated excess deaths specifically among school teachers and other staff working in schools and compared them to all those currently working and all professionals. There were fewer deaths than the 5-year average among female primary and secondary school teachers and the number of deaths among male teachers were similar to the 5-year average. There were more deaths among teaching assistants compared to the 5-year average, but only around half of the excess deaths were thought to involve Covid19. For all women working in schools combined there was a small increase in the number of deaths (5%) compared with the 5-year average, but the number of deaths occurring with Covid19 was greater than the excess, suggesting that some of those dying with Covid19 may have died over the course of a normal year from other causes. For those working in schools who were aged 65 and over there were large excesses in deaths compared with the average for the previous 5 years (74% for men and 37% for women), but only around a third of the excess deaths were thought to involve Covid19. It is possible that some excess deaths, were due to undiagnosed Covid19 among those who did not have classic symptoms (13), but this is less likely for later deaths as the amount of testing for Covid19 increased dramatically over time in the UK. We do not have information on other causes of death within this population over this time period, however in 2019 the most common causes of death among over 65s in England and Wales were heart disease and cancer (14). It is possible that more school staff were dying from other causes during this period due to delayed treatments for other conditions or due to an unwillingness to seek help for fear of contracting Covid19 or of overburdening the healthcare system. It is also possible the number of school staff in this group has increased over the last 5 years. Further research is needed to determine whether these represent numerator-denominator bias or if there is a true excesses and if so the cause of these excess deaths.

For peer review only

Relative risk of death with Covid19 among school staff

We found that secondary school teachers had around a 25% higher risk (in both men and women) of dying with Covid19 compared with the general population, although the evidence was weak. There was stronger evidence of an increased risk to secondary school teachers compared to all professionals. However, all-cause mortality was also increased among secondary school teachers compared to all professionals.

Proportionate mortality ratios (mortality from a specific cause: ‘all cause’ or ‘all other cause mortality’) are routinely used in occupational epidemiology in situations where calculation and adequate standardization of mortality rate ratios is not possible (15). The rationale for doing this is that if an occupation has a particular risk for a specific disease the proportion of deaths due to that disease will be increased relative to deaths from other causes in the group. We therefore plotted the correlation between age adjusted mortality for all causes and mortality with Covid19 across minor occupational groups for whom risk was reported in the ONS dataset, the two measures are bound to be correlated to a certain extent because all-cause mortality includes with Covid19. However, we also found a strong positive correlation between mortality with Covid19 and non-Covid19. This is consistent with findings from others which show that deaths from Covid19 closely match ‘normal’ risk within the UK population (13,16). Mortality from any cause amongst all education professionals and teachers were towards the lower end of the distribution. For male secondary teachers, female teaching and lunchtime assistants the ratio of mortality with Covid19 to all-cause mortality was consistent with other occupational groups; female primary school teachers and female secondary school teachers appeared to be outliers in opposite directions, however the number of deaths in these groups was small and a meta-analysis across occupations working in school showed only weak evidence of heterogeneity.

Consistency with other studies

The data we examined from ONS is consistent with the Public Health Scotland study (2) and with the Swedish study (7) which both showed similar or lower risk of hospitalisations due to Covid19 among teachers compared with other occupations, but our findings are also compatible with a slightly higher risk of Covid19 mortality among secondary school teachers. Mutambudzi and colleagues (17) investigated the risk of hospitalisation or death by occupation after testing positive for Covid19 among 120,075 working participants (aged 49 to 64 years) in the UK Biobank study, they found weak evidence of more hospitalisations or deaths with Covid19 among education workers compared to non-essential workers up to July 2020 (OR 1.59, 95%CI 0.87 to 2.91) after adjusting for potential confounders. However, UK Biobank is a highly selected population, which is not representative of the UK population due to a response rate of just 5% (18).

A study of data on teacher absence due to Covid19 in England during the Autumn term (September-December) 2020 found that the proportion of teachers absent due to infection appeared to be similar in both primary and secondary schools (19).The ONS schools infection survey found that primary and secondary staff had similar antibody positivity rates for SARS-CoV2 (15% and 16% respectively) at the end of Autumn term, which reflected exposure during the time when in person teaching was taking place; positivity rates among all teachers were slightly lower than among all working aged adults (18%) (20). Despite this, primary school teachers in England and Wales had a lower risk of death with Covid19 compared to secondary school teachers (although confidence intervals were overlapping).

Very few studies have examined risk among teaching assistants and lunchtime assistants as separate groups; we found that despite more deaths occurring in this group in 2020

For peer review only

compared to the previous 5 years, they were not at increased risk of death due to Covid19 or any cause compared to working aged people. A study carried out in California used modelling to compare deaths from all causes during the pandemic and found an odds ratio for teaching assistants of 1.28, but no confidence intervals were provided for that study (21).

Strengths and limitations of the study

We used routinely collected data on mortality which includes all deaths in England and Wales, and results are therefore unlikely to be due to ascertainment bias and will be representative of the working aged population of these countries. However, we did not have access to individual level mortality data so were not able to account for potential confounders such as comorbidities or household size. For our relative risk calculations our comparison group also included school staff (although they only made up a very small proportion of the total) because we did not have age adjusted mortality rates excluding teachers. The age adjusted mortality rates calculated by ONS were based on denominators take from a survey conducted in 2019, if the school workforce has changed as a result of the pandemic these rates will be inaccurate, if any changes in the school workforce are systematically different to other professions the results will be biased. Whilst the size of the school workforce in England remained fairly stable between 2015-2019, there was a marked decrease in the number of teachers retiring (from 17,853 teachers retiring in 2015 to 12,062 teachers in 2019) (22), suggesting that the average age of teachers may have increased over this time period. We don't know what impact the pandemic has had on the size and age distribution of those working in schools, it could be that older more vulnerable staff have retired or conversely staff may have stayed on to help with staff shortages due to teachers having to isolate. Therefore, we were unable to adequately assess the extent to which risk of death had changed during the pandemic period for these occupational groups especially among those aged over 65. In addition, the number of deaths were extremely small and subject to random fluctuations in some groups. Whilst our results are likely to be generalisable to countries which are similar to the UK, they may not be generalisable to developing countries and countries with different social structures.

In the period covered by this data many school staff worked remotely for large periods of time during 2020, a survey carried out by the global education company Tes found that just 22% of teachers were engaged in face-to-face teaching during the first lockdown (23),The more vulnerable staff are likely to have worked from home even when schools were fully open, which will have an impact on their workplace exposure. However, all teachers were potentially exposed to Covid19 up until schools closed on 20th March 2020 and deaths up to 25th May, just two months after schools were closed, accounted for approximately two thirds of all deaths occurring over the study period (Table 1). Given the lag between infection and death, many people dying up to the 25th May could have been infected prior to school closures. We do not have data on deaths by date and occupation, however, out of a total of 68,186 deaths which occurred due to Covid19 in England and Wales between the dates covered by this data (24), only 5018 deaths (7.4%) occurred between 25th May 2020 and the 30th September 2020, a period during which those dying were unlikely to have been infected in school due to school closures. Therefore the vast majority of people dying of Covid19 during the period covered by the study could have been infected when schools were open.

In Conclusion

Teachers, teaching and lunchtime assistants aged 20-64 years were not at high risk of death relative to the working age population in England and Wales during the Covid19 pandemic. For occupations working in schools Covid19 mortality was generally proportionate to all-cause mortality. Female primary school teachers and female secondary school teachers

For peer review only - http://bmjopen.bmj.com/site/about/guidelines.xhtml

seemed to be outliers in opposite directions however, evidence for heterogeneity in Covid19 mortality risk among school workers was weak. For policy makers who are considering the impact of schools being open in future pandemics it will be important to note that staff were not at high risk of death compared to other occupations. Although we did not address these in this manuscript, other outcomes such as hospitalisation due to Covid19 and long covid are also important considerations. In addition, for Covid19 the emergence of more infectious strains since the data for this study were collected plus vaccination of school staff are likely to have changed the situation in schools.

Contributorship statement – SL and GDS conceived the idea for the manuscript and designed the analyses, SL and KD performed the analyses, SL, GDS, CR and MM all contributed to the interpretation of the results, all authors contributed to drafting the manuscript and have seen and approved the final version

Competing interests – The authors have no competing interests.

Funding- Sarah Lewis, Caroline Relton and George Davey Smith have received a grant from National Institute for Health Research (NIHR) and UK Research and Innovation (UKRI)COVID-19 mapping and mitigation in schools (CoMMinS)

Data sharing statement-The data used in this manuscript have been made publicly available by the Office of National Statistics (ONS) in the United Kingdom

For peer review only

References

1. Buonsenso D, Roland D, De Rose C, Vásquez-Hoyos P, Ramly B, Chakakala-Chaziya JN, Munro A, González-Dambrauskas S. Schools Closures During the COVID-19 Pandemic: A Catastrophic Global Situation. Pediatr Infect Dis J. 2021;40(4):e146-e150. doi: 10.1097/INF.0000000000003052.

2. Fenton L, Gribben C, Caldwell D, Colville S, Bishop J, Reid M, White J, Hutchinson S, Robertson C, Colhoun HM, Wood R, McKeigue PM, McAllister DA. Risk of hospital admission with covid-19 among teachers compared with healthcare workers and other adults of working age in Scotland, March 2020 to July 2021: population based case-control study. *BMJ*. 2021;374:n2060. doi:10.1136/bmj.n2060

3. Helsinki Gradate School of Economics. Situation Room Report: The Corona Virus and Health Differences – In Which Socioeconomic Groups Have the Most Infections Been Observed in Finland? 19th January, 2021. https://www.helsinkigse.fi/covid19-data-en/situation-room-report-the-corona-virus-and-health-differences-in-which-socioeconomic-groups-have-the-most-infections-been-observed-in-finland/

4. Vlachos J, Hertegård E, Svalery HB. The effects of school closures on SARS-CoV-2 among parents and teachers. Proceedings of the National Academy of Sciences Mar 2021, 118 (9) e2020834118; DOI: 10.1073/pnas.2020834118

5. Ismail SA, Saliba V, Lopez Bernal J, Ramsay ME, Ladhani SN. SARS-CoV-2 infection and transmission in educational settings: a prospective, cross-sectional analysis of infection clusters and outbreaks in England. Lancet Infect Dis. 2021 Mar;21(3):344-353. doi: 10.1016/S1473-3099(20)30882-3

6. Falk A, Benda A, Falk P, Steffen S, Wallace Z, Høeg TB. COVID-19 Cases and Transmission in 17 K–12 Schools — Wood County, Wisconsin, August 31–November 29, 2020. MMWR Morb Mortal Wkly Rep 2021;70:136–140. DOI: http://dx.doi.org/10.15585/mmwr.mm7004e3

7. Ludvigsson JF, Engerström L, Nordenhäll C, Larsson E. Open Schools, Covid-19, and Child and Teacher Morbidity in Sweden. N Engl J Med. 2021;384(7):669-71 doi: 10.1056/NEJMc2026670.

8. Magnusson K, Nygård K, Vold L, Telle K. Occupational risk of COVID-19 in the 1st vs 2nd wave of infection. MedRxiv *Preprints* https://www.medrxiv.org/content/10.1101/2020.10.29.20220426v1.full.pdf

9. Coronavirus (COVID-19) related deaths by occupation, England and Wales. Release date 25th January 2021. https://www.ons.gov.uk/peoplepopulationandcommunity/healthandsocialcare/causesofdeath/datasets/coronaviruscovid19relateddeathsbyoccupationenglandandwales

10. Katikireddi SV, Leyland AH, McKee M, Ralston K, Stuckler D. Patterns of mortality by occupation in the UK, 1991-2011: a comparative analysis of linked census and mortality records. Lancet Public Health. 2017 Oct 23;2(11):e501-e512. doi: 10.1016/S2468-2667(17)30193-7.

11. Office for National Statistics. SOC2010 volume 1: structure and descriptions of unit groups. https://www.ons.gov.uk/methodology/classificationsandstandards/standardoccupationalclassificationsoc/soc2010/soc2010volume1structureanddescriptionsofunitgroups

For peer review only

12. Office for National Statistics. Annual population survey (APS) QMI. https://www.ons.gov.uk/employmentandlabourmarket/peopleinwork/employmentandemployeetypes/methodologies/annualpopulationsurveyapsqmi

13. Spiegelhalter D. Use of “normal” risk to improve understanding of dangers of covid-19. BMJ 2020; 370: m3259. https://doi.org/10.1136/bmj.m3259

14. Office for National Statistics. Deaths registered in England and Wales. Release date July 2020. https://www.ons.gov.uk/peoplepopulationandcommunity/birthsdeathsandmarriages/deaths/datasets/deathsregisteredinenglandandwalesseriesdrreferencetables

15. Decouflé P, Thomas TL, Pickle LW. Comparison of the proportionate mortality ratio and standardized mortality ratio risk measures. Am J Epidemiol. 1980 Mar;111(3):263-9. doi: 10.1093/oxfordjournals.aje.a112895.

16. . Bhaskaran K, Bacon S, Evans SJ, Bates CJ, Rentsch CT, MacKenna B, Tomlinson L, Walker AJ, Schultze A, Morton CE, Grint D, Mehrkar A, Eggo RM, Inglesby P, Douglas IJ, McDonald HI, Cockburn J, Williamson EJ, Evans D, Curtis HJ, Hulme WJ, Parry J, Hester F, Harper S, Spiegelhalter D, Smeeth L, Goldacre B. Factors associated with deaths due to COVID-19 versus other causes: population-based cohort analysis of UK primary care data and linked national death registrations within the OpenSAFELY platform. Lancet Reg Health Eur. 2021;6:100109. doi: 10.1016/j.lanepe.2021.100109.

17. Mutambudzi M, Niedwiedz C, Macdonald EB, et al. Occupation and risk of severe COVID-19: prospective cohort study of 120 075 UK Biobank participants. Occupational and Environmental Medicine 2021;78:307-314.doi: 10.1136/oemed-2020-106731. https://oem.bmj.com/content/early/2020/12/01/oemed-2020-106731.full#DC1

18. Tyrrell J, Zheng J, Beaumont, R. *et al.* Genetic predictors of participation in optional components of UK Biobank. *Nat Commun* **12,** 886 (2021). https://doi.org/10.1038/s41467-021-21073-y

19. Southall E, Holmes A, Hill EM, Atkins BD, Leng T, Thompson RN, Dyson L, Keeling MJ, Tildesley MJ. An analysis of school absences in England during the Covid-19 pandemic. medRxiv 2021.02.10.21251484 [Preprint]; doi: Southall E, Holmes A, Hill EM, Atkins BD, Leng T, Thompson RN, Dyson L, Keeling MJ, Tildesley MJ. An analysis of school absences in England during the COVID-19 pandemic. BMC Med. 2021 Jun 7;19(1):137. doi: 10.1186/s12916-021-01990-x..

20. Office for National Statistics. COVID-19 Schools Infection Survey Round 2, England: December 2020. https://www.ons.gov.uk/peoplepopulationandcommunity/healthandsocialcare/conditionsanddiseases/bulletins/covid19schoolsinfectionsurveyround2england/december2020#main-points

21. Chen Y-H, Glymour M, Riley A, Balmes J, Duchowny K, Harrison R, Matthay E, Bibbins-Domingo K. Excess mortality associated with the COVID-19 pandemic among Californians 18–65 years of age, by occupational sector and occupation: March through October 2020. medRxiv 2021.01.21.21250266 [Preprint]; doi: https://doi.org/10.1101/2021.01.21.21250266

22. United Kingdom Government. Reporting Year 2019. School workforce I n England. https://explore-education-statistics.service.gov.uk/find-statistics/school-workforce-in-england#dataBlock-4189d4c5-ba35-4f48-9248-cc39f53703a2-tables

23. Tes. Reality of lockdown for school staff: 5 key findings. https://www.tes.com/news/reality-lockdown-school-staff-5-key-findings

24. Gov.uk. Coronavirus (COVID-19) in the UK. https://coronavirus.data.gov.uk/

For peer review only

Table legends

Table 1 Age adjusted all-cause and with Covid19 mortality per 100,000 population (95% confidence intervals) taken from 3 ONS datasets on Covid19 mortality by occupation covering different time periods during the 2020 Covid19 pandemic. Incudes the ratio of Covid19 deaths to all-cause deaths for education professionals, all professionals and all working aged people (aged 20-64 years) stratified by sex.

Table 2- Deaths among working aged (20-64) people who working in education in England and Wales 9th March to 28th December 2020. This table shows the number of deaths by occupation in 2020 and the average for the previous 5 years among those working in schools aged 20-64 years, also includes figures for all occupations and all professionals for comparison. The number of excess deaths was calculated by subtracting the 5 year average number of deaths (provided by ONS) from the number of deaths from all causes between 9th March and 28th December 2020 for each occupation and occupational group. The number of excess deaths was converted into a percentage of the 5-year average (percentage excess). The percentage excess explained by Covid19 was calculated by dividing the number of Covid19 deaths by the number of excess deaths and multiplying this by 100. We set the maximum percentage excess deaths to be 100, but we did not cap the percentage excess explained by Covid19. The reason for this is to determine whether Covid19 could be a factor in some deaths which may have occurred anyway.

Table 3 Relative risks for death occurring with Covid19 for school staff aged 20-64 years compared to the total working aged population, calculated using age-adjusted mortality rates by occupation between 9th March and 28th December 2020 from the ONS dataset.

Table 4 Relative risks for death occurring from all causes for school staff aged 20-64 years compared to the total working aged population, calculated using age-adjusted mortality rates by occupation between 9th March and 28th December 2020 from the ONS dataset.

Figure legends

Figure 1 Scatterplot of mortality rate (per 100,000) for Covid19 and all other causes of death for men, using age adjusted mortality rates between 9th March and 28th December 2020 from ONS. Unlabelled data points represent occupational groups in Supplementary Table S4. Pearson's correlation coefficient = 0.78, $p = 1.4 \times 10^{-11}$

Figure 2 Scatterplot of mortality rate (per 100,000) for Covid19 and all other causes of death for women, using age adjusted mortality rates between 9th March and 28th December 2020 from ONS. Unlabelled data points represent occupational groups in Supplementary Table S5. Pearson's correlation coefficient = 0.88, $p = 1.7. \times 10^{-09}$

For peer review only

For peer review only

Table 1- Age adjusted all cause and with Covid19 mortality rates per 100,000 population (95% confidence intervals) for all educational professionals, all professional occupations and all working aged adults in England and Wales by overlapping time periods covered by this data.

Occupational group	Cause of Mortality	Males			Females		
		8th March-20th April	8th March-25th May	8th March-28th December	8th March-20th April	8th March-25th May	8th March-28th December
Working aged adults	With Covid19	9.9 (9.4-10.4)	19.1 (18.4-19.8)	31.4 (30.6-32.3)	5.2 (4.9-5.6)	9.7 (9.3-10.2)	16.8 (16.2-17.5)
	All cause	41.6 (40.6-42.6)	78.1 (76.7-79.4)	256.0 (253.5-258.4)	26.3 (25.5-27.1)	48.4 (47.3-49.4)	158.3 (156.4-160.2)
	Ratio*	1:4.2	1:4.1	1:8.2	1:5.1	1:5.0	1:9.4
Professional Occupations	With Covid19	5.6 (4.6-6.6)	11.6 (10.2-13.0)	17.6 (15.9 - 19.3)	4.2 (3.3-5.2)	8.0 (6.8 to 9.3)	12.8 (11.2- 14.4)
	All cause	22.2 (20.2-24.1)	41.2 (38.5-43.8)	130.4 (125.7-135.2)	22.0 (19.9-24.1)	39.3 (36.5-42.1)	120.0 (115.2-124.9)
	Ratio*	1:4	1:3.6	1:7.4	1:5.2	1:4.9	1:9.4
Educational professionals	With Covid19	6.7 (4.1-10.3)	12.9 (9.3-17.4)	18.4 (14.0-23.6)	3.3 (2.0-4.9)	6.0 (4.2-8.1)	9.8 (7.5-12.5)
	All cause	28.0 (22.4-34.5)	48.1 (40.4-55.7)	153.4 (139.8-167.1	20.4 (16.9-24.0)	37.2 (32.5-42.0)	110.0 (101.9-118.2)
	Ratio*	1:4.2	1:3.7	1:8.3	1:6.2	1:6.2	1:11.2

*Ratio of deaths involving Covid19 deaths to all deaths

For peer review only

Table 2 Number of deaths among working aged (20-64) people who were working in education in England and Wales 9th March to 28th December 2020

Description	Men						Women					
	Deaths involving COVID-19	All causes of death	All causes 5 year average	Excess	% excess	Excess due to Covid19	Deaths involving COVID-19	All causes of death	All causes 5 year average	Excess	% excess	Excess due to Covid19
Secondary education teaching professionals	29	241	233	8	3%	100%	23	156	173	-17	-10%	NA
Primary and nursery education teaching professionals	4	31	30	1	3%	100%	19	327	368	-41	-11%	NA
Special needs education teaching professionals	1	12	8	4	50%	25%	3	27	33	-6	-18%	NA
Teaching and other educational professionals n.e.c.	8	56	32	14	44%	57%	7	70	61	9	15%	78%
School secretaries	0	3	2	1	50%	0%	4	58	53	5	9%	80%
Teaching assistants	5	28	19	9	47%	56%	37	396	307	89	29%	42%

For peer review only

**Educational support assistants**	1	7	8	-1	-13%	NA	3	55	58	-3	-5%	NA
**School midday and crossing patrol occupations**	2	11	5	6	120%	33%	18	169	149	20	13%	90%
**All those working in schools**	50	389	337	52	15%	96%	114	1258	1202	56	5%	204%
**All professional occupations**	419	3144	2765	379	14%	111%	279	2696	2477	219	9%	127%
**All occupations**	4225	33904	29745	4159	14%	102%	1742	18419	16528	1891	11%	92%

For peer review only

Table 3 Covid19 deaths for educational professions aged 20-64 years compared to all working aged adults, between 9th March and 28th December 2020

**Description**	**Men**					**Women**				
	Risk*	Risk for all workers	RR	95% CI	P-value	Risk*	Risk for all workers	RR	95% CI	P-value
All educational professionals	0.00018	0.00031	0.59	0.46 - 0.75	0.000018	0.000098	0.00017	0.58	0.46 - 0.73	0.0000058
Higher education teaching professionals	0.00012	0.00031	0.37	0.20 - 0.68	0.0016	NA[1]				
Further education teaching professionals	0.00025	0.00031	0.79	0.42 - 1.46	0.46	NA[1]				
Secondary education teaching professionals	0.00039	0.00031	1.25	0.87 - 1.80	0.23	0.00021	0.00017	1.26	0.84 - 1.90	0.27
Primary and nursery teaching professionals	NA[1]					0.00010	0.00017	0.60	0.38 - 0.93	0.024

Teaching assistants	NA[1]					0.00015	0.00017	0.89	0.65 - 1.23	0.5
Lunchtime assistants	NA[1]					0.00019	0.00017	1.14	0.72 - 1.82	0.58
[*1]Less than 10 deaths, rate not reported.										

For peer review only

Table 4 All-cause mortality for school staff aged 20-64 years compared to all working aged adults, between 9th March and 28th December 2020

Description	Men					Women				
	Risk*	Risk for all workers	RR	95% CI	P-value	Risk*	Risk for all workers	RR	95% CI	P-value
All educational professionals	0.0015	0.0026	0.60	0.55 - 0.65	1.74×10^{-29}	0.0011	0.0016	0.69	0.65 - 0.74	6.14×10^{-23}
Higher education teaching professionals	0.00095	0.0026	0.37	0.30 - 0.47	2.00×10^{-16}	0.00073	0.0016	0.46	0.35 - 0.61	1.13×10^{-07}
Further education teaching professionals	0.0017	0.0026	0.66	0.52 - 0.84	0.00077	0.00096	0.0016	0.61	0.47 - 0.79	0.00015
Secondary education teaching professionals	0.0032	0.0026	1.26	1.11 - 1.43	0.00033	0.0015	0.0016	0.92	0.78 - 1.07	0.28
Primary and nursery teaching professionals	0.0024	0.0026	0.94	0.66 - 1.33	0.72	0.0019	0.0016	1.18	1.06 - 1.32	0.0023
Teaching assistants	0.0017	0.0026	0.65	0.45 - 0.95	0.024	0.0017	0.0016	1.05	0.95 - 1.16	0.3
Lunchtime assistants	NA[1]					0.0018	0.0016	1.14	0.98 - 1.33	0.08

*Total deaths divided by denominator [1]Less than 10 deaths, rate not reported.

For peer review only

For peer review only

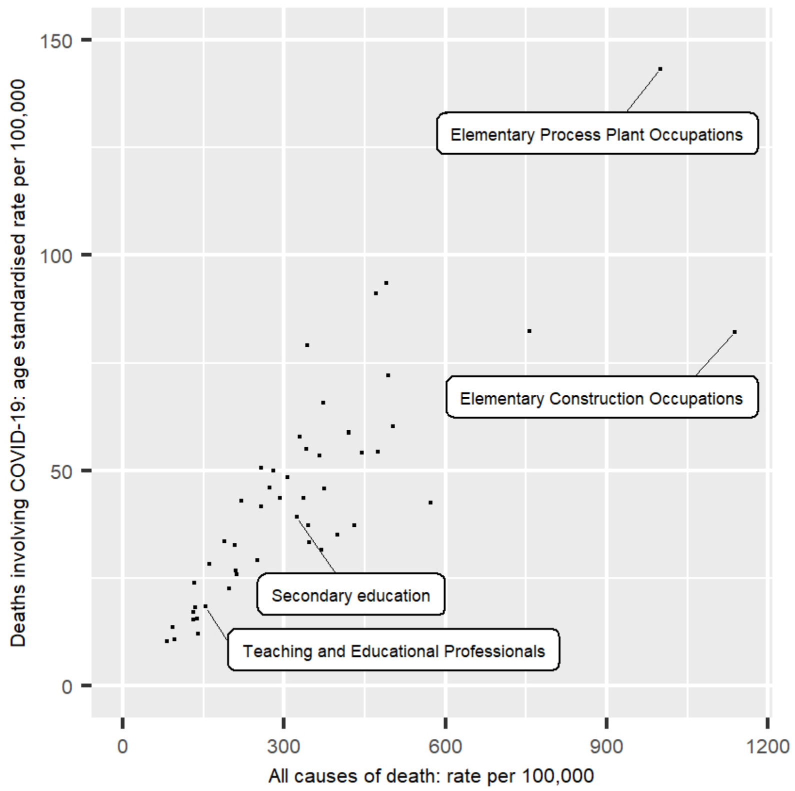

Figure 1 - Scatterplot of mortality rate (per 100,000) for Covid19 and all other causes of death for men, using age adjusted mortality rates between 9th March and 28th December 2020 from ONS. Unlabelled data points represent occupational groups in Supplementary Table S4. Pearson's correlation coefficient = 0.78, p = 1.4 × 10-11

89x89mm (300 x 300 DPI)

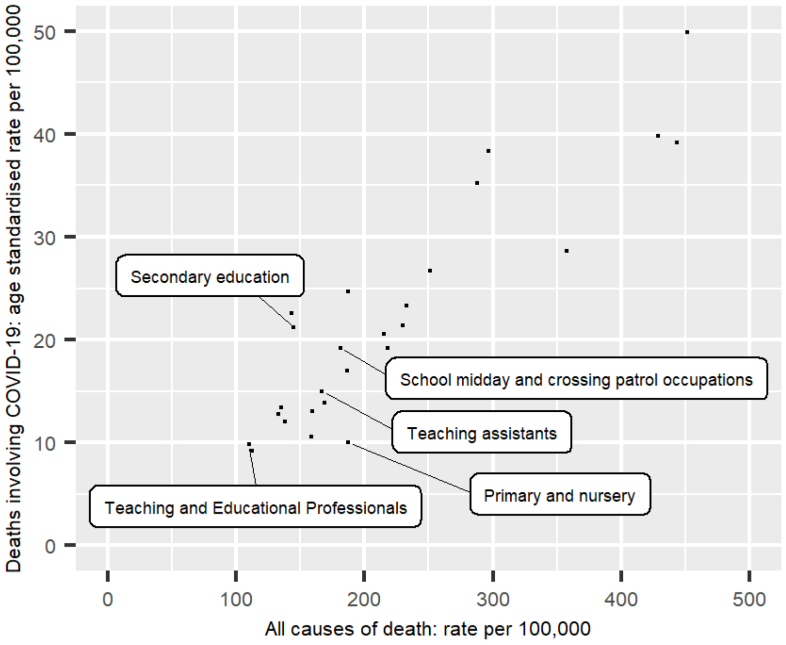

Figure 2 - Scatterplot of mortality rate (per 100,000) for Covid19 and all other causes of death for women, using age adjusted mortality rates between 9th March and 28th December 2020 from ONS. Unlabelled data points represent occupational groups in Supplementary Table S5. Pearson's correlation coefficient = 0.88, p = 1.7. × 10-09

89x89mm (300 x 300 DPI)

Supplementary material

Table S1 shows a breakdown of the number of deaths by occupation among those working in schools aged 65 and older. Excess deaths and percentage of excess deaths explained by Covid19 were calculated as for Table 2 in the main manuscript. This age group had much higher numbers of deaths compared with their 5-year average than the younger age group. Amongst all males in this age group there were 74% more deaths and amongst females there were 37% more than the 5-year average. However, only around a third of the excess deaths were thought to involve Covid19. The group of all professionals and of all occupations had fewer excess deaths as a proportion of their 5-year average but a higher proportion of the deaths involved Covid19 compared with those working in schools.

We did not have the denominators (total number working in each occupational group) for the figures presented in Tables 1s and 2s, so we were unable to work out the uncertainty in these estimates, and this will be very large in some of the smaller occupational groups**.**

Comparison of risk of death with Covid19 and from all causes for education professionals versus all professionals

Table S2 shows relative risk of deaths occurring with Covid19 for educational professionals aged 20-64 years compared to all professionals, between 9th March and 28th December 2020. Risk of dying with Covid19 in males varied from 11.5 (95%CI 5.2 to 21.7) in 100,000 for professionals in higher education to 39.2 (95%CI 24.3 to 58.6) in 100,000 for secondary school teachers. Similarly, risk in females ranged from 9.8 (95%CI 7.5 to 12.5) in 100,000 for higher education professionals to 21.2 (95% CI 12.4 to 33.2) in 100,000 among secondary school teachers. Compared with all professionals there was strong evidence that the risk of dying with Covid19 was elevated for male (RR 2.23, 95%CI 1.54 to 3.22) and female (RR 1.66, 95%CI 1.09 to 2.51) secondary school teachers. There was also evidence that the risk of dying with Covid19 was reduced among female higher education professionals (RR 0.77, 95%CI 0.60 to 0.97). For other analyses the confidence intervals were wide, and it was not possible to draw firm conclusions, although there was a suggested reduction in risk for female primary school teachers and male further education teachers.

Table S3 shows relative risks for all-cause mortality for educational professionals aged 20-64 years compared to all professionals, between 9th March and 28th December 2020. Both Male and Female higher education professionals had lower risks of mortality compared with the group of all professionals. However, there was very strong evidence that primary and secondary school teachers had higher risk of mortality from all causes compared with all professionals. This was especially true for males; male secondary school teachers had a 2.5 fold increased risk of mortality compared with all professionals and male primary school teachers a 1.8 fold increased risk.

Ratio of mortality with Covid19 versus other causes

We estimated the mortality due to all causes except Covid19 (Age adjusted all-cause mortality minus mortality with Covid19) and calculated the ratio of Covid19 mortality to mortality from other causes for minor occupational groups (according to ONS) and also for secondary school teachers and female primary school teachers. These figures are shown in Tables S4 and S5.

For peer review only

Table S4 shows the age adjusted mortality rates per 100,000 individuals for deaths with Covid19, plus our estimate of the non-Covid19 mortality per 100,000 among men. The ratio of deaths with Covid19 to other causes of death, ranged from 1 to 3 among male nurses and midwifes to 1 to 13 among elementary construction occupations. The ratio for male secondary school teachers was 1 death with Covid19 to 7 other deaths, which was similar to that for all education professionals.

Table S5 shows the age adjusted mortality rates per 100,000 individuals for deaths with Covid19, plus our estimate of the non-Covid19 mortality per 100,000 among women. The ratio of deaths with Covid19 to other causes of death, ranged from 1 to 5 among welfare professionals (social workers and probation officers) to 1 to 18 among primary and nursery school teachers. The ratio for female secondary school teachers was 1 death with Covid19 to 6 other deaths. Therefore, Covid19 was less likely to be a factor in deaths among female primary and nursery school teachers than other professions and Covid19 was more likely to primary and nursery school teachers than other professions and more likely to be involved in deaths among females secondary school teachers than among other professions except probation officers and social workers. For female teaching assistants the ratio of mortality with covid to other causes was 1:10 for school midday and crossing patrol occupations this was 1:8.

Note: There were fewer occupational groups with useable data (sufficient numbers of deaths to have age-adjusted mortality risks) for women than for men, this is due to small numbers of deaths in some groups among women.

Table S6- We calculated the proportionate mortality rates for occupational groups working in schools for which we had sufficient numbers of Covid19 deaths (n>10). This was calculated by dividing the number of deaths involving Covid19 for the occupational group by the total number of deaths in that group. We also calculated the SE of the proportion and from that the confidence intervals for the proportion using standard formula.

We found that male secondary school teachers had a proportionate mortality rate which was very similar to all males currently in work. We found that female teaching assistants and females lunchtime assistants had proportionate mortality rates which were similar to all other females currently in work. Female secondary school teachers had a proportionate mortality rate for Covid19 which was around 50% higher than for all female workers but the confidence intervals around this estimate were wide and overlapped with the estimate for all other workers. The proportionate mortality rate for female primary school teachers was 40% lower than for all female workers and the confidence intervals for this estimate did not overlap with those for all female workers.

Figure S1 and **S2** show scatterplots of mortality with Covid19 versus mortality from all causes for minor occupational among men (1s) and among women (2s). There was a very strong correlation between mortality with Covid19 and all-cause mortality among both men and women, correlation coefficients were 0.83 and 0.91 among men and women respectively with very small p-values. Risk of both Covid19 mortality and all-cause mortality among teachers and school staff were lower end of the scale. Male secondary school teachers fell within the centre of the plot, as did female teaching assistants and school midday and crossing patrol occupations, suggesting that the Covid19 risk reflects their normal mortality risk. However, both female primary and nursery school teachers and female secondary school teachers were slight outliers but in opposite directions, female primary and nursery school teachers had a low Covid19 risk relative to their all-cause mortality risk and female secondary school teachers had a slightly higher Covid19 risk relative to their all-cause mortality risk.

For peer review only

Table S1 Number of deaths among over 65s who were working in education in England and Wales 9th March to 28th December 2020

Description	Men						Women					
	Deaths involving COVID-19	All causes of death	All causes 5 year average	Excess	% excess	Excess due to Covid19	Deaths involving COVID-19	All causes of death	All causes 5 year average	Excess	% excess	Excess due to Covid19
Secondary education teaching professionals	77	565	327	238	73%	32%	19	306	226	80	35%	24%
Primary and nursery education teaching professionals	10	76	45	31	69%	32%	52	709	495	214	43%	24%
Special needs education teaching professionals	4	30	7	23	329%	17%	6	60	40	20	50%	30%
Teaching and other educational professionals n.e.c.	9	49	29	20	69%	45%	11	98	60	38	63%	29%
School secretaries	1	3	3	0	0%	NA	13	99	88	11	13%	118%
Teaching assistants	1	14	12	2	17%	50%	31	268	221	47	21%	66%
Educational support assistants	1	6	5	1	20%	20%	13	64	48	16	33%	81%
School midday and crossing patrol occupations	4	13	6	7	117%	57%	46	325	231	94	41%	49%
All those working in schools	107	756	434	322	74%	33%	191	1929	1409	520	37%	37%
All professional occupations	637	4504	3273	1231	38%	52%	401	3480	2712	768	28%	52%
All occupations	7033	43640	34676	8964	26%	78%	2993	25277	19842	5435	27%	55%

For peer review only

Table S2 - Covid19 related deaths for educational professionals aged 20-64 years compared to all professionals, between 9th March and 28th December 2020

Description	Men					Women				
	Risk	Risk for all professionals	RR	95% CI	P-value	Risk	Risk for all professionals	RR	95% CI	P-value
All educational professionals	0.00018	0.00018	1.05	0.82 - 1.34	0.74	0.000098	0.00013	0.77	0.6 - 0.97	0.03
Higher education teaching professionals	0.00012	0.00018	0.65	0.35 - 1.22	0.18	NA[1]				
Further education teaching professionals	0.00025	0.00018	1.40	0.75 - 2.62	0.29	NA[1]				
Secondary education teaching professionals	0.00039	0.00018	2.23	1.54 - 3.22	0.000025	0.00021	0.00013	1.66	1.09 - 2.51	0.017
Primary and nursery teaching professionals	NA[1]					0.00010	0.00013	0.78	0.49 - 1.23	0.29
[1]Less than 10 deaths, rate not reported in ONS data.										

Table S3 – All cause mortality for education professionals aged 20-64 years compared to all professionals, between 9th March and 28th December 2020

Description	Men					Women				
	Risk	Risk for all professionals	RR	95% CI	P-value	Risk	Risk for all professionals	RR	95% CI	P-value
All educational professionals	0.0015	0.0013	1.18	1.08 - 1.28	0.00023	0.0011	0.0012	0.92	0.85 - 0.98	0.015
Higher education teaching professionals	0.00095	0.0013	0.73	0.58 - 0.92	0.007	0.00073	0.0012	0.61	0.46 - 0.81	0.00059
Further education teaching professionals	0.0017	0.0013	1.29	1.01 - 1.65	0.039	0.00096	0.0012	0.80	0.62 - 1.04	0.092
Secondary education teaching professionals	0.0032	0.0013	2.48	2.18 - 2.81	1.41×10^{-40}	0.0015	0.0012	1.21	1.03 - 1.41	0.018
Primary and nursery teaching professionals	0.0024	0.0013	1.84	1.29 - 2.61	0.00077	0.0019	0.0012	1.56	1.40 - 1.74	7.77×10^{-15}

Table S4 - Covid19 versus mortality from all other causes in men by minor occupational group

Group	Covid19 mortality rate	All other causes mortality rate	Ratio
Administrative Occupations: Finance	45.9	226.4	1:4.9
Administrative Occupations: Government and Related Organisations	58.6	362.2	1:6.2
Administrative Occupations: Records	33.5	155.6	1:4.6
Architects, Town Planners and Surveyors	12.1	127.8	1:10.6
Artistic, Literary and Media Occupations	22.1	248.9	1:11.3
Assemblers and Routine Operatives	32.6	175.5	1:5.4
Building Finishing Trades	42.6	529.6	1:12.4
Business, Finance and Related Associate Professionals	15.4	116.5	1:7.6
Business, Research and Administrative Professionals	13.5	79.9	1:5.9
Caring Personal Services	91	379.3	1:4.2
Construction and Building Trades	37.3	392.5	1:10.5
Construction Operatives	31.6	337.4	1:10.7
Customer Service Occupations	42.9	178.3	1:4.2
Electrical and Electronic Trades	37.3	307.5	1:8.2
Elementary Administration Occupations	54.9	286.8	1:5.2
Elementary Cleaning Occupations	54.3	419.8	1:7.7
Elementary Construction Occupations	82.1	1055.7	1:12.9
Elementary Process Plant Occupations	143.2	856.1	1:6.0
Elementary Security Occupations	93.4	396	1:4.2
Elementary Storage Occupations	54	391.4	1:7.2
Engineering Professionals	10.8	84.7	1:7.8
Functional Managers and Directors	10.3	71.3	1:6.9
Health Professionals	23.9	108.9	1:4.6
Housekeeping and Related Services	29.1	220.4	1:7.6
Information Technology and Telecommunications Professionals	15.6	122.7	1:7.9
Information Technology Technicians	26.8	183.5	1:6.8
Leisure and Travel Services	48.4	257.7	1:5.3
Managers and Directors in Retail and Wholesale	28.3	133.3	1:4.7
Managers and Directors in Transport and Logistics	50	230.3	1:4.6
Managers and Proprietors in Hospitality and Leisure Services	72	421.3	1:5.9
Managers and Proprietors in Other Services	43.5	248.8	1:5.7
Metal Machining, Fitting and Instrument Making Trades	33.2	313.6	1:9.4
Mobile Machine Drivers and Operatives	35.1	363.9	1:10.4
Nursing and Midwifery Professionals	79.1	264.2	1:3.3
Other Administrative Occupations	25.9	185.8	1:7.2
Other Drivers and Transport Operatives	43.7	292.3	1:6.7
Other Elementary Services Occupations	59	360.7	1:6.1
Other Skilled Trades	45.8	329.4	1:7.2
Plant and Machine Operatives	82.3	674.8	1:8.2
Process Operatives	65.7	307.4	1:4.7

For peer review only

Production Managers and Directors	18.1	117.4	1:6.5
Protective Service Occupations	71.2	561.9	1:7.9
Public Services and Other Associate Professionals	17	113.3	1:6.7
Road Transport Drivers	57.8	271.2	1:4.7
Sales Assistants and Retail Cashiers	53.5	312.1	1:5.8
Sales Related Occupations	41.7	215.3	1:5.2
Sales, Marketing and Related Associate Professionals	22.5	175.5	1:7.8
**Secondary education**	**39.2**	**283.8**	**1:7.2**
Teaching and Educational Professionals	18.4	135	1:7.3
Vehicle Trades	60.2	441.3	1:7.3
Welfare Professionals	50.5	206.5	1:4.1

Mortality rates are per 100,000 individuals

For peer review only

Table S5 - Covid19 versus mortality from all other causes in women by minor occupational group

Group	Covid19 mortality rate	All other causes mortality rate	Ratio
Administrative Occupations: Finance	13	146.2	1:11.2
Administrative Occupations: Government and Related Organisations	19.2	198.8	1:10.4
Assemblers and Routine Operatives	39.2	403.8	1:10.3
Caring Personal Services	38.3	258.5	1:6.7
Childcare and Related Personal Services	12.8	120	1:9.4
Customer Service Occupations	13.4	121.6	1:9.1
Elementary Cleaning Occupations	20.6	194.5	1:9.4
Elementary Process Plant Occupations	49.9	401.6	1:8.0
Elementary Security Occupations	17	169.2	1:10.0
Food Preparation and Hospitality Trades	28.6	329.2	1:11.5
Hairdressers and Related Services	39.8	388.8	1:9.8
Managers and Directors in Retail and Wholesale	26.7	224.3	1:8.4
Managers and Proprietors in Hospitality and Leisure Services	35.2	252.8	1:7.2
Managers and Proprietors in Other Services	13.9	155.4	1:11.2
Nursing and Midwifery Professionals	24.7	162.7	1:6.6
Other Administrative Occupations	12	126.5	1:10.5
Other Elementary Services Occupations	21.4	208.5	1:9.7
**Primary and nursery**	**10**	**177.5**	**1:17.8**
Sales Assistants and Retail Cashiers	23.3	209.6	1:9.0
**Secondary education**	**21.2**	**123.8**	**1:5.8**
**School midday and crossing patrol occupations**	**19.2**	**161.9**	**1:8.4**
Secretarial and Related Occupations	10.6	148.1	1:14.0
Teaching and Educational Professionals	9.8	100.2	1:10.2
**Teaching assistants**	**15**	**151.8**	**1:10.1**
Welfare and Housing Associate Professionals	9.2	103.3	1:11.2
Welfare Professionals	22.6	120.9	1:5.3

Mortality rates are per 100,000 individuals

For peer review only

Table S6- Proportionate mortality rates for those working in schools

	Occupation	Covid19 deaths	All deaths	Proportionate mortality rate
Men	Secondary teachers	29	241	0.12 (0.08 to 0.16)
	All other workers	4159	33373	0.12 (0.12 to 0.13)
Women	Primary teachers	19	327	0.06 (0.03 to 0.08)
	Secondary teachers	23	156	0.15 (0.10 t0 0.20)
	Teaching assistants	37	396	0.09 (0.07 to 0.12)
	Midday occupations	18	169	0.11 (0.06 to 0.15)
	All other workers	1669	17623	0.10 (0.09 to 0.10)

Proportionate mortality rate is number of Covid19 deaths divided by the number of deaths from all causes.

For peer review only

Figure S1 – Scatterplot of mortality rate (per 100,000) for Covid19 and all-cause mortality for men, between 9th March and 28th December 2020

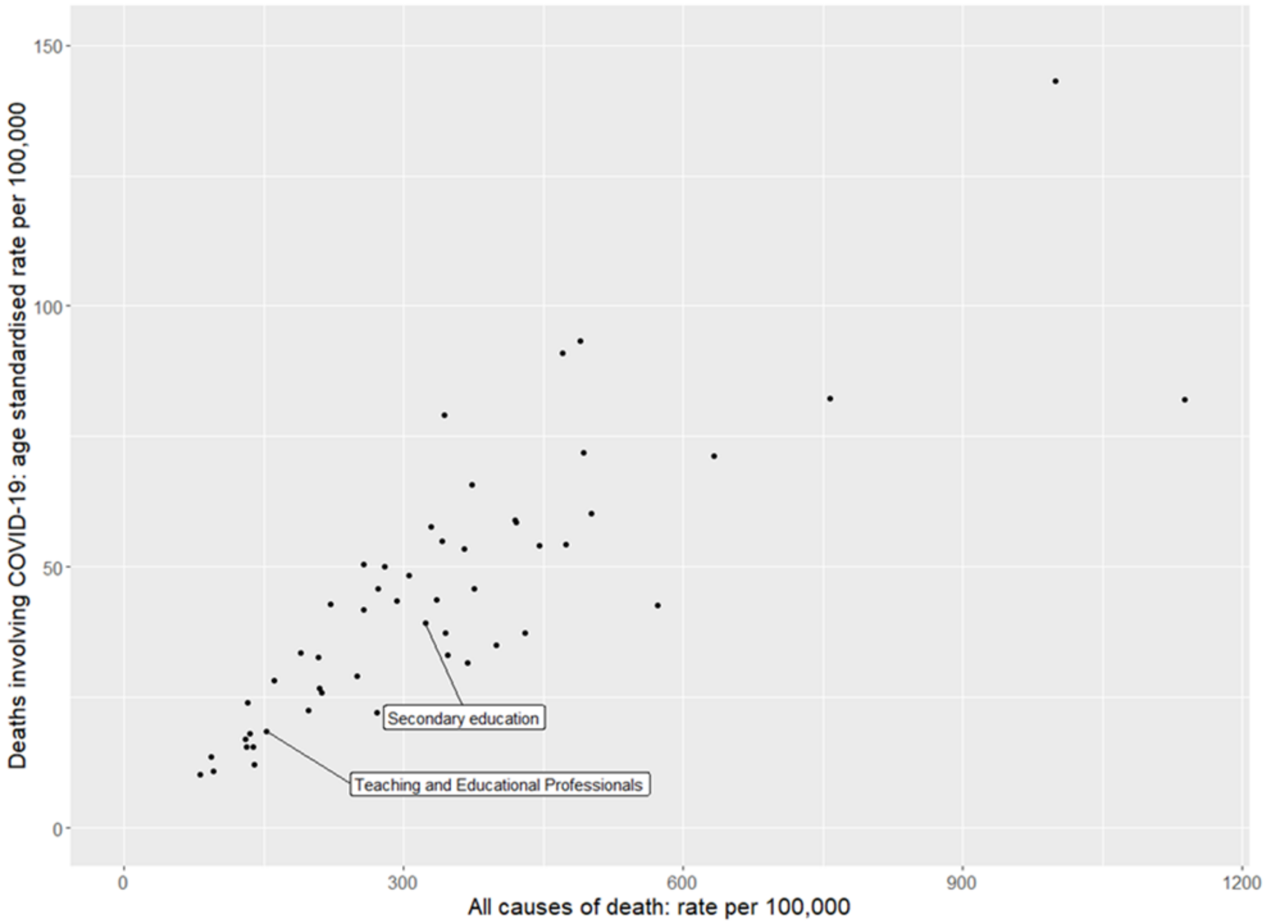

Pearson's correlation coefficient = 0.83, $p = 8.2 \times 10^{-14}$

Unlabelled data points represent other occupations in Table S4

only

Figure S2 – Scatterplot of mortality rate (per 100,000) for Covid19 and all-cause mortality for women, between 9th March and 28th December 2020

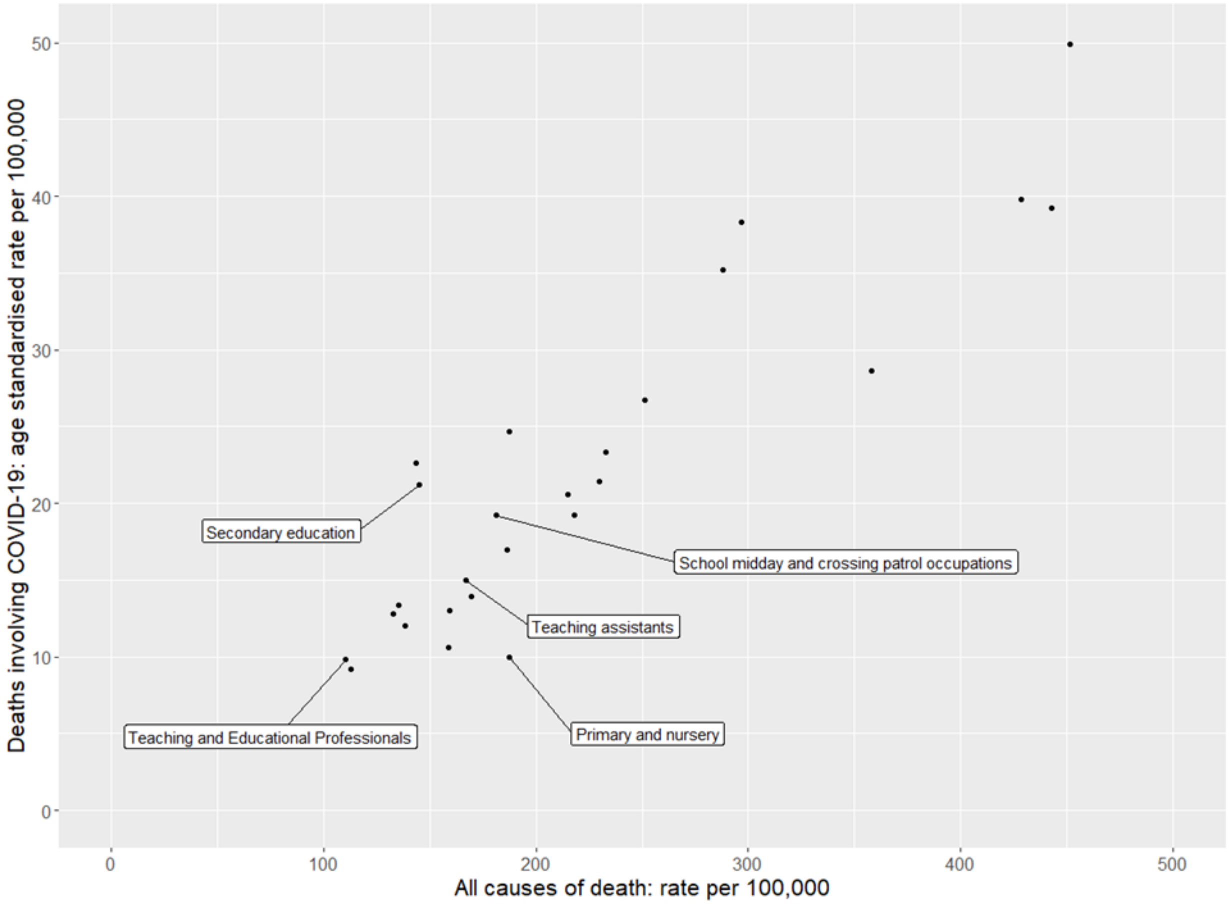

Pearson's correlation coefficient = 0.91, p = 1.4. × 10^{-10}

Unlabelled data points represent other occupations in Table S5

For peer review only

The RECORD statement – checklist of items, extended from the STROBE statement, that should be reported in observational studies using routinely collected health data.

	Item No.	STROBE items	Location in manuscript where items are reported	RECORD items	Location in manuscript where items are reported
**Title and abstract**					
	1	(a) Indicate the study's design with a commonly used term in the title or the abstract (b) Provide in the abstract an informative and balanced summary of what was done and what was found	Title (lines 2-3) and abstract (lines 14-37)	RECORD 1.1: The type of data used should be specified in the title or abstract. When possible, the name of the databases used should be included.<br><br>RECORD 1.2: If applicable, the geographic region and timeframe within which the study took place should be reported in the title or abstract.<br><br>RECORD 1.3: If linkage between databases was conducted for the study, this should be clearly stated in the title or abstract.	Title (lines 2-3) and Abstract (lines 15-19)<br><br>Reported in title (lines 1-2 and abstract 15-19)<br><br>Not applicable
**Introduction**					
Background rationale	2	Explain the scientific background and	Background (lines 54-90)		

For peer review only

		rationale for the investigation being reported			
Objectives	3	State specific objectives, including any prespecified hypotheses	Last 4 lines of the background section. (lines 93-96)		
**Methods**					
Study Design	4	Present key elements of study design early in the paper	There is a section on strength and limitations before the background (lines 39-51) and an outline of the study design in the last paragraph of the introduction (lines 90-96)		
Setting	5	Describe the setting, locations, and relevant dates, including periods of recruitment, exposure, follow-up, and data collection	This is described in the abstract and the first paragraph of the methods.		
Participants	6	*(a) Cohort study* - Give the eligibility criteria, and the sources and methods of selection of participants. Describe methods of follow-up	Not applicable- this was an ecological study using death registration data	RECORD 6.1: The methods of study population selection (such as codes or algorithms used to identify subjects) should be listed in detail. If this is not possible, an explanation	Not applicable – death registration data were used and all those eligible were represented. This is stated in the methods section and in the strength

For peer review only

		Case-control study - Give the eligibility criteria, and the sources and methods of case ascertainment and control selection. Give the rationale for the choice of cases and controls<br><br>*Cross-sectional study* - Give the eligibility criteria, and the sources and methods of selection of participants<br><br>*(b) Cohort study* - For matched studies, give matching criteria and number of exposed and unexposed<br><br>*Case-control study* - For matched studies, give matching criteria and the number of controls per case		should be provided.<br><br>RECORD 6.2: Any validation studies of the codes or algorithms used to select the population should be referenced. If validation was conducted for this study and not published elsewhere, detailed methods and results should be provided.<br><br>RECORD 6.3: If the study involved linkage of databases, consider use of a flow diagram or other graphical display to demonstrate the data linkage process, including the number of individuals with linked data at each stage.	and limitations section.
Variables	7	Clearly define all outcomes, exposures, predictors, potential confounders, and effect modifiers. Give		RECORD 7.1: A complete list of codes and algorithms used to classify exposures, outcomes, confounders, and	

For peer review only

		diagnostic criteria, if applicable.		effect modifiers should be provided. If these cannot be reported, an explanation should be provided.	
Data sources/ measurement	8	For each variable of interest, give sources of data and details of methods of assessment (measurement). Describe comparability of assessment methods if there is more than one group	Provided in the methods section, Lines 121-158.		
Bias	9	Describe any efforts to address potential sources of bias	We were unable to address this in the analysis but discuss potential bias in light of other evidence in the discussion Lines 366-403		
Study size	10	Explain how the study size was arrived at	This is described in the results section, line 196-202		
Quantitative variables	11	Explain how quantitative variables were handled in the analyses. If applicable, describe which	This is explained in the methods section under the subheading: “occupational exposure		

For peer review only

		groupings were chosen, and why	groups"- Line 121		
Statistical methods	12	(a) Describe all statistical methods, including those used to control for confounding<br><br>(b) Describe any methods used to examine subgroups and interactions<br><br>(c) Explain how missing data were addressed<br><br>(d) *Cohort study* - If applicable, explain how loss to follow-up was addressed<br><br>*Case-control study* - If applicable, explain how matching of cases and controls was addressed<br><br>*Cross-sectional study* - If applicable, describe analytical methods taking account of sampling strategy<br><br>(e) Describe any sensitivity analyses	Described under the heading "statistical methods section" in the methods section, line 160<br><br>(b), (c) and (d) are not applicable		

For peer review only

Data access and cleaning methods		..	The dataset we used is provided as reference 9, we did not carry-out any cleaning of the data.	RECORD 12.1: Authors should describe the extent to which the investigators had access to the database population used to create the study population.<br><br>RECORD 12.2: Authors should provide information on the data cleaning methods used in the study.	In Line 94 we state that this is a publicly available datset.<br><br>Not applicable
Linkage		..		RECORD 12.3: State whether the study included person-level, institutional-level, or other data linkage across two or more databases. The methods of linkage and methods of linkage quality evaluation should be provided.	Not applicable
**Results**					
Participants	13	(a) Report the numbers of individuals at each stage of the study (*e.g.*, numbers potentially eligible, examined for eligibility,	The dataset was complete or near complete due to mandatory death registration so the numbers given in the results tables	RECORD 13.1: Describe in detail the selection of the persons included in the study (*i.e.,* study population selection) including filtering based on data	Not applicable

For peer review only

		confirmed eligible, included in the study, completing follow-up, and analysed)<br><br>(b) Give reasons for non-participation at each stage.<br><br>(c) Consider use of a flow diagram	represent the total eligible population.<br><br>Not applicable	quality, data availability and linkage. The selection of included persons can be described in the text and/or by means of the study flow diagram.	
Descriptive data	14	(a) Give characteristics of study participants (*e.g.*, demographic, clinical, social) and information on exposures and potential confounders<br><br>(b) Indicate the number of participants with missing data for each variable of interest<br><br>(c) *Cohort study* - summarise follow-up time (*e.g.*, average and total amount)	a), b) and c) We do not have data on this.		
Outcome data	15	*Cohort study* - Report numbers of outcome events or summary measures over time	Numbers with the outcomes of interest are reported in Table 2.		

For peer review only

		Case-control study - Report numbers in each exposure category, or summary measures of exposure<br><br>*Cross-sectional study* - Report numbers of outcome events or summary measures			
Main results	16	(a) Give unadjusted estimates and, if applicable, confounder-adjusted estimates and their precision (e.g., 95% confidence interval). Make clear which confounders were adjusted for and why they were included<br><br>(b) Report category boundaries when continuous variables were categorized<br><br>(c) If relevant, consider translating estimates of relative risk into absolute risk for a meaningful time period	a) and b) are not applicable.<br><br>c) Absolute risks are provided in table 1 and relative risks and absolute risks are provided in Tables 3 and 4.		

Other analyses	17	Report other analyses done—e.g., analyses of subgroups and interactions, and sensitivity analyses	All analyses are reported in the paper		
**Discussion**					
Key results	18	Summarise key results with reference to study objectives	Summarised in lines 313-336		
Limitations	19	Discuss limitations of the study, taking into account sources of potential bias or imprecision. Discuss both direction and magnitude of any potential bias	Discussed in lines 364-403	RECORD 19.1: Discuss the implications of using data that were not created or collected to answer the specific research question(s). Include discussion of misclassification bias, unmeasured confounding, missing data, and changing eligibility over time, as they pertain to the study being reported.	Discussed in lines 364-400
Interpretation	20	Give a cautious overall interpretation of results considering objectives, limitations, multiplicity of analyses, results from similar studies, and	Provided in the conclusion lines 404-411		

For peer review only

		other relevant evidence			
Generalisability	21	Discuss the generalisability (external validity) of the study results	Discussed in Lines 385-387		
**Other Information**					
Funding	22	Give the source of funding and the role of the funders for the present study and, if applicable, for the original study on which the present article is based	Provided under the heading "funding" line 421.		
Accessibility of protocol, raw data, and programming code		..	A reference and a link to the raw data are provided.	RECORD 22.1: Authors should provide information on how to access any supplemental information such as the study protocol, raw data, or programming code.	A supplementary material section is provided.

*Reference: Benchimol EI, Smeeth L, Guttmann A, Harron K, Moher D, Petersen I, Sørensen HT, von Elm E, Langan SM, the RECORD Working Committee. The REporting of studies Conducted using Observational Routinely-collected health Data (RECORD) Statement. *PLoS Medicine* 2015; in press.

*Checklist is protected under Creative Commons Attribution (CC BY) license.

For peer review only
