# Supplementary material for: Was the risk of death among the population of teachers and other school workers in England and Wales due to COVID-19 and all causes higher than other occupations during the pandemic in 2020? An ecological study using routinely collected data on deaths from the Office for National Statistics
Source: BMJ Open. 2021 Nov 23;11(11):e050656. doi: 10.1136/bmjopen-2021-050656 (PMC8613671; doi:10.1136/bmjopen-2021-050656)
Supplement: Supplementary file 1 [file bmjopen-2021-050656supp001.pdf]

Supplementary material

Table S1 shows a breakdown of the number of deaths by occupation among those working in schools aged 65 and older. Excess deaths and percentage of excess deaths explained by Covid19 were calculated as for Table 2 in the main manuscript. This age group had much higher numbers of deaths compared with their 5-year average than the younger age group. Amongst all males in this age group there were 74% more deaths and amongst females there were 37% more than the 5-year average. However, only around a third of the excess deaths were thought to involve Covid19. The group of all professionals and of all occupations had fewer excess deaths as a proportion of their 5-year average but a higher proportion of the deaths involved Covid19 compared with those working in schools.

We did not have the denominators (total number working in each occupational group) for the figures presented in Tables 1s and 2s, so we were unable to work out the uncertainty in these estimates, and this will be very large in some of the smaller occupational groups**.**

Comparison of risk of death with Covid19 and from all causes for education professionals versus all professionals

Table S2 shows relative risk of deaths occurring with Covid19 for educational professionals aged 20-64 years compared to all professionals, between 9th March and 28th December 2020. Risk of dying with Covid19 in males varied from 11.5 (95%CI 5.2 to 21.7) in 100,000 for professionals in higher education to 39.2 (95%CI 24.3 to 58.6) in 100,000 for secondary school teachers. Similarly, risk in females ranged from 9.8 (95%CI 7.5 to 12.5) in 100,000 for higher education professionals to 21.2 (95% CI 12.4 to 33.2) in 100,000 among secondary school teachers. Compared with all professionals there was strong evidence that the risk of dying with Covid19 was elevated for male (RR 2.23, 95%CI 1.54 to 3.22) and female (RR 1.66, 95%CI 1.09 to 2.51) secondary school teachers. There was also evidence that the risk of dying with Covid19 was reduced among female higher education professionals (RR 0.77, 95%CI 0.60 to 0.97). For other analyses the confidence intervals were wide, and it was not possible to draw firm conclusions, although there was a suggested reduction in risk for female primary school teachers and male further education teachers.

Table S3 shows relative risks for all-cause mortality for educational professionals aged 20-64 years compared to all professionals, between 9th March and 28th December 2020. Both Male and Female higher education professionals had lower risks of mortality compared with the group of all professionals. However, there was very strong evidence that primary and secondary school teachers had higher risk of mortality from all causes compared with all professionals. This was especially true for males; male secondary school teachers had a 2.5 fold increased risk of mortality compared with all professionals and male primary school teachers a 1.8 fold increased risk.

Ratio of mortality with Covid19 versus other causes

We estimated the mortality due to all causes except Covid19 (Age adjusted all-cause mortality minus mortality with Covid19) and calculated the ratio of Covid19 mortality to mortality from other causes for minor occupational groups (according to ONS) and also for secondary school teachers and female primary school teachers. These figures are shown in Tables S4 and S5.

Table S4 shows the age adjusted mortality rates per 100,000 individuals for deaths with Covid19, plus our estimate of the non-Covid19 mortality per 100,000 among men. The ratio of deaths with Covid19 to other causes of death, ranged from 1 to 3 among male nurses and midwifes to 1 to 13 among elementary construction occupations. The ratio for male secondary school teachers was 1 death with Covid19 to 7 other deaths, which was similar to that for all education professionals.

Table S5 shows the age adjusted mortality rates per 100,000 individuals for deaths with Covid19, plus our estimate of the non-Covid19 mortality per 100,000 among women. The ratio of deaths with Covid19 to other causes of death, ranged from 1 to 5 among welfare professionals (social workers and probation officers) to 1 to 18 among primary and nursery school teachers. The ratio for female secondary school teachers was 1 death with Covid19 to 6 other deaths. Therefore, Covid19 was less likely to be a factor in deaths among female primary and nursery school teachers than other professions and Covid19 was more likely to primary and nursery school teachers than other professions and more likely to be involved in deaths among females secondary school teachers than among other professions except probation officers and social workers. For female teaching assistants the ratio of mortality with covid to other causes was 1:10 for school midday and crossing patrol occupations this was 1:8.

Note: There were fewer occupational groups with useable data (sufficient numbers of deaths to have age-adjusted mortality risks) for women than for men, this is due to small numbers of deaths in some groups among women.

Table S6- We calculated the proportionate mortality rates for occupational groups working in schools for which we had sufficient numbers of Covid19 deaths (n>10). This was calculated by dividing the number of deaths involving Covid19 for the occupational group by the total number of deaths in that group. We also calculated the SE of the proportion and from that the confidence intervals for the proportion using standard formula.

We found that male secondary school teachers had a proportionate mortality rate which was very similar to all males currently in work. We found that female teaching assistants and females lunchtime assistants had proportionate mortality rates which were similar to all other females currently in work. Female secondary school teachers had a proportionate mortality rate for Covid19 which was around 50% higher than for all female workers but the confidence intervals around this estimate were wide and overlapped with the estimate for all other workers. The proportionate mortality rate for female primary school teachers was 40% lower than for all female workers and the confidence intervals for this estimate did not overlap with those for all female workers.

Figure S1 and **S2** show scatterplots of mortality with Covid19 versus mortality from all causes for minor occupational among men (1s) and among women (2s). There was a very strong correlation between mortality with Covid19 and all-cause mortality among both men and women, correlation coefficients were 0.83 and 0.91 among men and women respectively with very small p-values. Risk of both Covid19 mortality and all-cause mortality among teachers and school staff were lower end of the scale. Male secondary school teachers fell within the centre of the plot, as did female teaching assistants and school midday and crossing patrol occupations, suggesting that the Covid19 risk reflects their normal mortality risk. However, both female primary and nursery school teachers and female secondary school teachers were slight outliers but in opposite directions, female primary and nursery school teachers had a low Covid19 risk relative to their all-cause mortality risk and female secondary school teachers had a slightly higher Covid19 risk relative to their all-cause mortality risk.

Table S1 Number of deaths among over 65s who were working in education in England and Wales 9th March to 28th December 2020

Description	Men						Women					
	Deaths involving COVID-19	All causes of death	All causes 5 year average	Excess	% excess	Excess due to Covid19	Deaths involving COVID-19	All causes of death	All causes 5 year average	Excess	% excess	Excess due to Covid19
Secondary education teaching professionals	77	565	327	238	73%	32%	19	306	226	80	35%	24%
Primary and nursery education teaching professionals	10	76	45	31	69%	32%	52	709	495	214	43%	24%
Special needs education teaching professionals	4	30	7	23	329%	17%	6	60	40	20	50%	30%
Teaching and other educational professionals n.e.c.	9	49	29	20	69%	45%	11	98	60	38	63%	29%
School secretaries	1	3	3	0	0%	NA	13	99	88	11	13%	118%
Teaching assistants	1	14	12	2	17%	50%	31	268	221	47	21%	66%
Educational support assistants	1	6	5	1	20%	20%	13	64	48	16	33%	81%
School midday and crossing patrol occupations	4	13	6	7	117%	57%	46	325	231	94	41%	49%
All those working in schools	107	756	434	322	74%	33%	191	1929	1409	520	37%	37%
All professional occupations	637	4504	3273	1231	38%	52%	401	3480	2712	768	28%	52%
All occupations	7033	43640	34676	8964	26%	78%	2993	25277	19842	5435	27%	55%

Table S2 - Covid19 related deaths for educational professionals aged 20-64 years compared to all professionals, between 9th March and 28th December 2020

Description	Men					Women				
	Risk	Risk for all professionals	RR	95% CI	P-value	Risk	Risk for all professionals	RR	95% CI	P-value
All educational professionals	0.00018	0.00018	1.05	0.82 - 1.34	0.74	0.000098	0.00013	0.77	0.6 - 0.97	0.03
Higher education teaching professionals	0.00012	0.00018	0.65	0.35 - 1.22	0.18	NA[1]				
Further education teaching professionals	0.00025	0.00018	1.40	0.75 - 2.62	0.29	NA[1]				
Secondary education teaching professionals	0.00039	0.00018	2.23	1.54 - 3.22	0.000025	0.00021	0.00013	1.66	1.09 - 2.51	0.017
Primary and nursery teaching professionals	NA[1]					0.00010	0.00013	0.78	0.49 - 1.23	0.29
[1]Less than 10 deaths, rate not reported in ONS data.										

Table S3 – All cause mortality for education professionals aged 20-64 years compared to all professionals, between 9th March and 28th December 2020

Description	Men					Women				
	Risk	Risk for all professionals	RR	95% CI	P-value	Risk	Risk for all professionals	RR	95% CI	P-value
All educational professionals	0.0015	0.0013	1.18	1.08 - 1.28	0.00023	0.0011	0.0012	0.92	0.85 - 0.98	0.015
Higher education teaching professionals	0.00095	0.0013	0.73	0.58 - 0.92	0.007	0.00073	0.0012	0.61	0.46 - 0.81	0.00059
Further education teaching professionals	0.0017	0.0013	1.29	1.01 - 1.65	0.039	0.00096	0.0012	0.80	0.62 - 1.04	0.092
Secondary education teaching professionals	0.0032	0.0013	2.48	2.18 - 2.81	1.41×10^{-40}	0.0015	0.0012	1.21	1.03 - 1.41	0.018
Primary and nursery teaching professionals	0.0024	0.0013	1.84	1.29 - 2.61	0.00077	0.0019	0.0012	1.56	1.40 - 1.74	7.77×10^{-15}

Table S4 - Covid19 versus mortality from all other causes in men by minor occupational group

Group	Covid19 mortality rate	All other causes mortality rate	Ratio
Administrative Occupations: Finance	45.9	226.4	1:4.9
Administrative Occupations: Government and Related Organisations	58.6	362.2	1:6.2
Administrative Occupations: Records	33.5	155.6	1:4.6
Architects, Town Planners and Surveyors	12.1	127.8	1:10.6
Artistic, Literary and Media Occupations	22.1	248.9	1:11.3
Assemblers and Routine Operatives	32.6	175.5	1:5.4
Building Finishing Trades	42.6	529.6	1:12.4
Business, Finance and Related Associate Professionals	15.4	116.5	1:7.6
Business, Research and Administrative Professionals	13.5	79.9	1:5.9
Caring Personal Services	91	379.3	1:4.2
Construction and Building Trades	37.3	392.5	1:10.5
Construction Operatives	31.6	337.4	1:10.7
Customer Service Occupations	42.9	178.3	1:4.2
Electrical and Electronic Trades	37.3	307.5	1:8.2
Elementary Administration Occupations	54.9	286.8	1:5.2
Elementary Cleaning Occupations	54.3	419.8	1:7.7
Elementary Construction Occupations	82.1	1055.7	1:12.9
Elementary Process Plant Occupations	143.2	856.1	1:6.0
Elementary Security Occupations	93.4	396	1:4.2
Elementary Storage Occupations	54	391.4	1:7.2
Engineering Professionals	10.8	84.7	1:7.8
Functional Managers and Directors	10.3	71.3	1:6.9
Health Professionals	23.9	108.9	1:4.6
Housekeeping and Related Services	29.1	220.4	1:7.6
Information Technology and Telecommunications Professionals	15.6	122.7	1:7.9
Information Technology Technicians	26.8	183.5	1:6.8
Leisure and Travel Services	48.4	257.7	1:5.3
Managers and Directors in Retail and Wholesale	28.3	133.3	1:4.7
Managers and Directors in Transport and Logistics	50	230.3	1:4.6
Managers and Proprietors in Hospitality and Leisure Services	72	421.3	1:5.9
Managers and Proprietors in Other Services	43.5	248.8	1:5.7
Metal Machining, Fitting and Instrument Making Trades	33.2	313.6	1:9.4
Mobile Machine Drivers and Operatives	35.1	363.9	1:10.4
Nursing and Midwifery Professionals	79.1	264.2	1:3.3
Other Administrative Occupations	25.9	185.8	1:7.2
Other Drivers and Transport Operatives	43.7	292.3	1:6.7
Other Elementary Services Occupations	59	360.7	1:6.1
Other Skilled Trades	45.8	329.4	1:7.2
Plant and Machine Operatives	82.3	674.8	1:8.2
Process Operatives	65.7	307.4	1:4.7

Production Managers and Directors	18.1	117.4	1:6.5
Protective Service Occupations	71.2	561.9	1:7.9
Public Services and Other Associate Professionals	17	113.3	1:6.7
Road Transport Drivers	57.8	271.2	1:4.7
Sales Assistants and Retail Cashiers	53.5	312.1	1:5.8
Sales Related Occupations	41.7	215.3	1:5.2
Sales, Marketing and Related Associate Professionals	22.5	175.5	1:7.8
**Secondary education**	**39.2**	**283.8**	**1:7.2**
Teaching and Educational Professionals	18.4	135	1:7.3
Vehicle Trades	60.2	441.3	1:7.3
Welfare Professionals	50.5	206.5	1:4.1

Mortality rates are per 100,000 individuals

Table S5 - Covid19 versus mortality from all other causes in women by minor occupational group

Group	Covid19 mortality rate	All other causes mortality rate	Ratio
Administrative Occupations: Finance	13	146.2	1:11.2
Administrative Occupations: Government and Related Organisations	19.2	198.8	1:10.4
Assemblers and Routine Operatives	39.2	403.8	1:10.3
Caring Personal Services	38.3	258.5	1:6.7
Childcare and Related Personal Services	12.8	120	1:9.4
Customer Service Occupations	13.4	121.6	1:9.1
Elementary Cleaning Occupations	20.6	194.5	1:9.4
Elementary Process Plant Occupations	49.9	401.6	1:8.0
Elementary Security Occupations	17	169.2	1:10.0
Food Preparation and Hospitality Trades	28.6	329.2	1:11.5
Hairdressers and Related Services	39.8	388.8	1:9.8
Managers and Directors in Retail and Wholesale	26.7	224.3	1:8.4
Managers and Proprietors in Hospitality and Leisure Services	35.2	252.8	1:7.2
Managers and Proprietors in Other Services	13.9	155.4	1:11.2
Nursing and Midwifery Professionals	24.7	162.7	1:6.6
Other Administrative Occupations	12	126.5	1:10.5
Other Elementary Services Occupations	21.4	208.5	1:9.7
**Primary and nursery**	**10**	**177.5**	**1:17.8**
Sales Assistants and Retail Cashiers	23.3	209.6	1:9.0
**Secondary education**	**21.2**	**123.8**	**1:5.8**
**School midday and crossing patrol occupations**	**19.2**	**161.9**	**1:8.4**
Secretarial and Related Occupations	10.6	148.1	1:14.0
Teaching and Educational Professionals	9.8	100.2	1:10.2
**Teaching assistants**	**15**	**151.8**	**1:10.1**
Welfare and Housing Associate Professionals	9.2	103.3	1:11.2
Welfare Professionals	22.6	120.9	1:5.3

Mortality rates are per 100,000 individuals

Table S6- Proportionate mortality rates for those working in schools

	**Occupation**	**Covid19 deaths**	**All deaths**	**Proportionate mortality rate**
Men	Secondary teachers	29	241	0.12 (0.08 to 0.16)
	All other workers	4159	33373	0.12 (0.12 to 0.13)
Women	Primary teachers	19	327	0.06 (0.03 to 0.08)
	Secondary teachers	23	156	0.15 (0.10 t0 0.20)
	Teaching assistants	37	396	0.09 (0.07 to 0.12)
	Midday occupations	18	169	0.11 (0.06 to 0.15)
	All other workers	1669	17623	0.10 (0.09 to 0.10)

Proportionate mortality rate is number of Covid19 deaths divided by the number of deaths from all causes.

Figure S1 – Scatterplot of mortality rate (per 100,000) for Covid19 and all-cause mortality for men, between 9th March and 28th December 2020

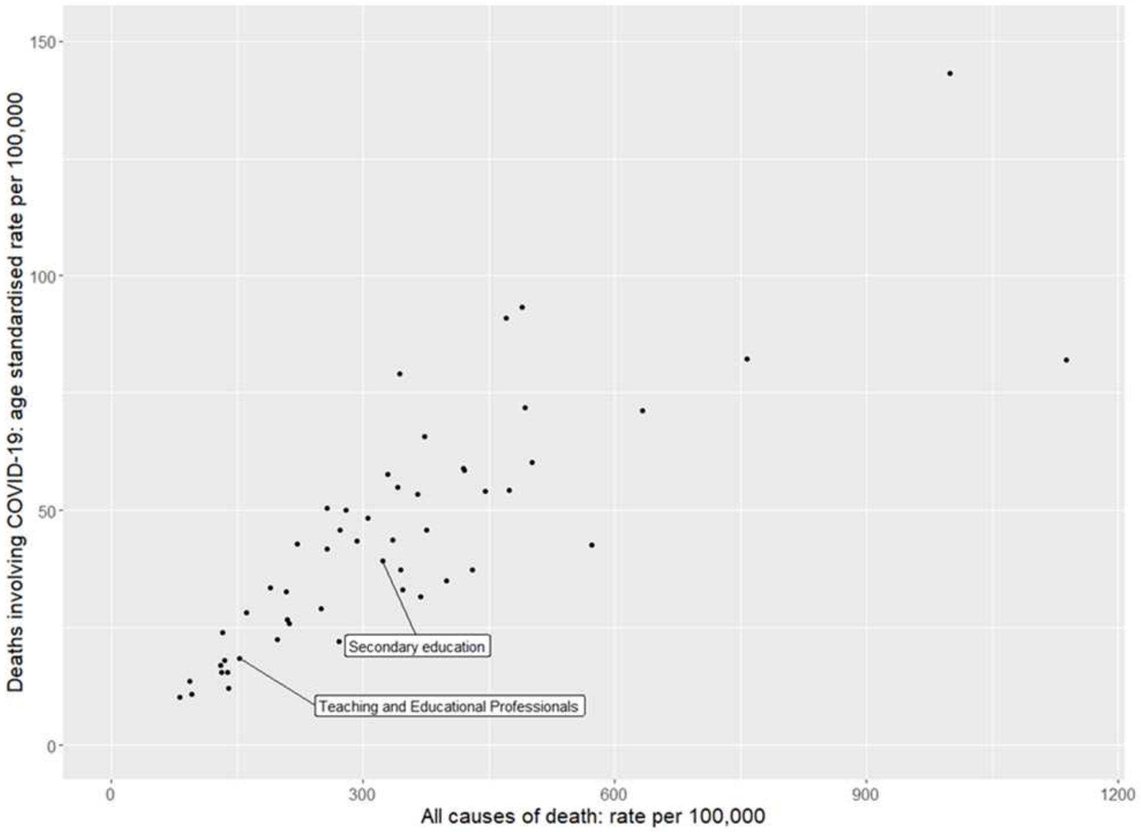

Pearson's correlation coefficient = 0.83, p = 8.2 × 10^{-14}

Unlabelled data points represent other occupations in Table S4

Figure S2 – Scatterplot of mortality rate (per 100,000) for Covid19 and all-cause mortality for women, between 9th March and 28th December 2020

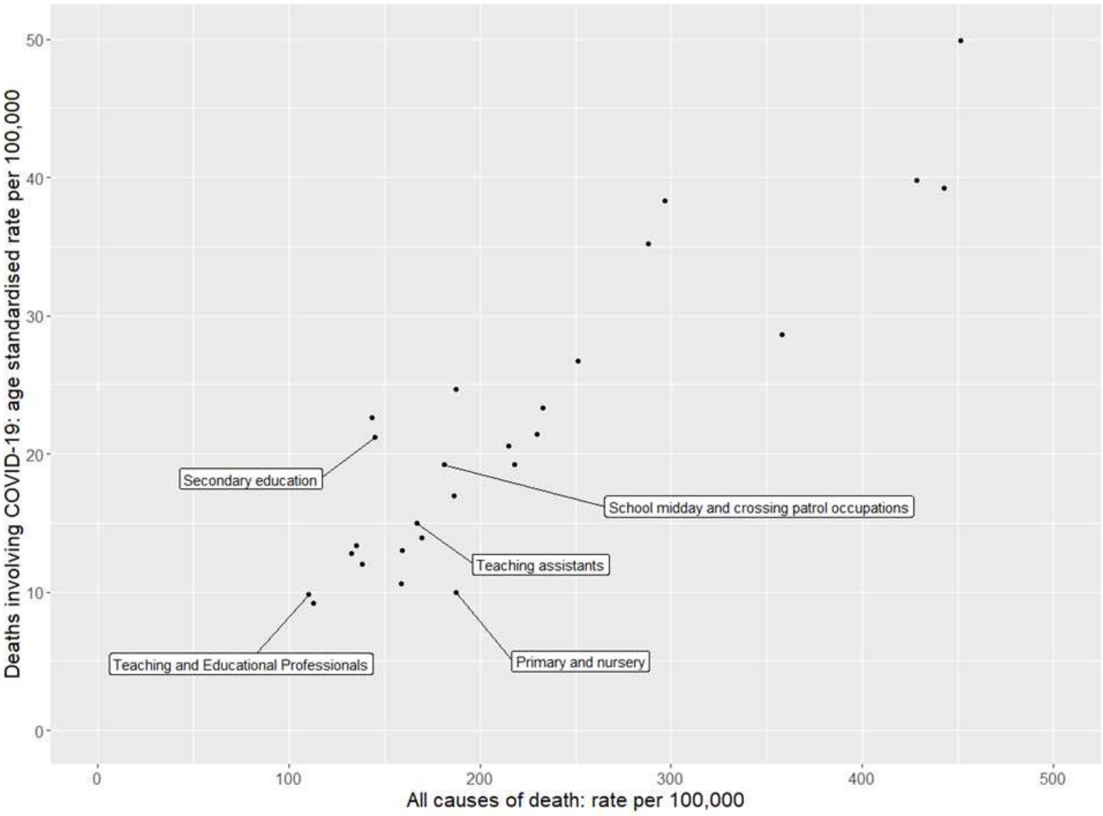

Pearson's correlation coefficient = 0.91, $p = 1.4. \times 10^{-10}$

Unlabelled data points represent other occupations in Table S5
